# Glucose exchange parameters in a subset of physiological conditions†

**DOI:** 10.1039/d3cp01973j

**Published:** 2023-08-01

**Authors:** J. Mareš, J. Karjalainen, P. Håkansson, S. Michaeli, T. Liimatainen

**Affiliations:** a NMR Research Unit, Faculty of Science, University of Oulu P.O. Box 3000, FIN-90014 Finland jiri.mares@iki.fi; b Research Unit of Health Sciences and Technology, Faculty of Medicine, University of Oulu Finland; c Department of Radiology, Oulu University Hospital Oulu Finland; d Center for MR Research, Radiology Department, University of Minnesota Minneapolis MN55455 USA

## Abstract

The chemical exchange of labile protons of the hydroxyl groups can be exploited in a variety of magnetic resonance experiments to gain information about the groups and their physicochemical environment. The exchangeable –OH protons provide important contributions to the *T*_2_ of water signals thus contributing to the *T*_2_-weighted contrast of MRI images. This exchange can be exploited more specifically and sensitively in chemical exchange saturation transfer (CEST) or longitudinal rotating frame relaxation (*T*_1,ρ_) experiments. Since glucose is omnipresent in living organisms, it may be seen as a rather universal probe. Even though the potential was first recognized many years ago, practical use has remained scarce due to numerous challenges. The major limitation is the rather low glucose concentration in most tissues. The other obstacles are related to multiple dependencies of the exchange parameters, such as temperature, pH, and concentration of various ions that are not known in sufficient detail for glucose. Thus, we embarked on evaluating the exchange parameters of a model that included every relevant chemical site for all –OH protons in both dominant enantiomers of glucose. We have (1) obtained conventional one-dimensional proton NMR spectra of glucose solutions in suitable temperature ranges, (2) we have iterated through several exchange models with various degrees of freedom determined by the number of distinguishable –OH proton sites and compared their performance, (3) we extrapolated the parameters of the best model of physiological temperature and (4) we demonstrated the use of the parameters in virtual experiments. As the main results, (1) we have obtained the temperature dependence of exchange parameters with reliable confidence intervals in three different pH values, with two of them reaching physiological temperature, and (2) we show how the parameters can be used in virtual experiments, helping to develop new applications for glucose as an NMR/MRI probe.

## Abstract 2

The chemical exchange of labile protons of the hydroxyl groups can be exploited in a variety of magnetic resonance experiments to gain information about the groups and their physicochemical environment.

## Graphical abstract

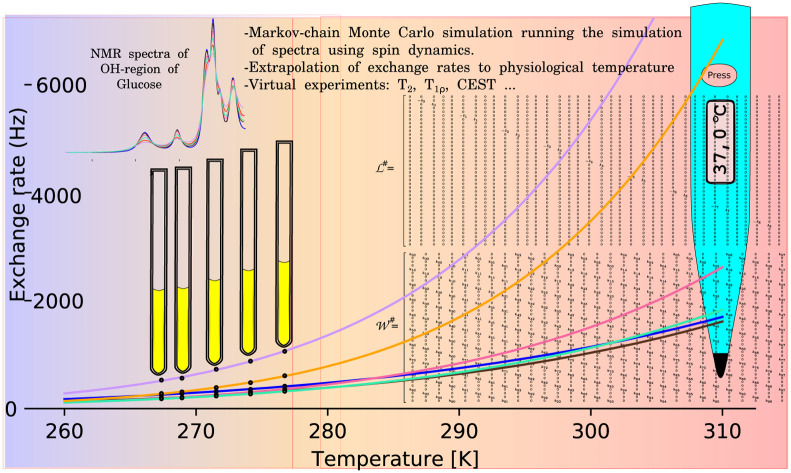

## 1 Introduction

Glucose plays an important role in the living world. It has been shown to be involved in the temporary storage of energy, transportable *via* blood and other body fluids in animals. In plants, it is the major intermediate product of photosynthesis, and precursor of many polysaccharides. Therefore, the majority of the energy entering the biosphere flows through this molecule.^1^ In humans, blood usually contains a relatively stable concentration of glucose of around 5 mM, transporting energy to muscles, brain and other organs. Abnormal glucose concentrations are associated with pathologies, such as diabetes or tumours. It is therefore of great interest to be able to measure the glucose concentration in different parts of the body, preferably noninvasively. In plants, monitoring of glucose production can be used to assess plant health which has in turn large agricultural importance, as seen from studies using different techniques.^2^ Application can also be expected in animal production, such as salmon farming.^3^ In order to measure the spatial distribution of glucose in the living body, the exchanging protons of the –OH sites can be used to create a natural contrast. The chemical exchange creates various effects in NMR, and these can be observed using *e.g.*, *T*_2_ or *T*_1,ρ_ relaxation measurements, or chemical exchange saturation transfer (CEST) experiments. The results of these experiments are mainly dependent on the concentration of glucose as well as the exchange rates of the glucose –OH groups. The exchange rates in turn depend on the properties of the solution, such as pH, concentration of different ions and temperature. Chemical exchange can also be used in signal enhancement through various hyperpolarisation techniques, see, *e.g.* ref. 4. There, hyperpolarisation using Xe atoms is well researched.^5^

The role of glucose in assessing clinical conditions in MRI has been mostly limited to academic discussions. Its potential in creating MRI contrast through its exchange properties is used in CEST-based MRI.^6,7^ The so-called Gluco-CEST has been proposed for the study of glucose concentration or as a pH-sensitive biomarker.^6,7^ Measurements of glucose in tumours have also been the aim of many other studies.^7–11^

The sensitivity of the –OH exchange rate with respect to pH can be used in principle for the measurement of pH.^12^ There are also challenges in using the –OH groups of glucose. One challenge is associated with the low concentration of glucose in blood, and the concentration of glucose in other tissues is usually even lower. This can be elevated by various means, but to a rather limited extent. One option has been to introduce a glucose derivative with a reduced metabolic rate,^13,14^ accumulating larger concentrations in tissues, with a risk of toxicity. Similarly, derivatives of disaccharides, sucralose and maltitol, used as artificial sweeteners, have been used in brain tumour imaging in rat models.^15,16^

Another problem is related to the small chemical shift of glucose –OH groups with respect to water. This challenge is more apparent at low magnetic fields of clinical MRI instruments, where the glucose –OH resonances cannot be easily targeted in CEST experiments without obscuring the results by irradiating the water resonance as well. The challenges of glucose-CEST in 3T have been extensively investigated in recent studies.^17,18^ Both drawbacks of the glucose properties are being alleviated at higher magnetic fields. To be able to evaluate the potential of glucose as a biomarker for MRI in clinical studies, it is crucial to know its properties accurately. Only then can valid conclusions be drawn from simulations of CEST spectra under realistic conditions, including a slightly inhomogeneous magnetic field, temperature, pH, or diffusion. It is important to know the details of the chemical shift values of the –OH protons with respect to water protons, and their exchange rate as a function of temperature, pH, and possibly other factors, such as concentrations of ions acting as proton exchange catalysts.^19^ The proton chemical exchange rates have been previously determined with various validity ranges.^17,18,20,21^ In the case of glucose, there has been a great uncertainty about the exchange rates and chemical shifts of individual hydroxyl protons, as well as their dependence on pH. This is mostly due to the fact that the exchange is in the intermediate-to-fast regime, and therefore it is detectable only indirectly under physiological conditions, as the –OH peaks coalesce into a (broadened) water peak. To properly evaluate these measurements or assess the feasibility of new techniques, it would be invaluable to have a detailed model of exchange, accurately parameterized, with known dependencies on all the properties of the solution. The confidence intervals of the measured parameters are also of critical importance. Such a comprehensive set of parameters is not available in the literature. Here, we build a model including every possible chemical site for all relevant –OH protons in both dominant enantiomers of the glucose in solution.

In this study, we started by measuring glucose solutions in standard phosphate buffered saline (1× PBS^22^) at three pH points and at temperature ranges suitable for resolving at least a subset of –OH proton sites for a given pH. We then tested several exchange models, including various numbers of distinct exchange sites and their interdependency. The parameters of the models together with error margins were estimated using Markov chain Monte Carlo simulation, which also allowed us to choose the most suitable exchange model. Taking the temperature dependence of the best-model parameters, we could extrapolate the exchange parameters to physiological temperature for a subrange of physiological pH values.

In this study, we investigate the dependence of the exchange parameters of the –OH protons of glucose on temperature and pH. In addition to pH, the chemical composition is kept constant, close to the physiological state, by using the standard 1× PBS buffer. We prepared samples of 1 M glucose in 1× PBS and adjusted the pH to three different values. To measure the exchange parameters, *i.e.*, exchange rates and chemical shifts, of each –OH site, we measured the temperature series of standard proton NMR spectra. The temperature ranges were selected after the initial screening, such that the highest temperature still contains resolution, allowing us to visually distinguish at least subsets of –OH signals. It was assumed that the –OH region of the spectrum is determined by the chemical exchange. To obtain the exchange parameters, a suitable model was gradually selected. The model consisting of distinct chemical shift sites and exchange rates is used to simulate the spectrum, which in turn enables finding the exchange parameters during the fitting procedure. We have therefore decided to employ Markov chain Monte Carlo (MCMC)^23^ simulation to facilitate the fitting.

## 2 Materials and methods

### 2.1 NMR experiments

Glucose samples were prepared by dissolving a known amount of glucose in 1× PBS buffer to obtain a concentration of 1 M. The pH was adjusted by a minimal amount (a few μl) of concentrated HCl or NaOH at room temperature, measured by a calibrated pH meter. Three samples were prepared with pH values of 6.21, 7.00 and 7.38, to cover the expected physiological range. Measurements were performed on an 11.7 T Bruker Avance III spectrometer, in a 5 mm tube. In addition to setting the temperature by the control unit, an accurate temperature was determined using a standard Bruker MeOH-d_4_ temperature calibration sample. The spectra were measured without any lock signal using the standard flip acquisition 1D proton spectrum with 32 scans, spectral width 15 ppm with 16k time domain points. Processing was done using line-broadening of 1 Hz, without any zero-filling. Spectra were carefully phased and baseline corrected with a second-order polynomial. Only part of the spectra that included the glucose –OH protons was extracted, starting from the first minimum between the water line and the –OH signals, up to 8.5 ppm, covering approximately 3800 points. The extracted section was normalized and used for the fitting procedure. After the initial screening, the sample with pH = 6.21 was measured between 270 K and 295 K. The sample with pH = 7.00 was measured between 268.5 K and 277.5 K as the higher temperature spectra do not have sufficient resolution (see Fig. S2 in the ESI†).

### 2.2 Simulation of NMR spectra

The simulation of the spectra was carried out using a light-weight in-house written Python program, using spin-dynamics simulation in extended Liouville space, see *e.g.* J. Jeener.^24^ Briefly, the density operator, a superket in the Liouville space |*ρ*〉 is expanded using the chemical space (called also the chemical configuration vector) with fractional concentration of exchanging sites, into |*ρ*^#^〉 = |*ρ*〉⊗|*m̂*〉, where ⊗ denotes a tensor product (also called the Kronecker product when dealing with matrix representation instead of operators). Observables, such as components of spin magnetization, are expanded using unit superkets, |*A*^#^〉 = |*A*〉⊗|1̂〉. The Liouvillian is expanded similarly as *L*^#^ = *L*⊗1̂, and supplemented with an exchange superoperator *W*^#^ which is a chemical exchange (reaction) matrix *K* of first-order reactions, expanded into the size of the extended Liouville space as *W*^#^ = 1̂⊗*K*. The time evolution of |*ρ*^#^〉 can be written in the same way as for the “standard” Liouville space:1
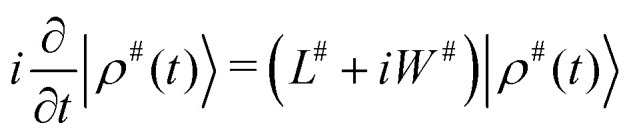
The time evolution can be facilitated by obtaining the propagator as *P*^#^(*τ*) = exp((*L*^#^ + *iW*^#^)*τ*) to obtain a next state ket *ρ*^#^(*t*) in time *τ* like:

2 |*ρ*(*τ*)〉 = *P*(*τ*)|*ρ*(0)〉,

where the superscript # was omitted to emphasize the analogy with the standard Liouville space behaviour for the situation where relaxation other than that due to chemical exchange is not taken into account. In the actual simulation, the time propagation is done in small steps to accumulate the “free induction decay” (FID) signal, which is then processed in the same way as in the standard NMR spectroscopy (see *e.g.* ref. 25).

The simulation was defined by the list of chemical shifts, exchange rates, and fractional concentrations. An example defining the simulation of the spectrum of 1 M glucose at 269.5 K is given in Table S11 of the ESI,† assembled from Tables S1, S7 and S8 of the ESI.† Only water and the exchanging –OH protons of glucose were included in the simulations of the spectra. Only the chemical-shift term of the NMR spin Hamiltonian was considered. This means that the simulation consists of isolated spins in pools with exchange, equivalent to the Bloch–McConnell equations.^26^

The spectral line widths were assumed to be determined purely by the exchange broadening, and no additional relaxation mechanisms were considered. This can be justified by the observation that the line width 
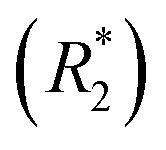
 of non-exchangeable protons of glucose is between *ca.* 15 Hz at the lowest temperature of 270 K and 7.5 Hz at 290 K. The –OH, similar to other glucose protons, do not have close-by protons acting as partners for dipolar relaxation, therefore it can be assumed that their intrinsic relaxation rate due to a combination of mechanisms such as dipole–dipole and CSA interactions would be similar, with upper ranges of 15 and 7.5 Hz for 270 and 295 K. These relaxation rates contribute to the line width, so ignoring them causes a slight overestimation of the exchange rate determined using our approach. This overestimation is gradually less important with increasing temperature, with an upper bound around 1% error at 295 K. The same simplification was used before, *e.g.* in ref. 27.

For the simulated spectra, the same parameters were used as for the experimental spectra, time-domain points, line-broadening exponential window function, and spectral width for processing. For fitting, the simulation is performed in every iteration to obtain the error as the sum of squares using the difference between the corresponding ∼3800 spectral points. To be able to directly compare the simulated and experimental spectra, the simulated spectrum was further modified by a second-order polynomial employed to simulate the baseline correction with parameters included in the fitting model. Then the section, exactly corresponding to the experimental one, is extracted and normalized. The integral of the section was left as an additional fitting parameter (with a narrow range of 0.8–1.2 multiple of the normalization constant) in the model, resulting in a total of 4 fitting parameters for the baseline.

### 2.3 Exchange models

**Fig. 1:**
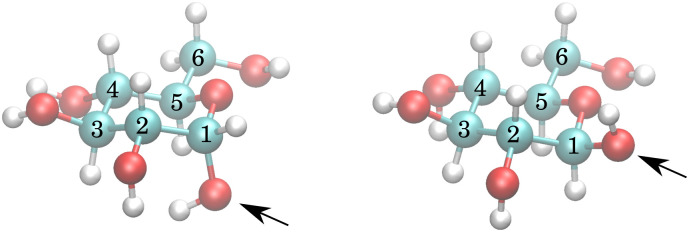
α- (left) and β- (right) anomers of glucose. The arrows point at anomeric oxygen at C_1_, determining the anomers. Notice that the hydrogens of C_1_ and C_2_ –OH groups of the α anomer are much closer to each other than for the β anomer.

**Fig. 2:**
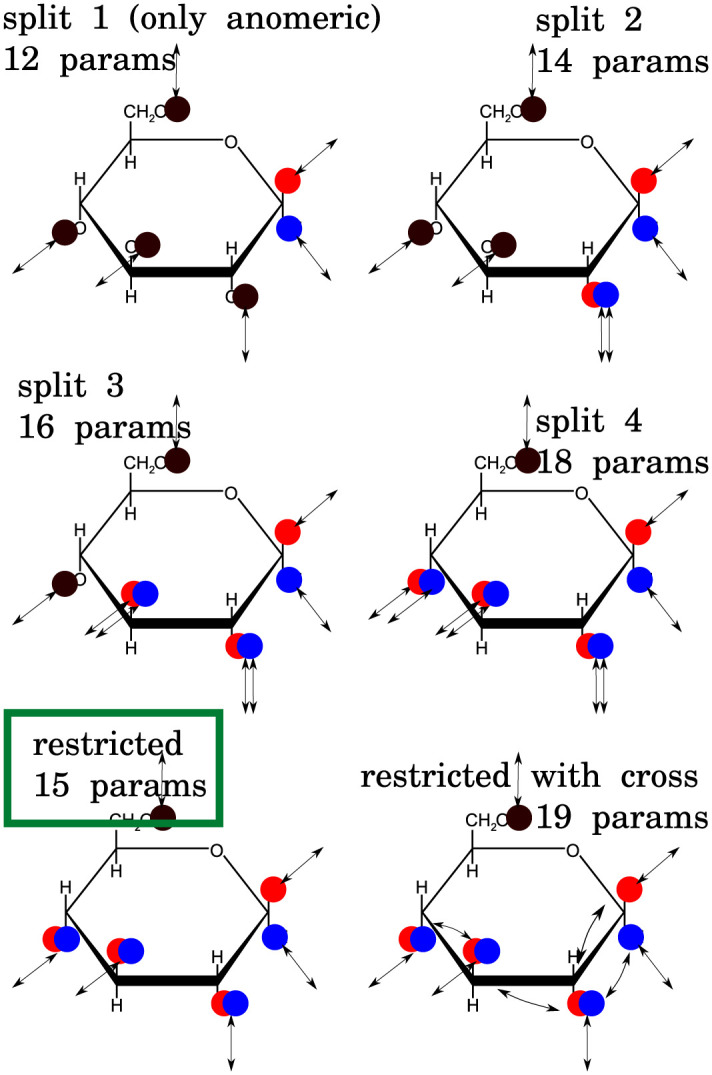
Exchange models. Each arrow indicates an independent exchange rate. Two colours of any –OH site indicate independent chemical shifts for α and β anomers. The “restricted” model was used for all final results in this work. The schemes of models “split 2” and “split 3” are slightly misleading, for example, for “split 2” in the actual calculation, there was no assignment of whether there are distinct chemical shifts and exchange rates at C_2_, C_3_ or C_4_. Clearly, many other models can be considered.

The exchange model of glucose contains all –OH sites. There are five distinct –OH groups on the glucose molecule, at C_1_, C_2_, C_3_, C_4_ and C_6_, using standard numbering (Fig. 1). The number of sites is, however, doubled due to the two major forms present in solution, the α- and β-anomers, with their relative fractional concentrations of 0.36 and 0.64.^28^ For the chemical exchange properties, it may be assumed that –OH at C_6_ would remain approximately identical to both forms. A model that should be able to describe all the parameters has therefore 18 independent parameters to determine: 9 chemical shifts and 9 exchange rates. In addition, there are four parameters of the spectrum baseline correction. We will see that not all of these parameters are independent. Moreover, it is natural to use the simplest model capable of explaining the experimental observations. The models are schematically depicted in Fig. 2.

With nine independent chemical shifts of glucose –OH groups and one for water, the Liouvillian matrix has dimensions of 40 × 40. Every spectrum, including setup, takes around 3–4 seconds to simulate.

### 2.4 Monte Carlo simulation

Since the spectra suffer from high overlap, the fitting can be trapped in many local minima. For the same reason, it is critically important to also obtain confidence intervals for the fitted parameters. In this situation, reliable estimates of the parameter values and error margins can be obtained by Markov chain Monte Carlo simulation.^23^ In this study, we used the methods implemented in ref. 29, modified and rewritten into Python language.

Briefly, in MCMC, similarly to other procedures for fitting parameters of a given model, there is a vector of experimental data *Y*_exp_, together with an error estimate such as *Y*_SEM_, which defines our prior probability distribution. The parameters of a model are defined as residing within given boundaries. Initially, each parameter is placed in a random position within its boundary. The *n* parameters are then randomly varied to sample the *n*-dimensional space. To do that, a (pseudo)random number is generated on the unit interval and multiplied by a step size *k*_*i*_, corresponding to the *i*th parameter. The product is added to the *i*th parameter and the model is reevaluated, obtaining a vector of model data *Y*_model_. The change is accepted if the error *E* with respect to *Y*_exp_ is smaller than with the unchanged parameters (Δ*E* < 0), as would be done in a standard minimization procedure. If Δ*E* > 0, the change is accepted if e^−Δ*E*^ < *u*_rnd_, where *u*_rnd_ is again a (pseudo)random number from the unit interval. In this way, the simulation samples the *n*-dimensional space. The sampling is the most efficient if the change is accepted with 50% probability. Therefore, during the initial stage of the MCMC, the step *k*_*i*_ is adjusted to obtain roughly a 50% acceptance rate. This is repeated later before a production phase, preferably when the parameters sample the higher probability region. If the parameter leaves its boundaries, it is returned within them by taking its mirror with respect to the crossed boundary. The error *E* is related to a common sum of squares *RSS*, but scaled by *Y*_SEM_ and the inverse temperature of the MCMC simulation *T*_inv_. 
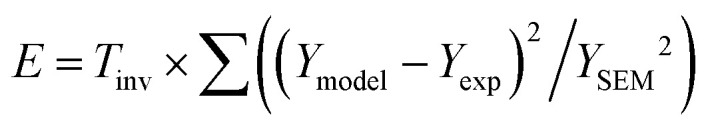
. In the favourable case, the global minimum is found during the simulation, together with the posterior distribution in a Bayesian sense, which is further processed to obtain the confidence interval of a desired 1 − α, here 95%. The distribution of a given parameter is, in general, not symmetric around the optimal value of the parameter, and therefore also the confidence interval is not symmetric. In this study, we have symmetrized the confidence intervals to comply with common practice in reporting the uncertainty. This is largely justified by the fact that the distributions are not far from symmetric (see Fig. S8 in the ESI†).

In order to obtain sufficient statistics for reliable error ranges, the length of the production part of the simulation was twenty thousand steps. The number of spectra simulations was this number multiplied by the number of parameters of a given model. One measure to estimate if the simulation is long enough can be obtained by the step-correlation, which should be small on a scale of trajectory steps. Examples of such correlation functions are shown in Fig. S9 in the ESI.† Based on those, the simulation is long enough.

For the initial fitting of the low-temperature, lower pH spectrum, the ranges of fitted parameters are shown in Table S12 of the ESI.† Unless the assignment of the peak is obvious, such as for C_1_ or C_6_, the chemical shift ranges do not determine the assignment *a priori*. The ranges of exchange rates are always kept such that they safely accommodate the true value. This has been checked for all results. For the spectra measured at higher temperatures and higher pH, the chemical shift fitting ranges were narrowed to non-overlapping ranges to fix the assignment obtained for low-temperature spectra fittings. The fitting ranges of the exchange rates were very broad, not restricting the sampled values (see Table S13 of the ESI†).

### 2.5 Temperature dependence fitting

Due to the fast-exchange regime of –OH protons exchanging with water protons at physiological temperature, we have extrapolated the rates based on measurements at lower temperatures. For pH ∼ 7.4, the physiological pH of blood,^30^ this procedure was not reliably possible. The pH of cerebrospinal fluid^31^ as well as neurons^32^ is slightly lower, about 7.3, whereas the common intracellular pH is lower than that, between 6.9 and 7.2, in pathological situations even close to 6.0.^33^ Our parameterizations at pH = 6.21 and 7.00, extrapolated to physiological temperature, are therefore relevant for *in vivo* applications.

In order to extend the exchange rates to higher temperatures, up to a physiological temperature of 310 K, we have performed one more fitting series, to obtain the temperature dependence of the exchange rates, governed by the Eyring equation. We have tabulated the exchange rates from 260 K to 310 K for two different pH values of 6.21 and 7.00. Partial parameterization has also been obtained for the third pH value of 7.38. Using the exchange parameters, we show examples of virtual NMR and MRI experiments and discuss further possible use.

For evaluation of the temperature dependence, we selected the restricted model described in Section 3.2, containing six separate exchange rates and nine chemical shifts. Extrapolation of exchange rates to higher temperatures for individual –OH sites can be obtained by fitting to the Eyring equation.^34–36^3
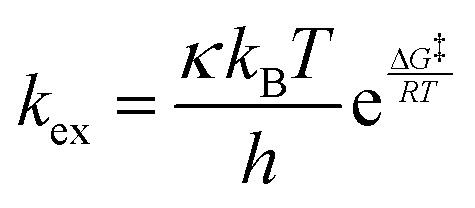
Where the reaction rate is in our application the exchange rate *k*_ex_, *κ* is called the transmission coefficient, *k*_B_ is the Boltzmann constant, *h* is Planck's constant, Δ*G*^‡^ is the Gibbs energy of activation, *R* is the universal gas constant and *T* is the absolute temperature. Both parameters (*κ*, Δ*G*^‡^) are obtained by fitting. The validity is briefly discussed in Section 4.3. As the exchange rates follow this temperature dependence very well, it allows for a safe extrapolation into regions out of measurements.

The fitting was done using the linearized form of eqn (3), which allowed us an error propagation into *κ* and Δ*G*^‡^ and subsequently into extrapolated temperature ranges. The analysis was performed using a Python library called uncertainties.^37^ The linear fit of the equation **y** ≐ *a***x** + *b* is done by combining *a* and *b* into vector *ab*, and forming a “design matrix” **x***ab*. This results in a simple matrix equation **y** ≐ **x***ab*. For actual calculation, the **x** matrix contains a column of *x* values and a second column of ones. Then *ab* ≐ **x**^−1,*p*^·**y**, where −1,*p* indicates pseudoinversion of a non-square matrix. The only mathematical operations, pseudoinversion and matrix multiplication, are directly available from the uncertainties.unumpy package. Therefore, the vector **y** can be used with its confidence intervals during calculation of *a* and *b*. Exponential and logarithmic functions, needed for linearization of the Eyring equation, are also directly available in “uncertainties”. The confidence intervals in Tables S1–S6 in the ESI,† are therefore obtained by propagation of the reliable confidence intervals of the exchange rates obtained by the MCMC simulations. They do not contain additional uncertainty due to the imperfection of these points along the linearized Eyring equation dependency. The same holds for the *κ* and Δ*G*^‡^ confidence intervals in Table 2.

## 3 Results

### 3.1 Initial selection of the model

**Fig. 3:**
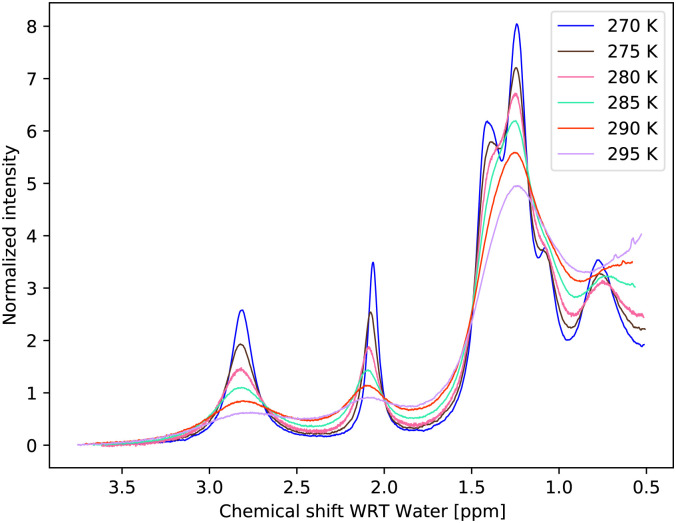
Measured temperature series of proton spectra of 1 M glucose at pH = 6.21.

**Fig. 4:**
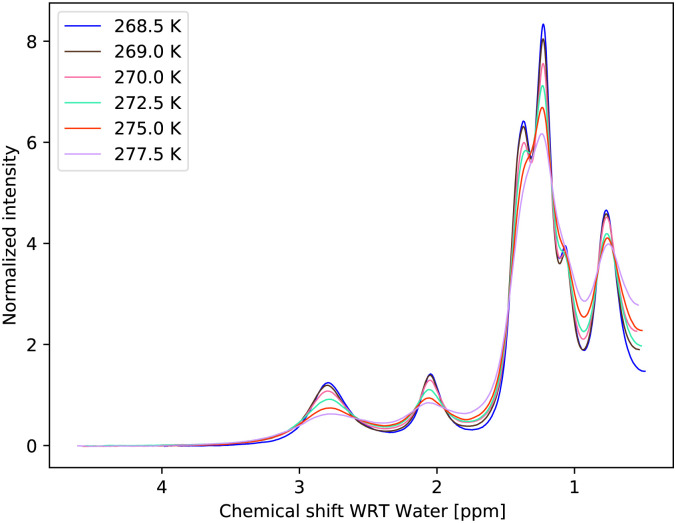
Measured temperature series of proton spectra of 1 M glucose at pH = 7.00.

The temperature series of the spectra at low pH 6.21 (Fig. 3) and at pH 7.00 of 1 M glucose in water shows that the slowest exchange rates and, moreover, the narrowest line widths were obtained with the lowest pH considered and the lowest temperature (270 K), as expected. Therefore, the initial studies were carried out using these spectra. The spectra can be seen in Fig. 3 and 4. In the case of the third sample with pH = 7.38, only spectra obtained at 270 K and 275 K were used, again, with higher temperature spectra lacking the resolution (see Fig. S3 in the ESI†).

**Fig. 5:**
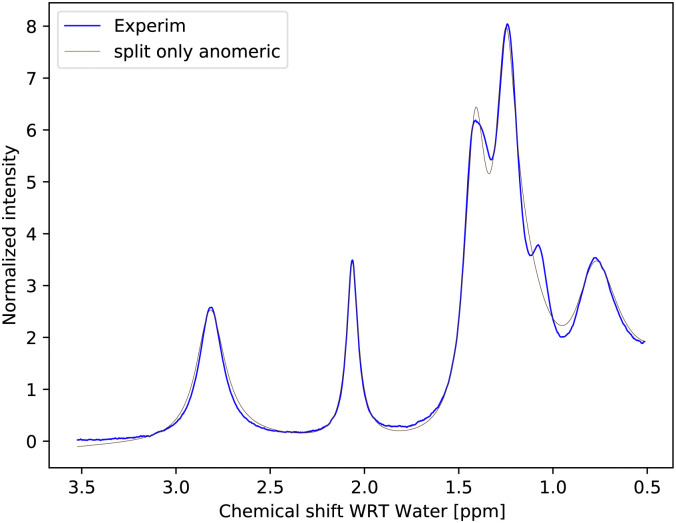
Measured spectrum of 1 M glucose at pH = 6.21 as compared with a fit to the simplest model considered, with separate chemical shifts of α and β allowed only at –OH of C_1_. This model is called “split only anomeric”. It is clear that there is at least one very distinct signal around 1.05–1.1 ppm, which, due to the proximity to C_1_, must be at C_2_ and since the volume corresponds to α, we have this one new assignment.

The simplest model with separate chemical shifts of α and β allowed only at –OH of C_1_ fails to recover one important and some minor features of the spectra, as can be seen in Fig. 5. Contrary to our original assumption, it turned out that the splitting of the –OH sites due to α- and β-anomers is not limited only to the anomeric –OH, but also influences the chemical shift of other –OH protons so significantly that this can be clearly resolved. Looking at the molecular structure of these anomers (Fig. 1), this is hardly surprising. In the case of the α-glucose, the anomeric –OH oxygen can be in the van der Waals distance from the –OH protons of carbon-2.

**Fig. 6:**
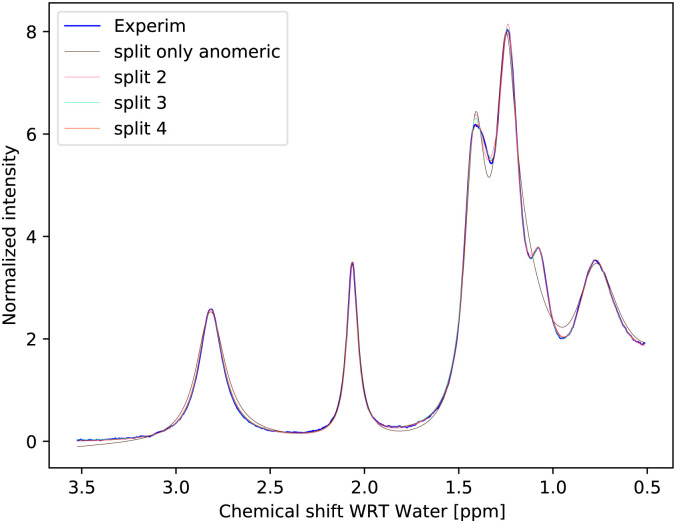
Measured spectrum of 1 M glucose at pH = 6.21 as compared with the fits to different models.

Inspection of the spectrum in Fig. 5 shows that there are other minor features within the –OH groups on the glucose ring that may also be caused by chemical shift differences of the other –OH protons at carbons 3 or 4, or both. Therefore, instead of allowing only one more –OH proton to assume different chemical shifts for α- and β-anomers, we considered also cases in which distinct chemical shifts and exchange rates of the three or four –OH protons were allowed (see the schematics in Fig. 2). The only –OH site that was not considered was that at carbon-6, for which there is no evidence of splitting from our measurements. However, this may be reconsidered in future studies where higher resolutions could be achieved. The comparison is shown in Fig. 6.

In all of these models, the exchange rates of well separated signals, the C_1,α_, C_1,β_ and the C_6_ give the same results within ∼10 Hz, whereas the exchange rates for the overlapping signals are not conclusive. This also results in poor fits of the temperature dependence to the temperature series (data not shown). This series of models, created mechanistically by adding more sites with both distinct chemical shifts and exchange rates, is therefore failing in the important goal of being sufficiently robust due to unrealistic degrees of freedom. For example, “split 2” has five glucose OH sites, of which two are split due to α and β enantiomers, as illustrated in Fig. 2. Besides OH at C_1,α_, C_1,β_, one more site is split like (1.0;*δ*_*i*_;*k*_ex,*i*_) → ([0.36,0.64];[*δ*_*i*,α_,*δ*_*i*,β_];[*k*_ex,*i*,α_,*k*_ex,*i*,β_]), where the first field 1.0 → [0.36,0.64] represents the fractional concentration of the sites. Similarly to the distinct chemical shift *δ*, the exchange rate *k*_ex_ at positions 2, 3 and 4 may differ for different enantiomers due to the long-range effect of the conformation at C_1_. But, as discussed in Section 4.5, the spectra do not contain enough information to resolve these, and as we see in the following section, it is better to assume that the *k*_ex,*i*_ for all but the C_1_ sites are the same for both enantiomers.

### 3.2 Reintroduction of restrictions to the model

In the paragraph above, we have gradually allowed for every –OH group, except C_6_, to have a distinct chemical shift and distinct exchange rate for α- and β-anomers. We may think, however, that the exchange rates would be approximately identical for C_2_–C_4_ groups in both of these anomers, even if the chemical shifts may slightly differ. We call this scenario the “restricted model”. Restricting the model in this sense will furthermore introduce constraints, allowing the fitting procedure to assign which –OH signals correspond to each other in different anomers. The hypothetical spectra of the separate α and β anomers, using the fitted parameters, are shown in Fig. S5 of ESI.† There are therefore six distinct exchange rates, for C_1,α_, C_1,β_, C_2_, C_3_, C_4_, and C_6_. Based on Fig. 9, it can be stated that this model gives enough freedom to fit the data as the fit already follows all the relevant details. The assignment of the C_2,α_ signal can be well guessed, as it is so distinct, indicating close proximity to the actual anomeric group at C_1_. The model defines the fraction corresponding to α, so only the α signal can be accommodated at that position, and the fit to a model gives the chemical shift of the corresponding β, the C_2,β_, which would be 1.258 ppm.

### 3.3 Temperature dependence

Temperature dependencies of the chemical exchange rates were parameterized with the Eyring equation^3^ at two (and partly also at a third) pH points, using the exchange rates from the MCMC fit of the restricted model. Whereas for pH = 6.21, spectra up to a temperature of 295 K could be used (Fig. 3), for pH = 7.00, we restricted the temperature to maximum of 277.5 K and measured the spectra in small steps. Spectra of the sample with pH = 7.00 with a temperature up to 295 K can be seen in the ESI,† Fig. S2, illustrating the need for the condensed temperature range.

**Fig. 7:**
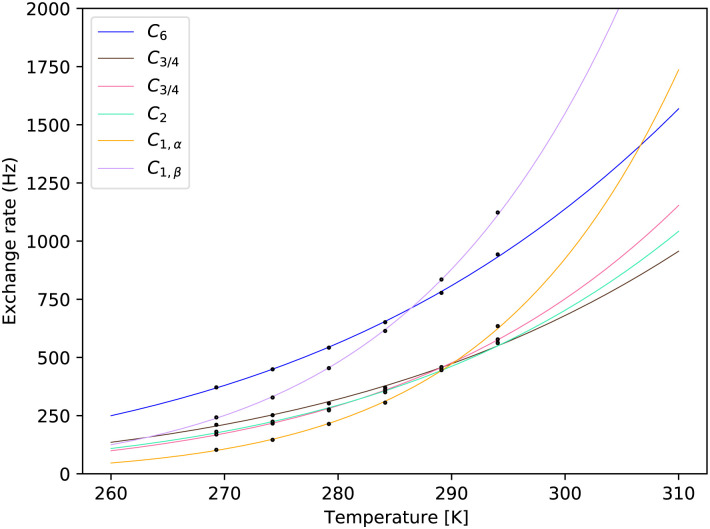
Fits of the temperature series for the sample of pH = 6.21. The exchange rates obtained by MCMC fit for each site are shown at calibrated temperatures. See further details in the ESI,† Fig. S5.

**Fig. 8:**
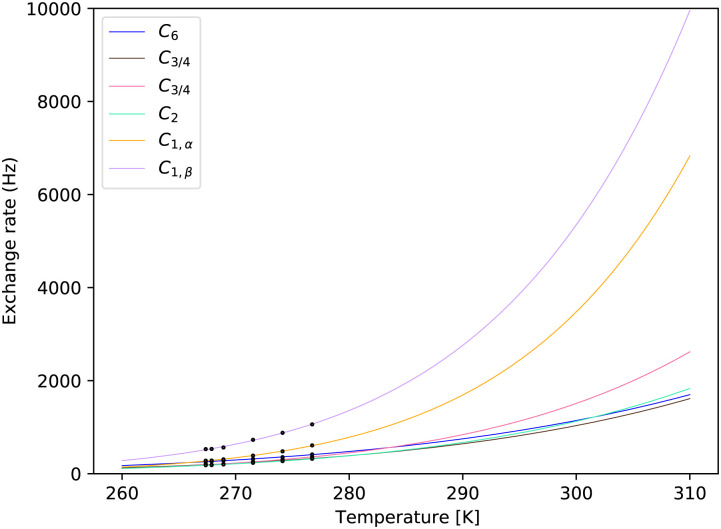
Fits of the temperature series for the sample of pH = 7.00. The exchange rates obtained by MCMC fit for each site are shown at calibrated temperatures. See further details in the ESI,† Fig. S6.

The best fits for every distinct exchange rate can be visually inspected in Fig. 7 and 8. Fig. S6 and S7 in the ESI,† show details around the measured temperatures. One can notice the steep temperature dependence of the hemiacetalic –OH at C_1_ for both α and β anomers. These are possibly the most important signals, as their large chemical shift with respect to water makes them the easiest target for CEST or off-resonance *T*_1,ρ_ experiments. Due to the largest chemical shift difference, they also have the largest impact on the *T*_2_ of the water signal. For convenience, we report tabulated values together with 95% confidence intervals in the ESI,† in the range between 260 K to 310 K with 0.25 K step. These are expected to be sufficient for most applications. As an example, we show separately parameters at the human physiological temperature of 310 K in Table 1.

**Table:** Exchange rates extrapolated to 310 K, 1 M glucose at pH = 7.00 and 6.21, using the restricted model, with partial assignment

*k*_ex_/Hz (pH = 7.00)	*δ* ppm (pH = 7.00)^a^	*k*_ex_/Hz (pH = 6.21)	*δ*/ppm (pH = 6.21)^b^	Atom
1699 ± 8	0.77576 ± 0.00018	1569 ± 10	0.763 ± 0.003	C_6_
1613 ± 24	1.2361 ± 0.0016	957 ± 24	1.230 ± 0.013	C_3/4α_
1613 ± 24	1.3004 ± 0.0011	957 ± 24	1.347 ± 0.004	C_3/4β_
2620 ± 40	1.2200 ± 0.0005	1154 ± 20	1.208 ± 0.008	C_3/4α_
2620 ± 40	1.4371 ± 0.0009	1154 ± 20	1.415 ± 0.002	C_3/4β_
1832 ± 22	1.0599 ± 0.0004	1042 ± 8	1.055 ± 0.003	C_2,α_
1832 ± 22	1.2255 ± 0.0034	1042 ± 8	1.252 ± 0.008	C_2,β_
6830 ± 90	2.0726 ± 0.0007	1736 ± 8	2.116 ± 0.001	C_1,α_
9950 ± 100	2.8231 ± 0.0011	2635 ± 13	2.835 ± 0.001	C_1,β_

a The chemical shifts correspond to measurement at 277 K.

b The chemical shifts correspond to measurement at 295 K.

Recording the spectra in the condensed range does not imply any principal problem as far as the dependence is concerned, eqn (3), used for extrapolation captures the physics correctly (see also Section 4.3). In practice, extrapolation to the temperature region distant from the measured points brings a large demand on the accuracy of these points. The accuracy of these measured points is expressed in a 95% confidence interval obtained from the fitting. Besides that, there is an unknown inaccuracy of the temperature setting in the measurement, even after careful temperature calibration. For the sample of pH = 7.00, we have not seen alarming signs in the data.

The parameters of the Eyring equation are presented with errors from the linearized fit in Table 2 and in Table S14 of the ESI,† with more decimal places. Confidence intervals are briefly discussed in Section 4.4.

Parameters of the Eyring equation for exchange rates obtained by linear fitting of the linearized Eying equation. Note that parameters for pH = 7.38 should be used only in low temperatures for estimates of exchange rates

**Table:** 

pH	6.21	Δ*G*^‡^ [J mol^−1^]
Site/parameter	*κ*	
C_6_	(1.37 ± 0.08) × 10^−6^	(2.227 ± 0.013) × 10^4^
C_3/4_	(1.54 ± 0.34) × 10^−6^	(2.38 ± 0.05) × 10^4^
C_3/4_	(2.5 ± 0.4) × 10^−5^	(3.054 ± 0.035) × 10^4^
C_2_	(8.2 ± 0.6) × 10^−6^	(2.793 ± 0.017) × 10^4^
C_1,α_	0.0162 ± 0.0007	(4.617 ± 0.010) × 10^4^
C_1,β_	0.00126 ± 0.00006	(3.852 ± 0.011) × 10^4^

**Table:** 

pH	7.00	Δ*G*^‡^
Site/parameter	*κ*	
C_6_	(1.43 ± 0.05) × 10^−5^	(2.810 ± 0.008) × 10^4^
C_3/4_	(6.0 ± 0.7) × 10^−5^	(3.192 ± 0.025) × 10^4^
C_3/4_	0.00228 ± 0.00027	(4.006 ± 0.027) × 10^4^
C_2_	0.000231 ± 0.000021	(3.508 ± 0.021) × 10^4^
C_1,α_	0.247 ± 0.024	(4.966 ± 0.022) × 10^4^
C_1,β_	0.071 ± 0.005	(4.549 ± 0.017) × 10^4^

**Table:** 

pH	7.38	Δ*G*^‡^
Site/parameter	*κ*	
C_6_	0.042 ± 0.005	(4.551 ± 0.028) × 10^4^
C_3/4_	(1.1 ± 0.6) × 10^3^	(6.83 ± 0.13) × 10^4^
C_3/4_	0.00080 ± 0.00023	(3.68 ± 0.07) × 10^4^
C_2_	(2.1 ± 0.8) × 10^3^	(7.00 ± 0.09) × 10^4^
C_1,α_	(5.7 ± 2.1) × 10^2^	(6.54 ± 0.08) × 10^4^
C_1,β_	2.9 ± 0.9	(5.23 ± 0.07) × 10^4^

The temperature dependence of the chemical shift with respect to the water line was not closely investigated. The chemical shifts are therefore reported (see the ESI,† Tables S7–S10) only at the measured temperature points. This should not be too limiting, since the temperature dependence is quite modest, considering its consequences on NMR/MRI experiments.

### 3.4 pH dependence

The pH-dependence of the exchange rate of glucose protons is of tremendous importance.^38–40^ In this study, we selected only three pH points. Our approach has been well suited for pH 6.21 and 7.00. With pH = 6.21, we were able to use temperature points up to 295 K, whereas for pH = 7.00, we carried out measurements condensed at low temperatures (see Section 3.3 for further information). Luckily, it was still possible to extrapolate all the way to 310 K with acceptable error estimates. In the case of pH = 7.38, we did not attempt to measure enough points to allow a similar extrapolation. The ultra-high magnetic field would probably allow for enough spectral resolution also for this sample (see the simulation in Fig. S4 in the ESI†). With available instrumentation, we do not recommend using the exchange rates extrapolated beyond the two measured points (see Tables S5 and S6 in the ESI†).

It is worth noting that different –OH groups have different pH dependences. For example, at 270 K (see Tables S1, S3 and S5 in the ESI†), the primary –OH group at C_6_ has a slower exchange rate at pH = 7.00 than at pH = 6.21 and at pH = 7.38 the exchange rate is again slightly higher. The secondary –OH groups at C_2_ to C_4_ exchange slower at lower pH. The hemiacetal –OH group at C_1_ is at the other edge of dependence, with the exchange rate increasing steeply upon pH increase. The difference in pH dependencies gives in principle the possibility to clarify more than one variable (such as glucose concentration and pH) at a time.

## 4 Discussion

In order to reliably assess the potential of NMR/MRI methods based on the chemical exchange of –OH protons of glucose, we started from careful preparation of glucose solution at three different pH points. We measured conventional one-dimensional proton spectra at a series of calibrated temperature points. We used rigorous simulation of the spectra using the extended Liouville space, where the exchange chemical shifts and exchange rates are further used in Monte Carlo simulation, in order to achieve good estimates of these parameters together with reliable error ranges. The temperature series of the exchange rates were further fitted to the Eyring equation, which also allowed reliable extrapolation to physiological temperatures for two lower pH points, together with propagation of the error ranges.

Knowing the chemical exchange parameters of glucose under the given chemical conditions, we can *e.g.* straightforwardly evaluate its contribution to the water *T*_2_ or *T*_1,ρ_. This further depends only on glucose concentration, and (a known) external magnetic field. This gives the possibility to measure the glucose concentration, which may be important in some cases. In other cases, it may be easy to measure the glucose concentration by different means, whereas the subject of interest may become pH or phosphate concentration. Even simultaneous measurement of two unknowns may be possible in principle. For assessing this possibility, reliably parameterized dependencies presented in this study may be crucial.

### 4.1 Dependence on buffering solution

It is important to restate, that the measurements were done in 1× PBS solution. This is often a good choice in order to mimic the physiological conditions, namely the pH and salt concentration. It has, however, an important implication in terms of proton-exchange rates, since the phosphate ion is known to catalyze the –OH proton exchange. The applicability to the physiological conditions has to be therefore assessed from this point of view. For example, the concentration of different inorganic phosphate species in blood is roughly ten times smaller (∼1 mM^41^) as compared to 1× PBS buffer. The intracellular concentration of phosphate varies, depending on the tissue. For example, values around 5 mM can be found for skeletal muscle,^42^ and an even higher concentration can be found under certain conditions.^43^ This makes 1× PBS an acceptable choice for many physiological situations. On the other hand, different buffers may be considered to approximate the physiological conditions of blood, with a lower concentration of phosphate and pH normally within 7.35–7.45.^30^

### 4.2 Assignment of chemical shift pairs

As presented in Section 3.2, the fitting should facilitate the assignment of which chemical shift of a given site at the α anomer belongs to the same site in the β anomer. Relying on the fitting procedure to facilitate the assignment through the identical exchange rate, may be too optimistic as the MCMC simulation has to overcome large barriers for possibly swapping the assignment and arrive at global minimum from a distant (in terms of parameters values) local minimum. At the same time, the “restricted” model may not be sufficiently complete. On the other hand, we have seen that the simulations converged to a consistently small sum of squares. Even still, the resulting assignment should be taken rather as a best guess and not an absolute truth.

### 4.3 Validity of the Eyring equation to determine the temperature dependence of the exchange rate

The Eyring equation has been derived for simple reactions. The chemical exchange does not fulfil this requirement. There is a forward reaction and a reverse reaction, each possibly with catalysis involved. Therefore, the parameters *κ* and Δ*G*^‡^ should be interpreted with great care. It is however expected that the dependence is still correct for the situation of largely different pools, such as when one pool consists of solvent protons. One pool is therefore always in large excess, and the flow is dependent on the reaction rate of the small pool. If we assume that the simple, forward reaction starts by dissociationGlu–OH → Glu–O^−^ + H^+^there is a several orders of magnitudes larger concentration of water molecules than other glucose molecules, therefore the dissociation would mostly result in the flow of H^+^ to the water pool, with the probability proportional to the (fractional) concentration of water *f*_H_2_O_ and therefore contribute to the chemical exchange relevant in this study. The other simple reaction, the dissociation of waterH–OH → H–O^−^ + H^+^results in the flow of H^+^ to glucose with a probability proportional to the concentration of glucose (*f*_glucose_), but dominantly, it would cause only exchange of protons between different water molecules, not contributing to chemical exchange in this study. For the exchange rate, the importance of dissociation at glucose would therefore contain the ratio 
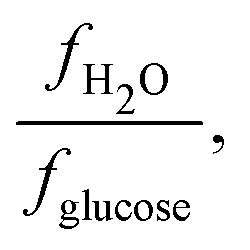
 which is large. Therefore, also the temperature dependence of the overall exchange rate should be well approximated by dependence of that reaction. In other situations, splitting the temperature dependence of the exchange rate into temperature dependencies of forward and reverse reactions may be more appropriate.

### 4.4 Confidence intervals of extrapolated exchange rates

Exchange rates and chemical shift values as obtained from MCMC simulations are represented as rigorous posterior distributions as long as the prior distribution is estimated correctly. On the other hand, propagation of the confidence intervals obtained from these distributions is partly obscured by the fact that the relationship of the temperature dependence is not exact. This can have different sources, such as slight simplification of the reaction model as described in Section 4.3, but possibly also due to increased proportion of dipole–dipole relaxation mechanism at the lowest temperatures. Due to these, together with random error, the parameters of the Eyring equation obtained using the six temperature points, can be obtained with irrelevantly wide confidence intervals, which would be propagated into wide confidence bands for the extrapolated curves. We therefore calculate the uncertainty of the Eyring equation parameters, only by propagation of the uncertainty of the exchange rates, and taking the Eyring equation parameters as if exactly matching the temperature dependence. This gives underestimated confidence bands, but in accord with the confidence intervals of the exchange rates obtained by MCMC, in a sense that the exchange rate and confidence interval recalculated from the fitted Eyring equation (Tables S1–S5 in the ESI†) would be close to a confidence interval obtained from MCMC for that temperature.

A more rigorous option would be to build a global model including the temperature dependence through the parameters of the Eyring equation. In this approach, the posterior distribution of the parameters would be directly obtained. Such a model would therefore contain two parameters determining the exchange rate in any temperature, only doubling the number of fitted variables as compared to fitting of a single temperature at a time, so 12 instead of 6 variables. At the same time, there is no similarly simple model for chemical shift values. If keeping the chemical shift values as fitted variables, this would multiply the number of variables by the number of temperatures, so 54 instead of 9. On the other hand, the chemical shift changes are very small especially for those other than the anomeric site at C_1_, therefore a linear correction can be considered, with two parameters for each chemical shift. The baseline correction can also be kept independent, resulting in 24 instead of 4 parameters, or, it may be justified to change the parameters into constants. Depending on the choice, the resulting model would contain 30, 66 or 90 parameters, requiring many more steps in order to sample the posterior distributions sufficiently. We did not attempt to follow this option in the current study, though it may be a straightforward extension of this study in the future.

### 4.5 Considering the direct OH–HO exchange

**Fig. 9:**
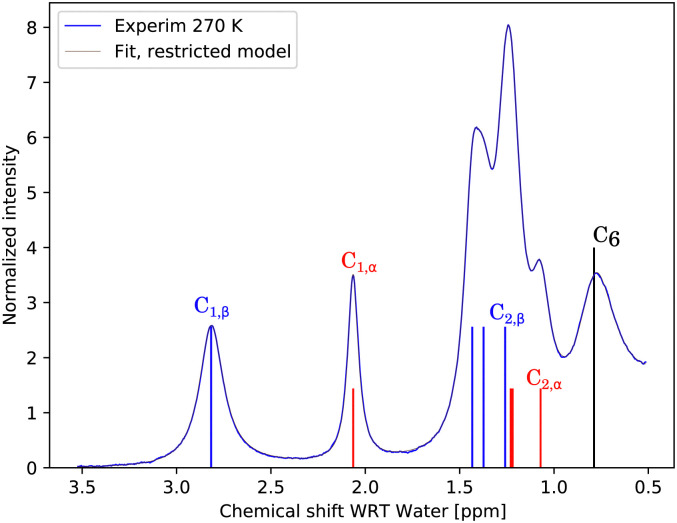
Comparison of the experimental spectrum for 270 K, 1 M glucose at pH = 6.21, and fit/simulation using the restricted model. The traces are nearly identical; therefore, the underlying experimental spectrum is visible only slightly. For C_1_–C_4_, with distinguishable chemical shifts for different anomers, the fitted chemical shifts are indicated by red and blue bars for α and β anomers, respectively, together with a likely assignment.

Due to the close proximity of the neighbouring –OH groups, it seems sensible to also consider direct exchange of protons between the –OH groups. We have introduced a model which allows for exchange between C_1_ and C_2_, C_2_ and C_3_, and also C_3_ and C_4_. The model is otherwise identical to the previous one, the “restricted model” (see Fig. 2, model “restricted with cross”). Noticing how accurate fit can be obtained using the restricted model (Fig. 9), it can be concluded that there is no space for additional degrees of freedom, and that the current data do not contain information regarding these additional parameters. A rigorous conclusion about the number of parameters in series of models, can be reached using the Bayesian approach of ref. 29. Such an approach is beyond the scope of this study. One can still conduct the MCMC simulation to obtain an extended space of parameters, a combination of restricted model with direct OH-HO exchange rates. With these, it would be possible to simulate NMR experiments, which could be possibly more sensitive in discriminating between different exchange routes. Such an experiment can be *e.g.* selective excitation of different –OH regions, conventional (continuous wave) CEST with a short saturation period, or pulsed CEST. These virtual experiments could then be used to carry out the actual measurements and evaluation. In this study, we did not take these thoughts further.

### 4.6 Concentration dependence

In this study, we use 1 M glucose solutions for all measurements and analysis. This enables direct observation of all the OH resonances in the conventional proton 1D proton spectrum and perform a robust analysis for a suitable temperature and pH range. The method, however, quickly loses its advantage when approaching physiological concentrations of glucose.

In this section, we exceptionally measure samples with lower concentrations of glucose, to estimate the possible bias in the exchange rate values due to the high concentration used throughout this study. We measured 1 M, 0.2 M and 0.04 M glucose in PBS buffer in pH = 6.21, at 280 K and 290 K.¶¶(The temperatures are obtaned from the instrument reading, without calibration, therefore the rates slightly differ from those reported in Tables S1, S2 in ESI.†) We used again only the conventional 1H spectra at 11.7 T spectrometer, this time with 2048 and 8192 scans for 0.2 M and 0.04 M samples, respectively. Using the same MCMC procedure to obtain the exchange rates, we were able to process well only the 0.2 M spectra, furthermore restricted only to the C_1_ resonances. The glucose OH region together with the fitted spectra are plotted in Fig. S10 in the ESI.† The 0.04 M spectra would require a different model for the baseline correction due to the *ca.* 2750 times stronger water signal nearby. Consequently, at 290 K, the fit quality for 0.04 M is poor compared to the other spectra. At 280 K the situation is more favourable, so we still include the results. The concentration dependence of the exchange rates is shown in Table 3.

**Table:** Exchange rate

Temperature, concentration	C_1,α_	C_1,β_
280 K, 1.0 M	230.2	474.9
280 K, 0.2 M	200.7	430.6
280 K, 0.04 M	195.1	416.7
290 K, 1.0 M	504.6	921.2
290 K, 0.2 M	356.4	650.4

The results show that the concentration dependence is itself also temperature dependent. For 280 K, the ratio *k*_ex,1M_/*k*_ex,0.2M_ is 230.2/200.7 = 1.14 and 474.9/430.6 = 1.10 for the α and β enantiomers of protons at C_1_, respectively. At 290 K, the ratios are already 1.42 for both α and β enantiomers. This has important consequences for our results, which are, in principle, correct only for the high concentration of glucose. Furthermore, we could not extract information on the concentration dependence of other exchangeable sites. On the other hand, both diluted and concentrated glucose in a pure phosphate buffer are far from the real composition of different physiological fluids. In any case, our results cover a limited subspace of conditions influencing the exchange rates. Interestingly, we see a surprising agreement between our results and the published results in Section 4.7.

In order to approach the physiological concentration of glucose, we would need to follow also the concentration dependence of the exchange rate as well as its temperature dependence. Only then could we extrapolate to physiological temperature and concentration. In order to extend the study into this dimension, a combination of our approach of conventional 1H spectra with CEST spectra and CPMG/*T*_1,ρ_ relaxation measurements would probably be required to cover the different regimes reliably.

The concentration dependence has also been observed in ref. 27 during a study of proton exchange at hydrated formaldehyde and its pH dependence in 0.01 M phosphate buffer. The authors reported 34 ± 18% faster exchange rate for 0.6 M as compared to 0.3 M solution. In Table 4 we also compare the exchange rates of formaldehyde in different pH and 1.1 °C with exchange rates at anomeric proton, which has similar chemical properties. We see closer similarity in the rates for pH 7.0 and 7.38, while for pH = 6.21, the differences are much larger.

**Table:** Exchange rates at 1.1 °C at the anomeric C_1_ site of glucose as compared to the exchange at hydrated formaldehyde

pH	6.21	7.0	7.38
C_1,α_	148.7	490.1	1130
C_1,β_	332.2	882.5	1772
Formaldehyde (ref. 27)	608	826	1242

The self-catalysis has been proposed for explanation of the concentration dependence of the exchange rate in the case of formaldehyde. The same would be consistent with our observations. In this respect, the first possibility is that the catalyst is the glucose molecule in Fig. 2, with all the OH groups in a neutral form. In order to become a catalyst, these OH groups would have to act as a stronger catalyst than the water OH groups.

The other option would be that the glucose becomes a catalyst only when it is itself in a process of proton exchange, and therefore ionized. In the short lifetime of the ionized form, in higher concentrations, there would be a higher chance to meet other molecules and catalyse their proton exchange.

The observation that the concentration dependence is itself strongly temperature dependent can be used to decide about the mechanism after detailed evaluation of competing dependencies, such as the molecular diffusion competing with the lifetime of the ionized form.

In both of these cases, the self catalysis reminds us also about the possible intramolecular proton exchange included in our models “with cross”, discussed in Section 4.5, since the nearby OH group at the same molecule can act as a catalyst for the other as if there was a local increase in concentration. In polysaccharides, the intramolecular proton exchange could scale up further. The current data do not contain enough evidence to make such predictions, as we were able to evaluate the concentration dependence of the exchange rate only for the chemically exceptional C_1_ protons, not present in common polysaccharides.

### 4.7 Comparison with a selection of published exchange models

A comparison of chemical exchange rates with the rates from different literature sources is complicated by all of the possible conditions affecting the rates. Disregarding the actual rates for the moment, there are large differences in the exchange models, namely in the number of exchanging sites resolved and in their assignment. The simplest models resolve only one effective chemical shift and one exchange rate. Their advantage is the simplicity that allows us to employ analytical formulas for experiments such as *T*_1,ρ_ or CPMG in contrast to numerical simulations such as in this study. We will return to one of these in Section 4.8. In Table 5 we compare our model with models used in important MRI studies concerning glucose.

Comparison of the exchange models of glucose at 310 K obtained in this study with published models

**Table:** 

Notes		Assignment					
		C_6_	C_3/4_	C_3/4_	C_2_	C_1,α_	C_1,β_
This work^a^	*δ* ppm	0.763 ± 0.003	1.230 ± 0.013, 1.347 ± 0.004	1.208 ± 0.008, 1.415 ± 0.002	1.055 ± 0.003, 1.252 ± 0.008	2.116 ± 0.001	2.835 ± 0.001
	*c*_frac_	1.0	0.36, 0.64	0.36, 0.64	0.36, 0.64	0.36	0.64
	pH = 6.21	1569 ± 10	957 ± 24	1154 ± 20	1042 ± 8	1736 ± 8	2635 ± 13
	pH = 7.00	1699 ± 8	1613 ± 24	2620 ± 40	1832 ± 22	6830 ± 90	9950 ± 100
b	pH = 7.38	5760 ± 100	21 600 ± 1600	3270 ± 120	22 100 ± 1200	34 900 ± 1700	28 200 ± 1100

a Values of the chemical shifts *δ* and fractional concentrations are for α and β enantiomer, respectively, where applicable. The fractional concentration, *c*_frac_ should be understood so that one distinct proton site has a value of 1.0. This is consistent with the cited literature.

b The values at pH = 7.38 should not be considered reliable for 310 K.

c Model 1 has many features of our model, so keeping in mind the 0.2 difference in pH, it can be compared with our model at pH = 7.00, the largest difference is for sites C_2,3,4_, where we note *ca.* 3–5 times faster exchange rate compared to our model.

d Model 2 does not assign the resonances correctly, and also overlooks the α,β enantiomeric ratio. This is especially important for the resonances at 2.0 and 2.9 ppm, as these are well resolved, most significant in terms of the possibility to irradiate them separately for example in the CEST experiment. They are also the most effective in causing R_2_ type of relaxation. It is therefore important to use the correct fractional concentrations that are *ca.* 0.36 and 0.64, and not 1.0 and 1.0. The exchange rates are not directly comparable, for which one reason is the five-times less concentrated phosphate buffer solution.

e Model 3, similarly to Model 2, disregards the α,β enantiomeric ratio, which is important for the resonance at C_1_, here at 2.2 and 2.8 ppm. Besides the pH difference, the buffer composition is the same as in our study, therefore the exchange rates are also comparable.

**Table:** 

Models 1 (ref. 21)^c^		C_6_	C_2,3,4_	C_1,α_	C_1,β_
	*δ* ppm	0.66	1.28	2.08	2.88
	*c*_frac_	1.0	3.0	0.36	0.64
	pH = 7.2	1570 ± 220	7360 ± 476	5662 ± 2660	10 000 ± 3800
Multi-pH fit	pH = 7.2	2900 ± 500	6500 ± 170	5200 ± 500	14 000 ± 650

**Table:** 

Model 2 (ref. 17 and 18)^d^		C_6_	C_3_	C_1,2,4_
0.2× PBS	*δ* ppm	2.9	2.0	1.3
	*c*_frac_	1.0	1.0	3.0
	7.2	1275	1700	935

**Table:** 

Model 3 (ref. 44)^e^				
	*δ* ppm	1.2	2.2	2.8
	*c*_frac_	3.0	1.0	1.0
	7.2	4000	8000	10 000

We see perhaps surprising agreement for exchange rates at the anomeric sites, when comparing our results at pH = 7.00 and the results of Model 1 and Model 3 at pH = 7.2. There seems to be a nearly identical effect of high glucose concentration and increased pH value by 0.2 units in diluted samples of glucose in PBS.

### 4.8 Glucose as a relaxation agent

The OH resonances of glucose may be used in CEST experiments, where each OH with distinct chemical shifts is irradiated separately. On the other hand, the exchangeable OH protons can create a standard *T*_2_ contrast. This has been studied in detail by Yadav *et al.*^45^ There, the *R*_2_ relaxation rates were measured in a CPMG experiment at 22 °C at a series of pH values in PBS buffer and are therefore compatible with our results. For the physiological pH of 7.3, the authors fitted an average chemical shift and exchange rate using an analytical formula for *R*_2,ex_, for a two-site exchange model,^46^ obtaining *δ* = 1.44 ppm and *k*_ex_ = 2200 Hz. Similarly to our approach, they extrapolated the exchange rate to 37 °C using the Arrhenius equation, giving *k*_ex,310K_ = 4600 Hz. The *R*_2_ relaxation rates were converted to relaxivity (s^−1^ mM^−1^). We use our results to simulate the CPMG measurement, with 10 echo periods of 20 ms and realistic 90° and 180° RF pulses. The relaxivity results are compared in Table 6.

Relaxivity

**Table:** 

pH 7.3		295.15 K (measurement)	310.15 K (extrapol.)
Ref. 45	3.0 T	0.021	0.012
	7.0 T	0.060	0.053
	11.7 T	0.077	0.102

pH 6.21	3.0 T	0.021	0.024
	7.0 T	0.030	0.047
	11.7 T	0.032	0.056

pH 7.00	3.0 T	0.024	0.017
	7.0 T	0.048	0.055
	11.7 T	0.058	0.089

pH 7.38	3.0 T	0.013	0.0055
	7.0 T	0.048	0.025
	11.7 T	0.091	0.056

a Simulated by our program to verify the equality of the approaches.

**Table:** 

Simulated^a^	Parameters from ref. 45:	1.44 ppm, *k*_ex_ = 4600 Hz	310.15 K
	3.0 T		0.012
	7.0 T		0.053
	11.7 T		0.10

**Fig. 10:**
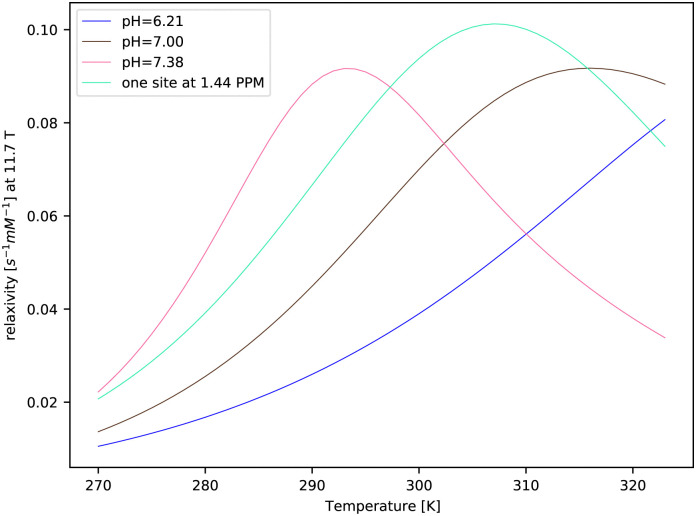
Simulated relaxivity at 11.7 T using our exchange models of glucose and compared to a one (and one) site model, nearly identical to that of ref. 45.

We can see that the relaxivity obtained experimentally by Yadav *et al.* at 22 °C for different magnetic fields is comparable to our results. For the physiological temperature, none of our conditions reach the predicted relaxivity of 0.102 s^−1^ mM^−1^. This high relaxivity at 37 °C, predicted by their two-site exchange model is near its maximum in its temperature dependence for 11.7 T. A more realistic model, such as in the current study, where there are more sites with different offsets and exchange rates, would generally predict a wider, and therefore lower, maximum of the relaxivity. We have simulated relaxivity as a function of temperature using our results in Fig. 10.

The results are compared with the relaxivity at 11.7 T obtained by a two-site model, where the offset is at 1.44 ppm and the temperature dependence of *k*_ex_ is close to that reported by Yadav *et al.* The expected effect of a wider maximum of exchange rates for a model with more sites is not too significant in this case.

### 4.9 Using the exchange parameters in virtual experiments

It is straightforward to simulate the exchange contribution to the water *T*_2_ or *T*_1,ρ_, adiabatic *T*_1,ρ_ ref. 47 relaxation times or other experiments and possibly compare the contribution to other mechanisms such as common dipole–dipole relaxation. First, we conducted high-resolution NMR of the glucose solution, introducing a standard CPMG pulse train before the acquisition.

**Fig. 11:**
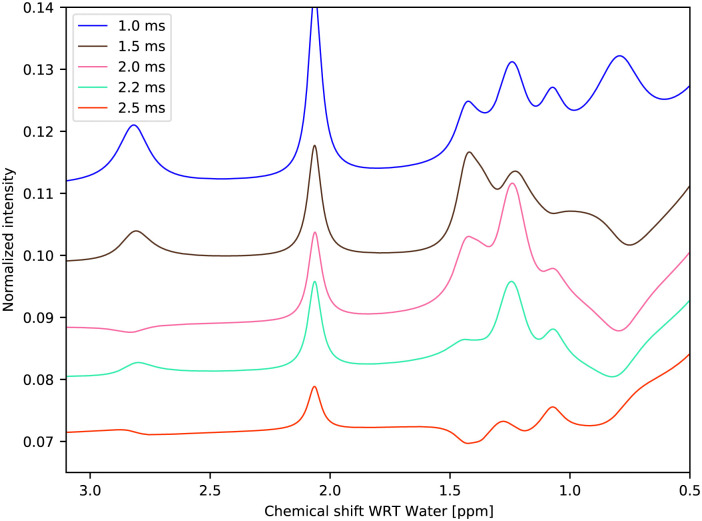
Simulated CPMG experiment using fitted exchange parameters of 1 M glucose at 270.0 K and pH = 6.21. Different traces correspond to different half-echo times, known in Bruker spectrometers as *d*20, all of them after four echo inversions.

As can be seen in Fig. 11, with a half-echo time of 1.5 ms and longer, one obtains a spectrum with a rich phase pattern. It is clear that such spectra can be used to verify or refine the exchange rates.

Simulation of the CEST spectrum would be equally straightforward, and the results may be compared with many CEST-based studies. In the CPMG simulation, used in section 4.8, instead of simulating the FID, detecting only the whole magnetization on the quadrature detector is facilitated by ‖*J*^†^_−_·*ρ*‖, where *J*_−_ is the Liouville state defined by the lowering operator and *ρ* is the density matrix. This would also be the case for imaging. We can promptly obtain the exchange contribution to *T*_2_.

## 5 Conclusions

It may be argued that glucose is the most important molecule in the living world. In that sense, it may be unexpected, that there are still properties that are not well known. We have obtained temperature dependence of exchange parameters of all distinguishable glucose –OH protons, with reliable confidence intervals in three different pH values, with two of them reaching physiological temperature.

We show a simple example of how the parameters can be used in virtual experiments, helping to develop new applications for glucose as an NMR/MRI probe. Knowing the chemical exchange parameters of glucose in the given chemical conditions, we can for example straightforwardly evaluate its contribution to the water *T*_2_ or *T*_1,ρ_. The exchange parameters give opportunities to determine either glucose concentration or pH, when the other parameter is known, or in favourable cases, also both simultaneously.

For future studies, it would be valuable to determine the pH dependence of exchange parameters using more pH points. The next additional dimension of the model, can be obtained by adding the dependence on phosphate ion concentration. This would result in a rather complete model of the exchange properties of glucose hydroxyls under any physiological conditions.

## Author contributions

J. M.: investigation, conceptualization, software, methodology, validation, and writing original draft. J. K.: conceptualization, thorough review and editing. P. H.: methodology and editing. S. M.: conceptualization, writing and editing the manuscript T. L.: conceptualization and editing.

## Conflicts of interest

There are no conflicts to declare.

## Supplementary Material

CP-025-D3CP01973J-s001
