# Supplementary material for: Glucose exchange parameters in a subset of physiological conditions
Source: Phys Chem Chem Phys. 2023 Aug 1;25(34):22965–78. doi: 10.1039/d3cp01973j (PMC10467565; doi:10.1039/d3cp01973j)
Supplement: CP-025-D3CP01973J-s001 [file CP-025-D3CP01973J-s001.pdf]

Electronic Supplementary Material (ESI) for Physical Chemistry Chemical Physics.
This journal is © the Owner Societies 2023

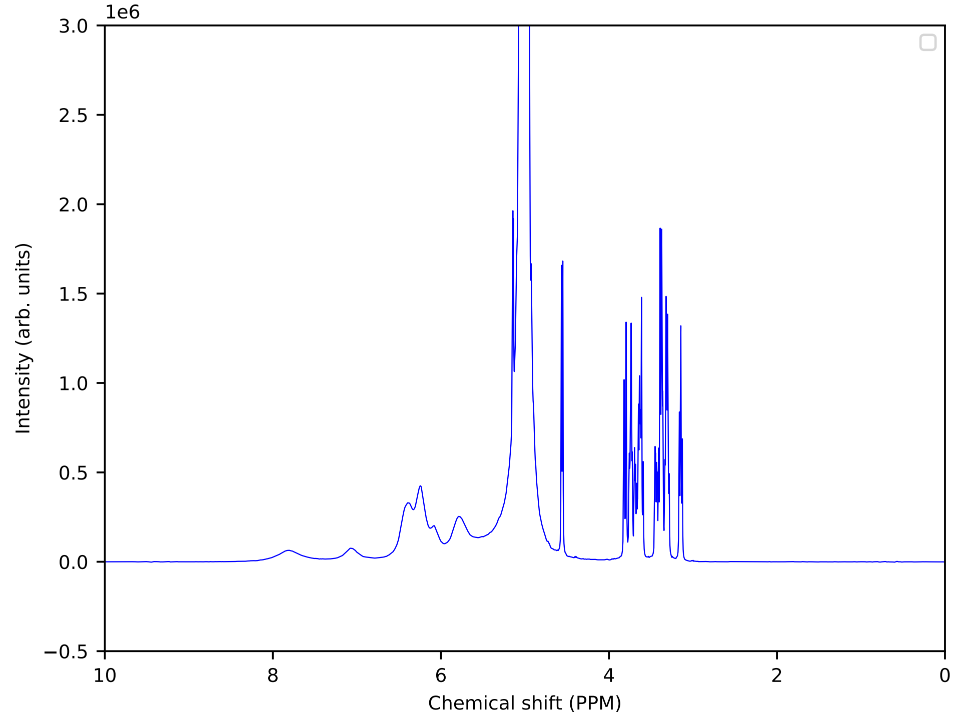

Figure 1: Full proton experimental spectrum of 1M glucose in 1xPBS, pH=7.00, temperature = 270 K at magnetic field of 11.7 T

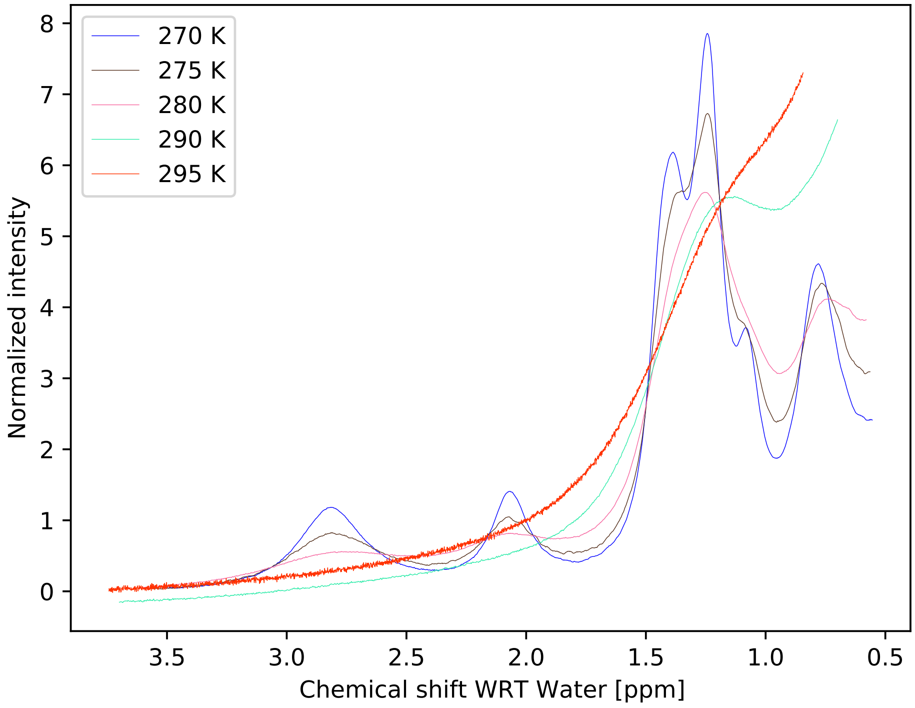

Figure 2: Set of experimental spectra in -OH region of 1M glucose in 1xPBS, pH=7.00, at magnetic field of 11.7 T. At this pH, the spectra loose quickly resolution with increasing temperature.

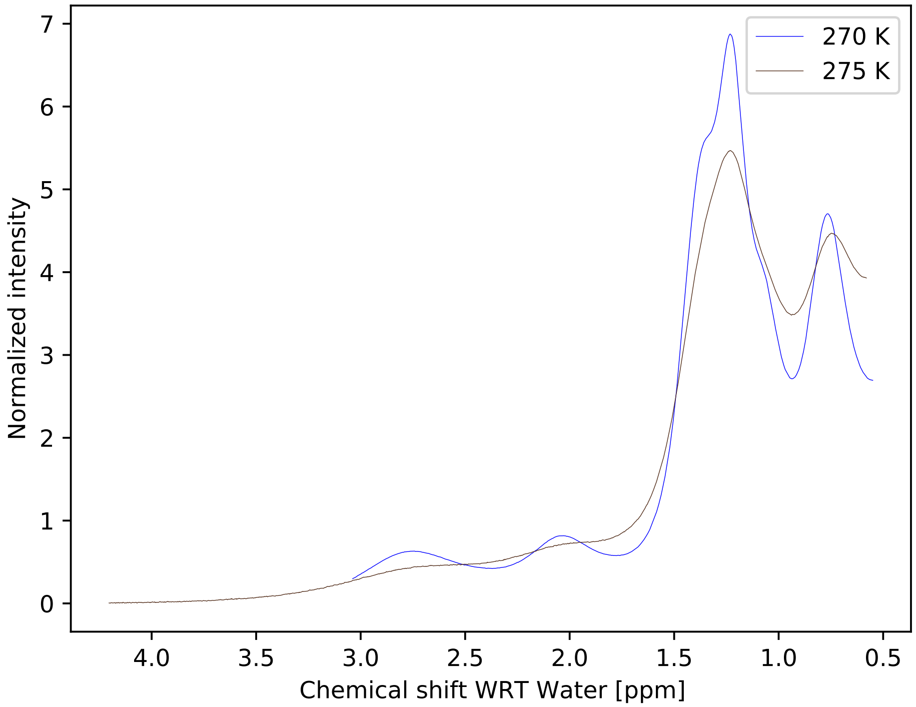

Figure 3: Experimental spectra in -OH region of 1M glucose in 1xPBS, pH=7.38, at magnetic field of 11.7 T. In this pH, already at T=275 K, the resolution is very poor.

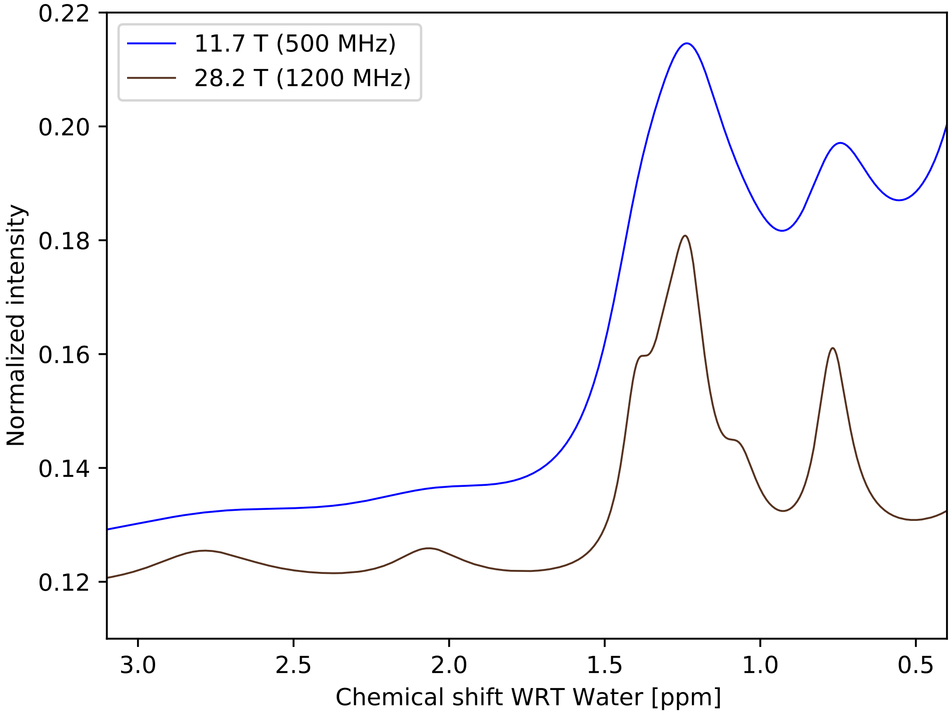

Figure 4: Simulated spectra in -OH region of 1M glucose in 1xPBS, at 275 K (calibrated temperature $\cong 273.5\,\mathrm{K}$), pH=7.38, at magnetic fields of 11.7 T and 28.2 T. The ultra high field gives good separation of glucose OH signals as well as their separation from the water signal.

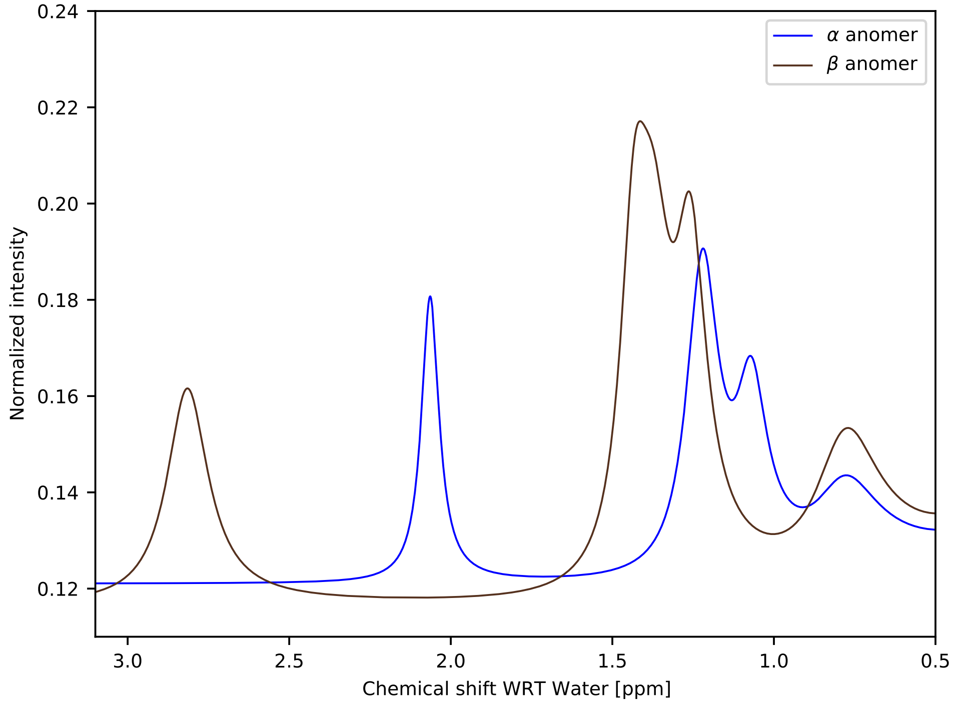

Figure 5: Hypothetical simulated spectra in -OH region of separate α and β anomers of 1M glucose in 1xPBS, at pH=6.21, temperature = 270 K at magnetic field of 11.7 T

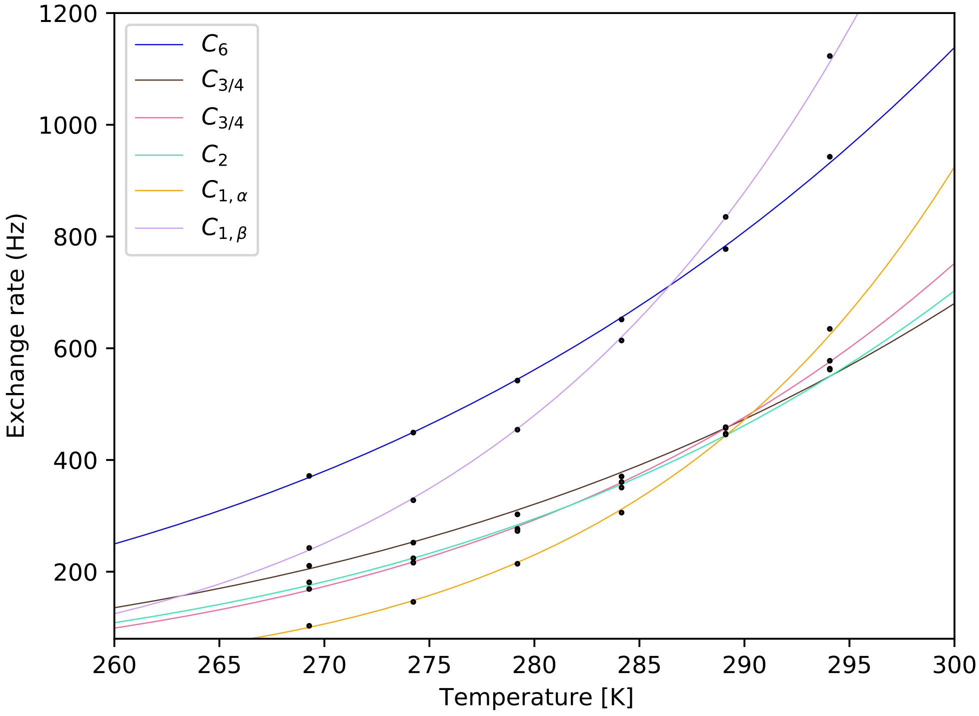

Figure 6: Fits of the temperature series for sample of pH=6.21. Exchange rate obtained by MCMC fit for each site are shown at calibrated temperatures, zoomed around the data points.

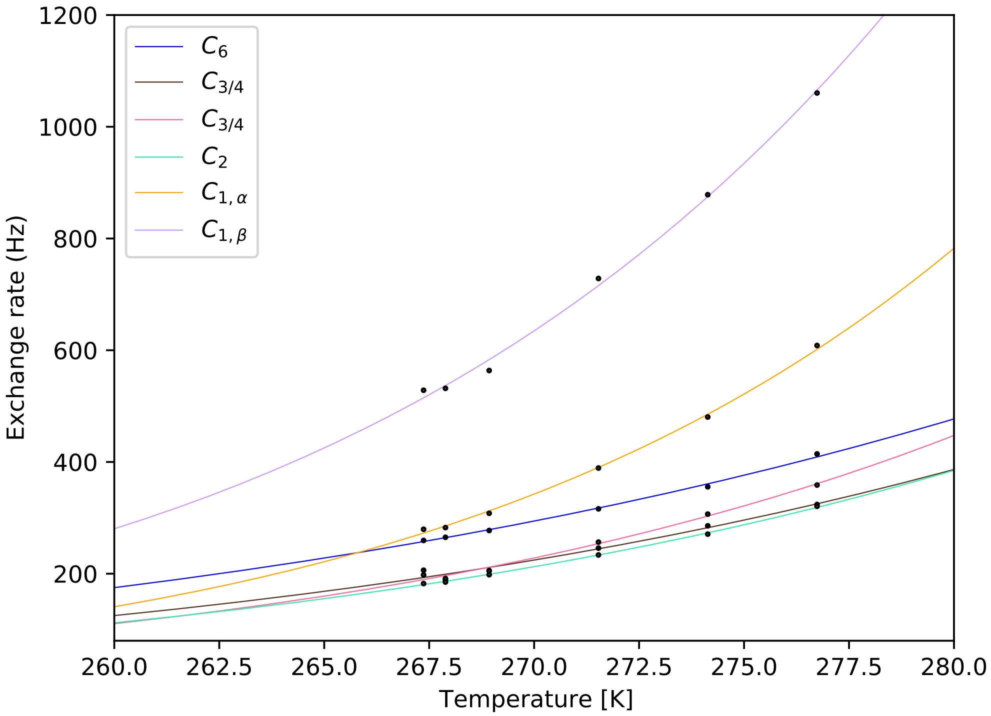

Figure 7: Fits of the temperature series for sample of pH=7.00. Exchange rate obtained by MCMC fit for each site are shown at calibrated temperatures, zoomed around the data points. Note that the lowest-temperature points are systematically out of trend. Removing these points may be considered after rigorous justification.

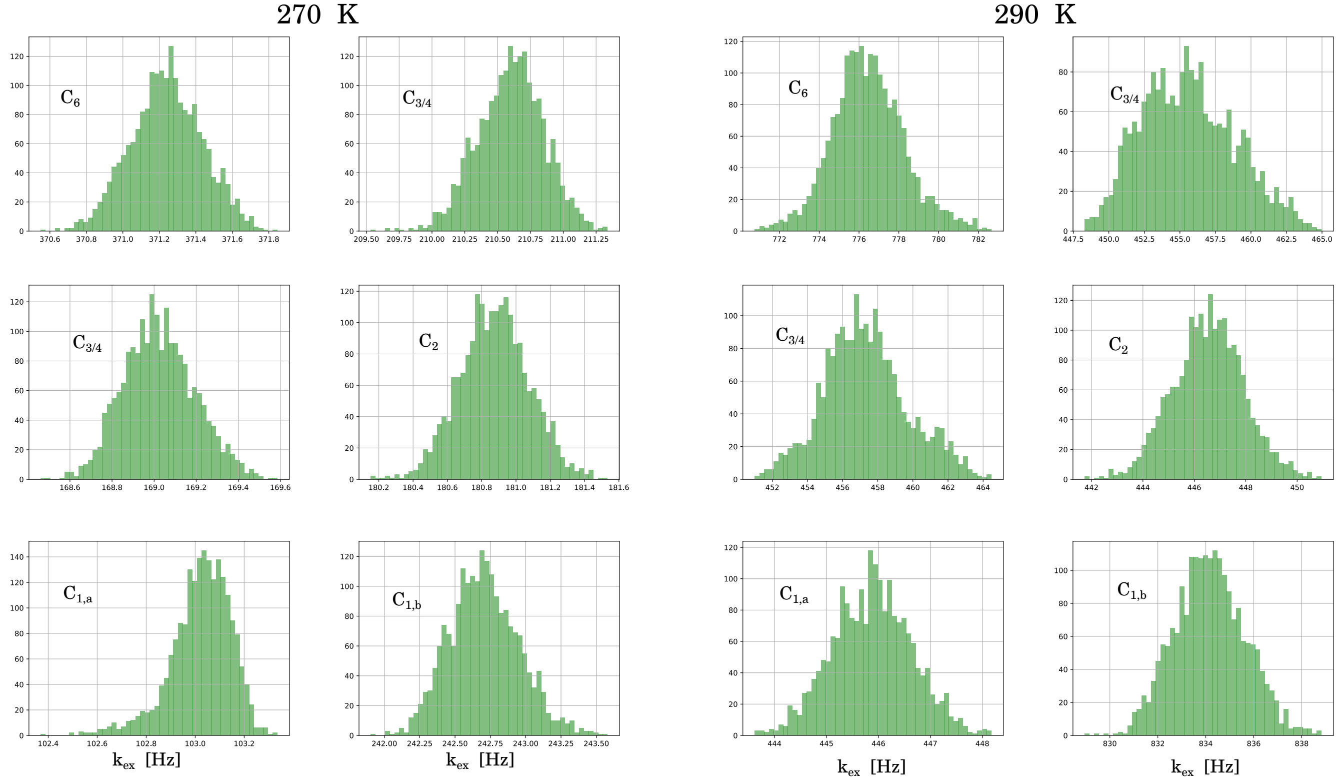

Figure 8: Examples of histograms of exchange rates obtained in MCMC simulations, for sample of pH=6.21, at 270 K and 290 K. The distributions are approximately symmetric.

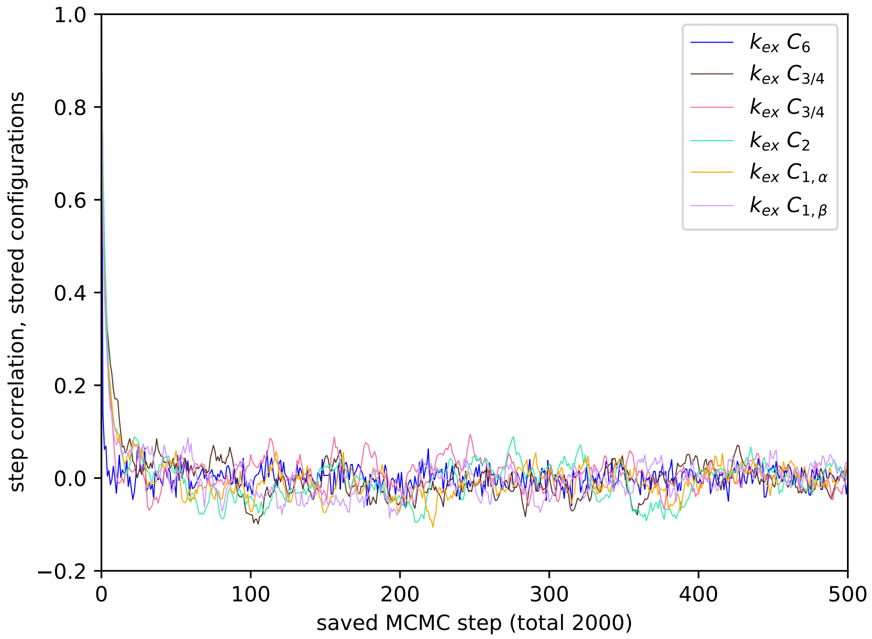

Figure 9: Examples correlation functions of saved steps obtained in MCMC simulations, for sample of pH=6.21, at 270 K. One can see that the correlation time is short on the scale of MCMC simulation, therefore the space is probably sampled well.

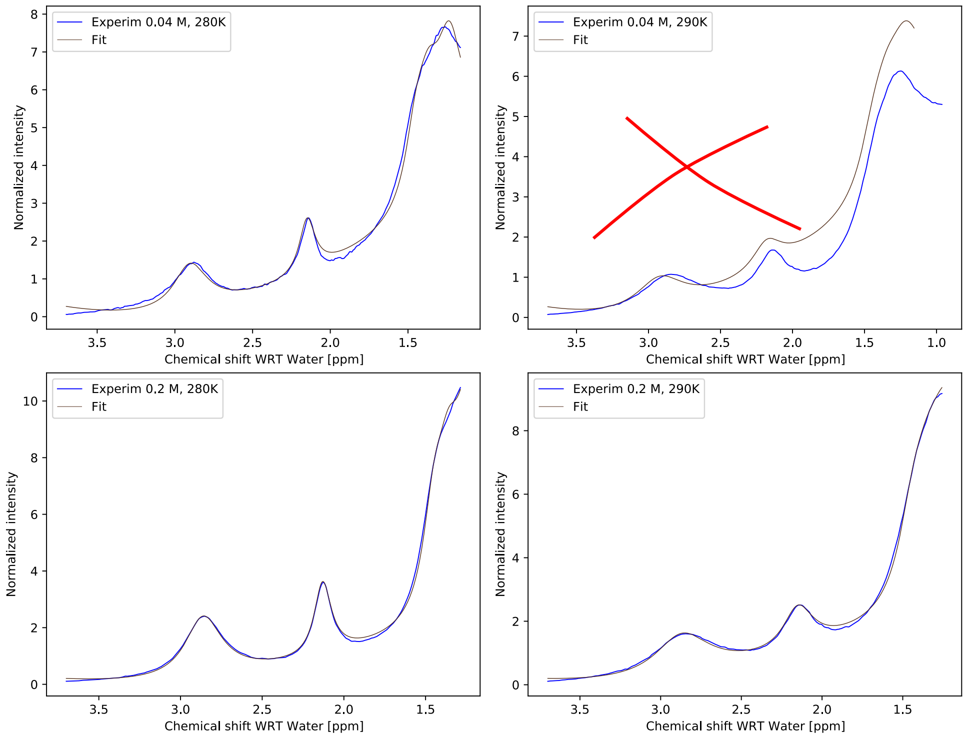

Figure 10: Spectra at low concentration of glucose, pH = 6.21, clearly the fitting for spectrum of 0.04 M glucose at 290 K has to be discarded.

Table 1: Exchange rates extrapolated to 310 K, 1M glucose at pH=6.21, using restricted model, with partial assignment.

T(K)	C_6	$C_{3/4}$	$C_{3/4}$	C_2	$C_{1,\alpha}$	$C_{1,\beta}$
260.0	249.7 ± 0.8	135.5 ± 1.8	99.1 ± 0.9	108.8 ± 0.5	46.46 ± 0.17	124.8 ± 0.4
260.25	252.5 ± 0.8	137.1 ± 1.8	100.5 ± 0.9	110.2 ± 0.5	47.47 ± 0.17	127.1 ± 0.4
260.5	255.2 ± 0.8	138.7 ± 1.8	102.0 ± 0.9	111.7 ± 0.5	48.50 ± 0.17	129.4 ± 0.4
260.75	258.0 ± 0.8	140.3 ± 1.8	103.5 ± 0.9	113.2 ± 0.5	49.55 ± 0.17	131.7 ± 0.4
261.0	260.8 ± 0.8	141.9 ± 1.8	105.0 ± 0.9	114.7 ± 0.5	50.62 ± 0.18	134.1 ± 0.4
261.25	263.6 ± 0.8	143.5 ± 1.7	106.5 ± 0.9	116.3 ± 0.5	51.71 ± 0.18	136.6 ± 0.4
261.5	266.5 ± 0.8	145.2 ± 1.7	108.1 ± 0.9	117.8 ± 0.5	52.82 ± 0.18	139.0 ± 0.4
261.75	269.3 ± 0.8	146.8 ± 1.7	109.6 ± 0.9	119.4 ± 0.5	53.95 ± 0.18	141.5 ± 0.4
262.0	272.2 ± 0.8	148.5 ± 1.7	111.2 ± 0.9	121.0 ± 0.5	55.11 ± 0.18	144.1 ± 0.4
262.25	275.2 ± 0.8	150.2 ± 1.7	112.8 ± 0.9	122.6 ± 0.5	56.29 ± 0.18	146.7 ± 0.4
262.5	278.1 ± 0.8	151.9 ± 1.7	114.5 ± 0.9	124.2 ± 0.5	57.49 ± 0.18	149.3 ± 0.4
262.75	281.1 ± 0.8	153.7 ± 1.7	116.1 ± 0.9	125.8 ± 0.5	58.71 ± 0.19	152.0 ± 0.4
263.0	284.1 ± 0.8	155.4 ± 1.7	117.8 ± 0.9	127.5 ± 0.5	59.96 ± 0.19	154.7 ± 0.4
263.25	287.1 ± 0.8	157.2 ± 1.6	119.4 ± 0.9	129.2 ± 0.5	61.24 ± 0.19	157.4 ± 0.4
263.5	290.2 ± 0.8	159.0 ± 1.6	121.2 ± 0.9	130.9 ± 0.5	62.53 ± 0.19	160.2 ± 0.4
263.75	293.3 ± 0.7	160.8 ± 1.6	122.9 ± 0.9	132.6 ± 0.5	63.85 ± 0.19	163.1 ± 0.4
264.0	296.4 ± 0.7	162.6 ± 1.6	124.6 ± 0.8	134.3 ± 0.5	65.20 ± 0.19	166.0 ± 0.4
264.25	299.6 ± 0.7	164.4 ± 1.6	126.4 ± 0.8	136.1 ± 0.5	66.58 ± 0.19	168.9 ± 0.4
264.5	302.7 ± 0.7	166.3 ± 1.6	128.2 ± 0.8	137.9 ± 0.5	67.98 ± 0.20	171.9 ± 0.4
264.75	305.9 ± 0.7	168.1 ± 1.5	130.0 ± 0.8	139.7 ± 0.5	69.40 ± 0.20	174.9 ± 0.4
265.0	309.1 ± 0.7	170.0 ± 1.5	131.8 ± 0.8	141.5 ± 0.5	70.86 ± 0.20	178.0 ± 0.4
265.25	312.4 ± 0.7	171.9 ± 1.5	133.7 ± 0.8	143.3 ± 0.5	72.34 ± 0.20	181.1 ± 0.4
265.5	315.7 ± 0.7	173.9 ± 1.5	135.6 ± 0.8	145.2 ± 0.5	73.85 ± 0.20	184.3 ± 0.4
265.75	319.0 ± 0.7	175.8 ± 1.5	137.5 ± 0.8	147.0 ± 0.5	75.39 ± 0.20	187.6 ± 0.4
266.0	322.3 ± 0.7	177.8 ± 1.4	139.4 ± 0.8	148.9 ± 0.5	76.95 ± 0.20	190.8 ± 0.4
266.25	325.7 ± 0.7	179.7 ± 1.4	141.4 ± 0.8	150.9 ± 0.5	78.55 ± 0.21	194.2 ± 0.4
266.5	329.1 ± 0.6	181.7 ± 1.4	143.4 ± 0.8	152.8 ± 0.5	80.18 ± 0.21	197.5 ± 0.4
266.75	332.5 ± 0.6	183.8 ± 1.4	145.4 ± 0.8	154.8 ± 0.5	81.84 ± 0.21	201.0 ± 0.4
267.0	336.0 ± 0.6	185.8 ± 1.4	147.4 ± 0.7	156.7 ± 0.5	83.53 ± 0.21	204.5 ± 0.4
267.25	339.5 ± 0.6	187.8 ± 1.3	149.4 ± 0.7	158.7 ± 0.5	85.25 ± 0.21	208.0 ± 0.4
267.5	343.0 ± 0.6	189.9 ± 1.3	151.5 ± 0.7	160.8 ± 0.5	87.00 ± 0.21	211.6 ± 0.4
267.75	346.5 ± 0.6	192.0 ± 1.3	153.6 ± 0.7	162.8 ± 0.5	88.78 ± 0.21	215.2 ± 0.4
268.0	350.1 ± 0.6	194.1 ± 1.3	155.7 ± 0.7	164.9 ± 0.5	90.60 ± 0.21	219.0 ± 0.4
268.25	353.7 ± 0.6	196.2 ± 1.2	157.9 ± 0.7	167.0 ± 0.5	92.46 ± 0.21	222.7 ± 0.4
268.5	357.4 ± 0.6	198.4 ± 1.2	160.0 ± 0.7	169.1 ± 0.5	94.34 ± 0.21	226.5 ± 0.4
268.75	361.0 ± 0.6	200.5 ± 1.2	162.2 ± 0.7	171.2 ± 0.4	96.27 ± 0.22	230.4 ± 0.4
269.0	364.7 ± 0.5	202.7 ± 1.2	164.5 ± 0.7	173.4 ± 0.4	98.22 ± 0.22	234.4 ± 0.4
269.25	368.4 ± 0.5	204.9 ± 1.1	166.7 ± 0.6	175.6 ± 0.4	100.22 ± 0.22	238.4 ± 0.4
269.5	372.2 ± 0.5	207.2 ± 1.1	169.0 ± 0.6	177.8 ± 0.4	102.25 ± 0.22	242.4 ± 0.4
269.75	376.0 ± 0.5	209.4 ± 1.1	171.3 ± 0.6	180.0 ± 0.4	104.32 ± 0.22	246.5 ± 0.4
270.0	379.8 ± 0.5	211.7 ± 1.0	173.6 ± 0.6	182.3 ± 0.4	106.42 ± 0.22	250.7 ± 0.4
270.25	383.7 ± 0.5	214.0 ± 1.0	176.0 ± 0.6	184.6 ± 0.4	108.57 ± 0.22	255.0 ± 0.4
270.5	387.6 ± 0.5	216.3 ± 1.0	178.4 ± 0.6	186.9 ± 0.4	110.75 ± 0.22	259.3 ± 0.4
270.75	391.5 ± 0.5	218.6 ± 1.0	180.8 ± 0.6	189.2 ± 0.4	112.98 ± 0.22	263.7 ± 0.4
271.0	395.5 ± 0.4	221.0 ± 0.9	183.3 ± 0.5	191.6 ± 0.4	115.24 ± 0.22	268.1 ± 0.4
271.25	399.4 ± 0.4	223.3 ± 0.9	185.7 ± 0.5	193.9 ± 0.4	117.55 ± 0.22	272.6 ± 0.4
271.5	403.5 ± 0.4	225.7 ± 0.9	188.2 ± 0.5	196.3 ± 0.4	119.89 ± 0.22	277.2 ± 0.4
271.75	407.5 ± 0.4	228.1 ± 0.8	190.8 ± 0.5	198.8 ± 0.4	122.28 ± 0.23	281.8 ± 0.4
272.0	411.6 ± 0.4	230.6 ± 0.8	193.3 ± 0.5	201.2 ± 0.4	124.72 ± 0.23	286.5 ± 0.4
272.25	415.7 ± 0.4	233.0 ± 0.8	195.9 ± 0.5	203.7 ± 0.4	127.19 ± 0.23	291.3 ± 0.4
272.5	419.9 ± 0.4	235.5 ± 0.7	198.6 ± 0.5	206.2 ± 0.4	129.71 ± 0.23	296.2 ± 0.4
272.75	424.1 ± 0.4	238.0 ± 0.7	201.2 ± 0.4	208.8 ± 0.4	132.28 ± 0.23	301.1 ± 0.4
273.0	428.3 ± 0.4	240.5 ± 0.7	203.9 ± 0.4	211.3 ± 0.4	134.89 ± 0.23	306.1 ± 0.4
273.25	432.5 ± 0.4	243.1 ± 0.7	206.6 ± 0.4	213.9 ± 0.4	137.55 ± 0.23	311.2 ± 0.4
273.5	436.84 ± 0.35	245.6 ± 0.6	209.3 ± 0.4	216.5 ± 0.4	140.26 ± 0.23	316.3 ± 0.4
273.75	441.17 ± 0.34	248.2 ± 0.6	212.1 ± 0.4	219.2 ± 0.4	143.02 ± 0.23	321.6 ± 0.4
274.0	445.53 ± 0.34	250.8 ± 0.6	214.9 ± 0.4	221.8 ± 0.4	145.82 ± 0.23	326.9 ± 0.4
274.25	449.93 ± 0.33	253.5 ± 0.6	217.8 ± 0.4	224.5 ± 0.4	148.68 ± 0.23	332.2 ± 0.4
274.5	454.36 ± 0.33	256.1 ± 0.6	220.6 ± 0.4	227.3 ± 0.4	151.58 ± 0.23	337.7 ± 0.4
274.75	458.83 ± 0.33	258.8 ± 0.6	223.6 ± 0.4	230.0 ± 0.4	154.54 ± 0.23	343.2 ± 0.4
275.0	463.34 ± 0.33	261.5 ± 0.6	226.5 ± 0.4	232.8 ± 0.4	157.55 ± 0.24	348.9 ± 0.4
275.25	467.88 ± 0.33	264.2 ± 0.6	229.5 ± 0.4	235.6 ± 0.4	160.61 ± 0.24	354.6 ± 0.4
275.5	472.46 ± 0.34	267.0 ± 0.6	232.5 ± 0.4	238.4 ± 0.4	163.73 ± 0.24	360.3 ± 0.4
275.75	477.08 ± 0.34	269.8 ± 0.6	235.5 ± 0.4	241.3 ± 0.4	166.90 ± 0.24	366.2 ± 0.4
276.0	481.73 ± 0.35	272.6 ± 0.6	238.6 ± 0.4	244.2 ± 0.4	170.12 ± 0.24	372.2 ± 0.4
276.25	486.4 ± 0.4	275.4 ± 0.6	241.7 ± 0.4	247.1 ± 0.4	173.41 ± 0.24	378.2 ± 0.4
276.5	491.1 ± 0.4	278.2 ± 0.6	244.8 ± 0.4	250.1 ± 0.4	176.75 ± 0.24	384.3 ± 0.4
276.75	495.9 ± 0.4	281.1 ± 0.7	248.0 ± 0.4	253.1 ± 0.4	180.15 ± 0.25	390.5 ± 0.4
277.0	500.7 ± 0.4	284.0 ± 0.7	251.2 ± 0.5	256.1 ± 0.4	183.61 ± 0.25	396.8 ± 0.4
277.25	505.6 ± 0.4	286.9 ± 0.7	254.5 ± 0.5	259.2 ± 0.4	187.12 ± 0.25	403.2 ± 0.4
277.5	510.4 ± 0.4	289.9 ± 0.8	257.8 ± 0.5	262.2 ± 0.4	190.70 ± 0.25	409.7 ± 0.4
277.75	515.4 ± 0.4	292.9 ± 0.8	261.1 ± 0.5	265.4 ± 0.4	194.34 ± 0.25	416.3 ± 0.4
278.0	520.3 ± 0.5	295.9 ± 0.9	264.5 ± 0.6	268.5 ± 0.4	198.05 ± 0.26	423.0 ± 0.4
278.25	525.3 ± 0.5	298.9 ± 0.9	267.9 ± 0.6	271.7 ± 0.4	201.82 ± 0.26	429.7 ± 0.4
278.5	530.3 ± 0.5	301.9 ± 1.0	271.3 ± 0.6	274.9 ± 0.4	205.65 ± 0.26	436.6 ± 0.4
278.75	535.4 ± 0.5	305.0 ± 1.0	274.8 ± 0.7	278.1 ± 0.5	209.55 ± 0.27	443.6 ± 0.5
279.0	540.5 ± 0.6	308.1 ± 1.1	278.3 ± 0.7	281.4 ± 0.5	213.51 ± 0.27	450.6 ± 0.5
279.25	545.7 ± 0.6	311.2 ± 1.1	281.8 ± 0.7	284.7 ± 0.5	217.55 ± 0.28	457.8 ± 0.5
279.5	550.9 ± 0.6	314.4 ± 1.2	285.4 ± 0.8	288.0 ± 0.5	221.65 ± 0.28	465.0 ± 0.5
279.75	556.1 ± 0.6	317.6 ± 1.3	289.1 ± 0.8	291.4 ± 0.5	225.82 ± 0.28	472.4 ± 0.5
280.0	561.4 ± 0.7	320.8 ± 1.3	292.7 ± 0.9	294.8 ± 0.5	230.06 ± 0.29	479.9 ± 0.5
280.25	566.7 ± 0.7	324.0 ± 1.4	296.4 ± 0.9	298.2 ± 0.5	234.38 ± 0.30	487.4 ± 0.5
280.5	572.1 ± 0.7	327.3 ± 1.5	300.2 ± 1.0	301.7 ± 0.6	238.77 ± 0.30	495.1 ± 0.6
280.75	577.5 ± 0.7	330.5 ± 1.5	304.0 ± 1.0	305.2 ± 0.6	243.23 ± 0.31	502.9 ± 0.6
281.0	582.9 ± 0.8	333.9 ± 1.6	307.8 ± 1.1	308.8 ± 0.6	247.77 ± 0.31	510.8 ± 0.6
281.25	588.4 ± 0.8	337.2 ± 1.7	311.7 ± 1.1	312.3 ± 0.6	252.39 ± 0.32	518.8 ± 0.6
281.5	593.9 ± 0.8	340.6 ± 1.8	315.6 ± 1.2	316.0 ± 0.6	257.08 ± 0.33	526.9 ± 0.6
281.75	599.5 ± 0.9	344.0 ± 1.9	319.6 ± 1.2	319.6 ± 0.6	261.85 ± 0.34	535.1 ± 0.7
282.0	605.1 ± 0.9	347.4 ± 1.9	323.6 ± 1.3	323.3 ± 0.7	266.70 ± 0.35	543.5 ± 0.7
282.25	610.8 ± 0.9	350.9 ± 2.0	327.6 ± 1.3	327.0 ± 0.7	271.64 ± 0.35	551.9 ± 0.7
282.5	616.5 ± 1.0	354.3 ± 2.1	331.7 ± 1.4	330.8 ± 0.7	276.7 ± 0.4	560.5 ± 0.7
282.75	622.2 ± 1.0	357.8 ± 2.2	335.8 ± 1.4	334.6 ± 0.7	281.8 ± 0.4	569.2 ± 0.8
283.0	628.0 ± 1.0	361.4 ± 2.3	340.0 ± 1.5	338.4 ± 0.8	286.9 ± 0.4	578.0 ± 0.8
283.25	633.8 ± 1.1	365.0 ± 2.4	344.2 ± 1.5	342.2 ± 0.8	292.2 ± 0.4	586.9 ± 0.8
283.5	639.7 ± 1.1	368.5 ± 2.4	348.5 ± 1.6	346.1 ± 0.8	297.6 ± 0.4	596.0 ± 0.9
283.75	645.6 ± 1.2	372.2 ± 2.5	352.8 ± 1.7	350.1 ± 0.8	303.0 ± 0.4	605.2 ± 0.9
284.0	651.6 ± 1.2	375.8 ± 2.6	357.2 ± 1.7	354.1 ± 0.9	308.6 ± 0.4	614.5 ± 0.9
284.25	657.6 ± 1.2	379.5 ± 2.7	361.6 ± 1.8	358.1 ± 0.9	314.2 ± 0.4	623.9 ± 1.0
284.5	663.6 ± 1.3	383.2 ± 2.8	366.0 ± 1.9	362.1 ± 0.9	319.9 ± 0.5	633.4 ± 1.0
284.75	669.7 ± 1.3	387.0 ± 2.9	370.5 ± 1.9	366.2 ± 0.9	325.7 ± 0.5	643.1 ± 1.0
285.0	675.9 ± 1.4	390.8 ± 3.0	375.1 ± 2.0	370.4 ± 1.0	331.6 ± 0.5	652.9 ± 1.1

Table 2: CONT: Exchange rates extrapolated to 310 K, 1M glucose at pH=6.21, using restricted model, with partial assignment.

T(K)	C_6	$C_{3/4}$	$C_{3/4}$	C_2	$C_{1,\alpha}$	$C_{1,\beta}$
285.25	682.0 ± 1.4	394.6 ± 3.1	379.7 ± 2.1	374.5 ± 1.0	337.6 ± 0.5	662.9 ± 1.1
285.5	688.3 ± 1.4	398.4 ± 3.2	384.3 ± 2.2	378.8 ± 1.0	343.7 ± 0.5	673.0 ± 1.1
285.75	694.6 ± 1.5	402.3 ± 3.3	389.0 ± 2.2	383.0 ± 1.1	349.9 ± 0.5	683.2 ± 1.2
286.0	700.9 ± 1.5	406.2 ± 3.4	393.7 ± 2.3	387.3 ± 1.1	356.3 ± 0.6	693.6 ± 1.2
286.25	707.3 ± 1.6	410.1 ± 3.5	398.5 ± 2.4	391.6 ± 1.1	362.7 ± 0.6	704.1 ± 1.3
286.5	713.7 ± 1.6	414 ± 4	403.4 ± 2.5	396.0 ± 1.2	369.2 ± 0.6	714.7 ± 1.3
286.75	720.2 ± 1.7	418 ± 4	408.3 ± 2.5	400.4 ± 1.2	375.8 ± 0.6	725.5 ± 1.3
287.0	726.7 ± 1.7	422 ± 4	413.2 ± 2.6	404.9 ± 1.2	382.5 ± 0.6	736.4 ± 1.4
287.25	733.2 ± 1.8	426 ± 4	418.2 ± 2.7	409.4 ± 1.3	389.4 ± 0.7	747.5 ± 1.4
287.5	739.8 ± 1.8	430 ± 4	423.2 ± 2.8	414.0 ± 1.3	396.3 ± 0.7	758.7 ± 1.5
287.75	746.5 ± 1.9	434 ± 4	428.3 ± 2.9	418.5 ± 1.3	403.4 ± 0.7	770.0 ± 1.5
288.0	753.2 ± 1.9	438 ± 4	433.5 ± 3.0	423.2 ± 1.4	410.5 ± 0.7	781.6 ± 1.6
288.25	760.0 ± 2.0	443 ± 4	438.7 ± 3.1	427.8 ± 1.4	417.8 ± 0.7	793.2 ± 1.6
288.5	766.8 ± 2.0	447 ± 5	443.9 ± 3.2	432.6 ± 1.4	425.2 ± 0.8	805.1 ± 1.7
288.75	773.6 ± 2.1	451 ± 5	449.2 ± 3.2	437.3 ± 1.5	432.7 ± 0.8	817.0 ± 1.7
289.0	780.6 ± 2.1	455 ± 5	454.6 ± 3.3	442.1 ± 1.5	440.4 ± 0.8	829.2 ± 1.8
289.25	787.5 ± 2.2	460 ± 5	460.0 ± 3.4	447.0 ± 1.6	448.1 ± 0.8	841.5 ± 1.8
289.5	794.5 ± 2.2	464 ± 5	465.5 ± 3.5	451.9 ± 1.6	456.0 ± 0.9	853.9 ± 1.9
289.75	801.6 ± 2.3	469 ± 5	471 ± 4	456.8 ± 1.6	464.0 ± 0.9	866.5 ± 2.0
290.0	808.7 ± 2.3	473 ± 5	477 ± 4	461.8 ± 1.7	472.2 ± 0.9	879.3 ± 2.0
290.25	815.9 ± 2.4	477 ± 5	482 ± 4	466.8 ± 1.7	480.4 ± 1.0	892.3 ± 2.1
290.5	823.1 ± 2.5	482 ± 6	488 ± 4	471.9 ± 1.8	488.8 ± 1.0	905.4 ± 2.1
290.75	830.3 ± 2.5	486 ± 6	494 ± 4	477.0 ± 1.8	497.4 ± 1.0	918.7 ± 2.2
291.0	837.7 ± 2.6	491 ± 6	500 ± 4	482.2 ± 1.9	506.0 ± 1.1	932.1 ± 2.3
291.25	845.0 ± 2.6	496 ± 6	505 ± 4	487.4 ± 1.9	514.8 ± 1.1	945.8 ± 2.3
291.5	852.5 ± 2.7	500 ± 6	511 ± 4	492.7 ± 2.0	523.8 ± 1.1	959.6 ± 2.4
291.75	859.9 ± 2.8	505 ± 6	517 ± 4	498.0 ± 2.0	532.9 ± 1.2	973.6 ± 2.5
292.0	867.5 ± 2.8	510 ± 6	523 ± 5	503.4 ± 2.1	542.1 ± 1.2	987.8 ± 2.5
292.25	875.0 ± 2.9	514 ± 7	530 ± 5	508.8 ± 2.1	551.4 ± 1.2	1002.1 ± 2.6
292.5	882.7 ± 2.9	519 ± 7	536 ± 5	514.3 ± 2.2	560.9 ± 1.3	1016.7 ± 2.7
292.75	890.4 ± 3.0	524 ± 7	542 ± 5	519.8 ± 2.2	570.6 ± 1.3	1031.4 ± 2.8
293.0	898.1 ± 3.1	529 ± 7	548 ± 5	525.3 ± 2.3	580.4 ± 1.3	1046.3 ± 2.8
293.25	905.9 ± 3.1	534 ± 7	555 ± 5	531.0 ± 2.3	590.4 ± 1.4	1061.4 ± 2.9
293.5	913.8 ± 3.2	539 ± 7	561 ± 5	536.6 ± 2.4	600.5 ± 1.4	1076.7 ± 3.0
293.75	921.7 ± 3.3	543 ± 8	568 ± 5	542.3 ± 2.4	610.7 ± 1.5	1092.2 ± 3.1
294.0	929.6 ± 3.3	548 ± 8	574 ± 6	548.1 ± 2.5	621.2 ± 1.5	1107.9 ± 3.1
294.25	937.7 ± 3.4	554 ± 8	581 ± 6	553.9 ± 2.5	631.8 ± 1.6	1123.8 ± 3.2
294.5	945.7 ± 3.5	559 ± 8	587 ± 6	559.8 ± 2.6	642.5 ± 1.6	1139.9 ± 3.3
294.75	954 ± 4	564 ± 8	594 ± 6	565.7 ± 2.6	653.4 ± 1.7	1156.1 ± 3.4
295.0	962 ± 4	569 ± 8	601 ± 6	571.7 ± 2.7	664.5 ± 1.7	1172.6 ± 3.5
295.25	970 ± 4	574 ± 9	608 ± 6	577.7 ± 2.8	675.7 ± 1.8	1189 ± 4
295.5	979 ± 4	579 ± 9	615 ± 6	583.8 ± 2.8	687.2 ± 1.8	1206 ± 4
295.75	987 ± 4	585 ± 9	622 ± 7	589.9 ± 2.9	698.8 ± 1.9	1223 ± 4
296.0	995 ± 4	590 ± 9	629 ± 7	596.1 ± 2.9	710.5 ± 1.9	1241 ± 4
296.25	1004 ± 4	595 ± 9	636 ± 7	602.4 ± 3.0	722.5 ± 2.0	1258 ± 4
296.5	1012 ± 4	601 ± 10	643 ± 7	608.7 ± 3.1	734.6 ± 2.0	1276 ± 4
296.75	1021 ± 4	606 ± 10	651 ± 7	615.0 ± 3.1	746.9 ± 2.1	1294 ± 4
297.0	1030 ± 4	611 ± 10	658 ± 7	621.4 ± 3.2	759.4 ± 2.1	1312 ± 4
297.25	1038 ± 4	617 ± 10	665 ± 8	627.9 ± 3.3	772.1 ± 2.2	1331 ± 4
297.5	1047 ± 4	622 ± 10	673 ± 8	634.4 ± 3.3	785.0 ± 2.3	1349 ± 4
297.75	1056 ± 4	628 ± 10	680 ± 8	641.0 ± 3.4	798.0 ± 2.3	1368 ± 5
298.0	1065 ± 5	634 ± 11	688 ± 8	647.6 ± 3.5	811.3 ± 2.4	1387 ± 5
298.25	1074 ± 5	639 ± 11	696 ± 8	654 ± 4	824.8 ± 2.5	1407 ± 5
298.5	1083 ± 5	645 ± 11	704 ± 8	661 ± 4	838.4 ± 2.5	1426 ± 5
298.75	1092 ± 5	651 ± 11	712 ± 9	668 ± 4	852.3 ± 2.6	1446 ± 5
299.0	1101 ± 5	657 ± 12	719 ± 9	675 ± 4	866.4 ± 2.7	1466 ± 5
299.25	1110 ± 5	662 ± 12	728 ± 9	682 ± 4	880.7 ± 2.7	1487 ± 5
299.5	1120 ± 5	668 ± 12	736 ± 9	689 ± 4	895.2 ± 2.8	1507 ± 5
299.75	1129 ± 5	674 ± 12	744 ± 9	696 ± 4	909.9 ± 2.9	1528 ± 6
300.0	1138 ± 5	680 ± 12	752 ± 9	703 ± 4	924.8 ± 3.0	1549 ± 6
300.25	1148 ± 5	686 ± 13	760 ± 10	710 ± 4	940.0 ± 3.0	1571 ± 6
300.5	1157 ± 5	692 ± 13	769 ± 10	717 ± 4	955.3 ± 3.1	1592 ± 6
300.75	1167 ± 6	698 ± 13	777 ± 10	725 ± 4	970.9 ± 3.2	1614 ± 6
301.0	1176 ± 6	705 ± 13	786 ± 10	732 ± 4	986.7 ± 3.3	1636 ± 6
301.25	1186 ± 6	711 ± 14	795 ± 10	739 ± 4	1002.8 ± 3.4	1659 ± 6
301.5	1196 ± 6	717 ± 14	803 ± 11	747 ± 5	1019.1 ± 3.5	1681 ± 7
301.75	1206 ± 6	723 ± 14	812 ± 11	754 ± 5	1035.6 ± 3.5	1704 ± 7
302.0	1216 ± 6	729 ± 14	821 ± 11	762 ± 5	1052 ± 4	1728 ± 7
302.25	1226 ± 6	736 ± 15	830 ± 11	770 ± 5	1069 ± 4	1751 ± 7
302.5	1236 ± 6	742 ± 15	839 ± 12	777 ± 5	1087 ± 4	1775 ± 7
302.75	1246 ± 6	749 ± 15	848 ± 12	785 ± 5	1104 ± 4	1799 ± 7
303.0	1256 ± 6	755 ± 15	857 ± 12	793 ± 5	1122 ± 4	1823 ± 7
303.25	1266 ± 7	762 ± 16	867 ± 12	801 ± 5	1140 ± 4	1848 ± 8
303.5	1276 ± 7	768 ± 16	876 ± 12	809 ± 5	1158 ± 4	1873 ± 8
303.75	1287 ± 7	775 ± 16	886 ± 13	817 ± 5	1177 ± 4	1898 ± 8
304.0	1297 ± 7	782 ± 16	895 ± 13	825 ± 5	1196 ± 4	1924 ± 8
304.25	1308 ± 7	788 ± 17	905 ± 13	834 ± 6	1215 ± 5	1950 ± 8
304.5	1318 ± 7	795 ± 17	915 ± 13	842 ± 6	1234 ± 5	1976 ± 8
304.75	1329 ± 7	802 ± 17	925 ± 14	850 ± 6	1254 ± 5	2002 ± 9
305.0	1340 ± 7	809 ± 17	935 ± 14	859 ± 6	1274 ± 5	2029 ± 9
305.25	1350 ± 7	816 ± 18	945 ± 14	867 ± 6	1294 ± 5	2056 ± 9
305.5	1361 ± 8	823 ± 18	955 ± 14	876 ± 6	1314 ± 5	2083 ± 9
305.75	1372 ± 8	830 ± 18	965 ± 15	884 ± 6	1335 ± 5	2111 ± 9
306.0	1383 ± 8	837 ± 19	975 ± 15	893 ± 6	1356 ± 5	2139 ± 10
306.25	1394 ± 8	844 ± 19	986 ± 15	902 ± 6	1377 ± 5	2168 ± 10
306.5	1405 ± 8	851 ± 19	996 ± 16	911 ± 7	1399 ± 6	2196 ± 10
306.75	1417 ± 8	858 ± 19	1007 ± 16	920 ± 7	1421 ± 6	2225 ± 10
307.0	1428 ± 8	866 ± 20	1017 ± 16	929 ± 7	1443 ± 6	2255 ± 10
307.25	1439 ± 8	873 ± 20	1028 ± 16	938 ± 7	1466 ± 6	2284 ± 11
307.5	1451 ± 8	880 ± 20	1039 ± 17	947 ± 7	1489 ± 6	2314 ± 11
307.75	1462 ± 9	888 ± 21	1050 ± 17	956 ± 7	1512 ± 6	2345 ± 11
308.0	1474 ± 9	895 ± 21	1061 ± 17	965 ± 7	1536 ± 6	2376 ± 11
308.25	1485 ± 9	903 ± 21	1072 ± 18	975 ± 7	1560 ± 7	2407 ± 12
308.5	1497 ± 9	910 ± 22	1084 ± 18	984 ± 7	1584 ± 7	2438 ± 12
308.75	1509 ± 9	918 ± 22	1095 ± 18	994 ± 8	1608 ± 7	2470 ± 12
309.0	1521 ± 9	926 ± 22	1107 ± 18	1003 ± 8	1633 ± 7	2502 ± 12
309.25	1533 ± 9	933 ± 23	1118 ± 19	1013 ± 8	1658 ± 7	2535 ± 12
309.5	1545 ± 10	941 ± 23	1130 ± 19	1023 ± 8	1684 ± 7	2568 ± 13
309.75	1557 ± 10	949 ± 23	1142 ± 19	1032 ± 8	1710 ± 8	2601 ± 13
310.0	1569 ± 10	957 ± 24	1154 ± 20	1042 ± 8	1736 ± 8	2635 ± 13

Table 3: Exchange rates extrapolated to 310 K, 1M glucose at pH=7.00, using restricted model, with partial assignment.

T(K)	C_6	$C_{3/4}$	$C_{3/4}$	C_2	$C_{1,\alpha}$	$C_{1,\beta}$
260.0	175.02 ± 0.27	125.0 ± 0.5	110.6 ± 0.5	112.2 ± 0.4	140.8 ± 0.5	280.4 ± 0.9
260.25	177.39 ± 0.26	126.9 ± 0.5	112.7 ± 0.5	114.0 ± 0.4	144.1 ± 0.5	286.4 ± 0.9
260.5	179.79 ± 0.26	128.9 ± 0.5	114.8 ± 0.5	115.9 ± 0.4	147.4 ± 0.5	292.5 ± 0.9
260.75	182.21 ± 0.26	130.8 ± 0.5	116.9 ± 0.5	117.9 ± 0.4	150.9 ± 0.5	298.7 ± 0.9
261.0	184.67 ± 0.26	132.8 ± 0.5	119.2 ± 0.5	119.8 ± 0.4	154.4 ± 0.5	305.1 ± 0.9
261.25	187.15 ± 0.25	134.8 ± 0.5	121.4 ± 0.5	121.8 ± 0.4	157.9 ± 0.5	311.5 ± 0.9
261.5	189.66 ± 0.25	136.9 ± 0.5	123.7 ± 0.5	123.8 ± 0.4	161.6 ± 0.5	318.1 ± 0.9
261.75	192.20 ± 0.25	138.9 ± 0.5	126.0 ± 0.5	125.9 ± 0.4	165.3 ± 0.5	324.9 ± 0.9
262.0	194.77 ± 0.24	141.0 ± 0.5	128.3 ± 0.5	127.9 ± 0.4	169.1 ± 0.5	331.7 ± 0.8
262.25	197.37 ± 0.24	143.1 ± 0.5	130.7 ± 0.4	130.0 ± 0.4	173.0 ± 0.5	338.7 ± 0.8
262.5	199.99 ± 0.24	145.3 ± 0.5	133.2 ± 0.4	132.2 ± 0.4	176.9 ± 0.5	345.9 ± 0.8
262.75	202.65 ± 0.23	147.5 ± 0.5	135.6 ± 0.4	134.3 ± 0.4	181.0 ± 0.5	353.1 ± 0.8
263.0	205.34 ± 0.23	149.7 ± 0.4	138.2 ± 0.4	136.53 ± 0.35	185.1 ± 0.5	360.5 ± 0.8
263.25	208.06 ± 0.22	151.9 ± 0.4	140.7 ± 0.4	138.76 ± 0.35	189.3 ± 0.5	368.1 ± 0.8
263.5	210.81 ± 0.22	154.2 ± 0.4	143.3 ± 0.4	141.02 ± 0.34	193.6 ± 0.5	375.8 ± 0.8
263.75	213.59 ± 0.22	156.5 ± 0.4	146.0 ± 0.4	143.31 ± 0.33	198.0 ± 0.5	383.6 ± 0.8
264.0	216.40 ± 0.21	158.8 ± 0.4	148.7 ± 0.4	145.64 ± 0.33	202.5 ± 0.5	391.6 ± 0.8
264.25	219.25 ± 0.21	161.1 ± 0.4	151.4 ± 0.4	148.00 ± 0.32	207.1 ± 0.5	399.7 ± 0.8
264.5	222.12 ± 0.20	163.5 ± 0.4	154.2 ± 0.4	150.39 ± 0.31	211.8 ± 0.5	408.0 ± 0.8
264.75	225.03 ± 0.20	165.9 ± 0.4	157.0 ± 0.4	152.82 ± 0.31	216.5 ± 0.5	416.4 ± 0.7
265.0	227.98 ± 0.19	168.4 ± 0.4	159.86 ± 0.35	155.28 ± 0.30	221.4 ± 0.5	425.0 ± 0.7
265.25	230.95 ± 0.19	170.8 ± 0.4	162.77 ± 0.34	157.77 ± 0.29	226.4 ± 0.5	433.8 ± 0.7
265.5	233.96 ± 0.18	173.35 ± 0.35	165.74 ± 0.33	160.31 ± 0.29	231.4 ± 0.5	442.7 ± 0.7
265.75	237.00 ± 0.18	175.89 ± 0.34	168.75 ± 0.32	162.87 ± 0.28	236.6 ± 0.4	451.8 ± 0.7
266.0	240.08 ± 0.17	178.46 ± 0.33	171.81 ± 0.31	165.48 ± 0.27	241.9 ± 0.4	461.0 ± 0.7
266.25	243.19 ± 0.17	181.07 ± 0.31	174.92 ± 0.30	168.12 ± 0.26	247.3 ± 0.4	470.5 ± 0.7
266.5	246.33 ± 0.16	183.70 ± 0.30	178.09 ± 0.29	170.80 ± 0.26	252.8 ± 0.4	480.1 ± 0.7
266.75	249.51 ± 0.16	186.38 ± 0.29	181.30 ± 0.28	173.51 ± 0.25	258.4 ± 0.4	489.9 ± 0.6
267.0	252.72 ± 0.16	189.08 ± 0.28	184.57 ± 0.27	176.27 ± 0.24	264.1 ± 0.4	499.8 ± 0.6
267.25	255.97 ± 0.15	191.82 ± 0.27	187.88 ± 0.26	179.06 ± 0.24	269.9 ± 0.4	510.0 ± 0.6
267.5	259.26 ± 0.15	194.60 ± 0.26	191.26 ± 0.25	181.89 ± 0.23	275.9 ± 0.4	520.3 ± 0.6
267.75	262.58 ± 0.14	197.41 ± 0.25	194.68 ± 0.24	184.76 ± 0.22	282.0 ± 0.4	530.9 ± 0.6
268.0	265.94 ± 0.14	200.25 ± 0.25	198.16 ± 0.23	187.67 ± 0.22	288.2 ± 0.4	541.6 ± 0.6
268.25	269.34 ± 0.13	203.13 ± 0.24	201.70 ± 0.23	190.62 ± 0.21	294.5 ± 0.4	552.5 ± 0.6
268.5	272.77 ± 0.13	206.05 ± 0.23	205.29 ± 0.22	193.62 ± 0.21	300.93 ± 0.35	563.6 ± 0.6
268.75	276.24 ± 0.13	209.00 ± 0.23	208.94 ± 0.22	196.65 ± 0.20	307.51 ± 0.35	574.9 ± 0.6
269.0	279.75 ± 0.13	211.99 ± 0.23	212.65 ± 0.22	199.72 ± 0.20	314.22 ± 0.35	586.5 ± 0.5
269.25	283.29 ± 0.12	215.02 ± 0.23	216.42 ± 0.22	202.84 ± 0.20	321.07 ± 0.35	598.2 ± 0.5
269.5	286.88 ± 0.12	218.08 ± 0.23	220.24 ± 0.22	206.00 ± 0.20	328.05 ± 0.35	610.1 ± 0.5
269.75	290.50 ± 0.12	221.19 ± 0.23	224.13 ± 0.23	209.21 ± 0.20	335.16 ± 0.35	622.3 ± 0.5
270.0	294.16 ± 0.12	224.33 ± 0.24	228.08 ± 0.24	212.46 ± 0.21	342.43 ± 0.35	634.7 ± 0.5
270.25	297.86 ± 0.12	227.51 ± 0.25	232.09 ± 0.25	215.75 ± 0.21	349.8 ± 0.4	647.3 ± 0.6
270.5	301.60 ± 0.13	230.73 ± 0.26	236.17 ± 0.27	219.09 ± 0.22	357.4 ± 0.4	660.1 ± 0.6
270.75	305.39 ± 0.13	233.99 ± 0.27	240.31 ± 0.28	222.47 ± 0.23	365.1 ± 0.4	673.2 ± 0.6
271.0	309.21 ± 0.13	237.29 ± 0.29	244.51 ± 0.30	225.90 ± 0.24	372.9 ± 0.4	686.5 ± 0.6
271.25	313.07 ± 0.14	240.63 ± 0.30	248.78 ± 0.33	229.38 ± 0.25	380.9 ± 0.4	700.0 ± 0.6
271.5	316.98 ± 0.14	244.01 ± 0.32	253.11 ± 0.35	232.90 ± 0.26	389.1 ± 0.4	713.8 ± 0.7
271.75	320.92 ± 0.15	247.43 ± 0.34	257.5 ± 0.4	236.47 ± 0.27	397.4 ± 0.5	727.8 ± 0.7
272.0	324.91 ± 0.16	250.9 ± 0.4	262.0 ± 0.4	240.09 ± 0.29	405.9 ± 0.5	742.1 ± 0.7
272.25	328.94 ± 0.17	254.4 ± 0.4	266.5 ± 0.4	243.76 ± 0.31	414.5 ± 0.5	756.6 ± 0.8
272.5	333.02 ± 0.17	258.0 ± 0.4	271.1 ± 0.5	247.48 ± 0.33	423.4 ± 0.6	771.4 ± 0.8
272.75	337.13 ± 0.18	261.5 ± 0.4	275.8 ± 0.5	251.24 ± 0.34	432.4 ± 0.6	786.5 ± 0.9
273.0	341.29 ± 0.19	265.2 ± 0.5	280.6 ± 0.5	255.1 ± 0.4	441.5 ± 0.6	801.8 ± 0.9
273.25	345.50 ± 0.20	268.9 ± 0.5	285.4 ± 0.6	258.9 ± 0.4	450.9 ± 0.7	817.4 ± 1.0
273.5	349.74 ± 0.22	272.6 ± 0.5	290.3 ± 0.6	262.9 ± 0.4	460.4 ± 0.7	833.2 ± 1.1
273.75	354.04 ± 0.23	276.4 ± 0.6	295.3 ± 0.6	266.8 ± 0.4	470.1 ± 0.8	849.4 ± 1.1
274.0	358.38 ± 0.24	280.2 ± 0.6	300.3 ± 0.7	270.9 ± 0.5	480.0 ± 0.8	865.8 ± 1.2
274.25	362.76 ± 0.25	284.0 ± 0.6	305.5 ± 0.7	274.9 ± 0.5	490.1 ± 0.9	882.5 ± 1.3
274.5	367.19 ± 0.26	287.9 ± 0.7	310.7 ± 0.8	279.1 ± 0.5	500.3 ± 0.9	899.5 ± 1.3
274.75	371.66 ± 0.28	291.9 ± 0.7	316.0 ± 0.8	283.3 ± 0.5	510.8 ± 1.0	916.8 ± 1.4
275.0	376.18 ± 0.29	295.9 ± 0.7	321.4 ± 0.9	287.5 ± 0.6	521.5 ± 1.1	934.4 ± 1.5
275.25	380.75 ± 0.31	299.9 ± 0.8	326.8 ± 0.9	291.8 ± 0.6	532.3 ± 1.1	952.3 ± 1.6
275.5	385.37 ± 0.32	304.0 ± 0.8	332.3 ± 0.9	296.1 ± 0.6	543.4 ± 1.2	970.5 ± 1.7
275.75	390.03 ± 0.34	308.2 ± 0.8	338.0 ± 1.0	300.6 ± 0.7	554.7 ± 1.2	989.0 ± 1.8
276.0	394.75 ± 0.35	312.4 ± 0.9	343.7 ± 1.0	305.0 ± 0.7	566.2 ± 1.3	1007.9 ± 1.8
276.25	399.5 ± 0.4	316.6 ± 0.9	349.4 ± 1.1	309.6 ± 0.7	577.9 ± 1.4	1027.0 ± 1.9
276.5	404.3 ± 0.4	320.9 ± 1.0	355.3 ± 1.2	314.1 ± 0.8	589.9 ± 1.5	1046.5 ± 2.0
276.75	409.2 ± 0.4	325.3 ± 1.0	361.3 ± 1.2	318.8 ± 0.8	602.0 ± 1.5	1066.4 ± 2.1
277.0	414.1 ± 0.4	329.7 ± 1.1	367.3 ± 1.3	323.5 ± 0.8	614.4 ± 1.6	1086.6 ± 2.3
277.25	419.0 ± 0.4	334.1 ± 1.1	373.5 ± 1.3	328.3 ± 0.9	627.1 ± 1.7	1107.1 ± 2.4
277.5	424.1 ± 0.5	338.6 ± 1.1	379.7 ± 1.4	333.1 ± 0.9	639.9 ± 1.8	1128.0 ± 2.5
277.75	429.1 ± 0.5	343.1 ± 1.2	386.1 ± 1.4	338.0 ± 0.9	653.0 ± 1.9	1149.2 ± 2.6
278.0	434.2 ± 0.5	347.8 ± 1.2	392.5 ± 1.5	343.0 ± 1.0	666.4 ± 2.0	1170.8 ± 2.7
278.25	439.4 ± 0.5	352.4 ± 1.3	399.0 ± 1.6	348.0 ± 1.0	680.0 ± 2.1	1192.7 ± 2.8
278.5	444.6 ± 0.5	357.1 ± 1.3	405.6 ± 1.6	353.1 ± 1.1	693.9 ± 2.2	1215.1 ± 3.0
278.75	449.9 ± 0.6	361.9 ± 1.4	412.3 ± 1.7	358.2 ± 1.1	708.0 ± 2.3	1237.8 ± 3.1
279.0	455.2 ± 0.6	366.7 ± 1.4	419.1 ± 1.8	363.4 ± 1.1	722.3 ± 2.4	1260.9 ± 3.2
279.25	460.6 ± 0.6	371.6 ± 1.5	426.0 ± 1.9	368.7 ± 1.2	737.0 ± 2.5	1284.3 ± 3.4
279.5	466.0 ± 0.6	376.5 ± 1.6	433.1 ± 1.9	374.1 ± 1.2	751.9 ± 2.6	1308.2 ± 3.5
279.75	471.5 ± 0.6	381.5 ± 1.6	440.2 ± 2.0	379.5 ± 1.3	767.1 ± 2.7	1332 ± 4
280.0	477.0 ± 0.7	386.6 ± 1.7	447.4 ± 2.1	385.0 ± 1.3	782.5 ± 2.8	1357 ± 4
280.25	482.6 ± 0.7	391.7 ± 1.7	454.7 ± 2.2	390.5 ± 1.4	798.3 ± 2.9	1382 ± 4
280.5	488.3 ± 0.7	396.8 ± 1.8	462.2 ± 2.2	396.2 ± 1.4	814.3 ± 3.0	1408 ± 4
280.75	494.0 ± 0.7	402.1 ± 1.9	469.7 ± 2.3	401.9 ± 1.5	830.6 ± 3.2	1434 ± 4
281.0	499.7 ± 0.8	407.3 ± 1.9	477.4 ± 2.4	407.6 ± 1.5	847.3 ± 3.3	1460 ± 4
281.25	505.6 ± 0.8	412.7 ± 2.0	485.1 ± 2.5	413.5 ± 1.6	864.2 ± 3.4	1487 ± 5
281.5	511.4 ± 0.8	418.1 ± 2.0	493.0 ± 2.6	419.4 ± 1.6	881 ± 4	1514 ± 5
281.75	517.4 ± 0.8	423.6 ± 2.1	501.0 ± 2.7	425.4 ± 1.7	899 ± 4	1542 ± 5
282.0	523.4 ± 0.9	429.1 ± 2.2	509.1 ± 2.8	431.5 ± 1.8	917 ± 4	1570 ± 5
282.25	529.4 ± 0.9	434.7 ± 2.2	517.3 ± 2.9	437.6 ± 1.8	935 ± 4	1599 ± 5
282.5	535.5 ± 0.9	440.3 ± 2.3	525.6 ± 3.0	443.8 ± 1.9	954 ± 4	1628 ± 6
282.75	541.7 ± 0.9	446.0 ± 2.4	534.1 ± 3.1	450.1 ± 1.9	972 ± 4	1657 ± 6
283.0	548.0 ± 1.0	451.8 ± 2.5	542.7 ± 3.2	456.5 ± 2.0	992 ± 4	1687 ± 6
283.25	554.3 ± 1.0	457.7 ± 2.5	551.4 ± 3.3	462.9 ± 2.1	1011 ± 5	1718 ± 6
283.5	560.6 ± 1.0	463.6 ± 2.6	560.2 ± 3.4	469.5 ± 2.1	1031 ± 5	1749 ± 6
283.75	567.0 ± 1.0	469.6 ± 2.7	569.2 ± 3.5	476.1 ± 2.2	1051 ± 5	1781 ± 7
284.0	573.5 ± 1.1	475.6 ± 2.8	578 ± 4	482.8 ± 2.3	1072 ± 5	1813 ± 7
284.25	580.1 ± 1.1	481.7 ± 2.8	587 ± 4	489.6 ± 2.3	1093 ± 5	1845 ± 7
284.5	586.7 ± 1.1	487.9 ± 2.9	597 ± 4	496.4 ± 2.4	1114 ± 6	1878 ± 7
284.75	593.3 ± 1.2	494.2 ± 3.0	606 ± 4	503.4 ± 2.5	1136 ± 6	1912 ± 8
285.0	600.1 ± 1.2	500.5 ± 3.1	616 ± 4	510.4 ± 2.5	1158 ± 6	1946 ± 8

Table 4: CONT: Exchange rates extrapolated to 310 K, 1M glucose at pH=7.00, using restricted model, with partial assignment.

T(K)	C_6	$C_{3/4}$	$C_{3/4}$	C_2	$C_{1,\alpha}$	$C_{1,\beta}$
285.25	606.9 ± 1.2	506.9 ± 3.2	626 ± 4	517.5 ± 2.6	1181 ± 6	1981 ± 8
285.5	613.7 ± 1.3	513.3 ± 3.3	636 ± 4	524.7 ± 2.7	1203 ± 6	2016 ± 8
285.75	620.7 ± 1.3	519.8 ± 3.4	646 ± 4	532.0 ± 2.8	1227 ± 7	2052 ± 9
286.0	627.7 ± 1.3	526.4 ± 3.4	656 ± 5	539.4 ± 2.8	1250 ± 7	2089 ± 9
286.25	634.7 ± 1.4	533.1 ± 3.5	666 ± 5	546.9 ± 2.9	1275 ± 7	2126 ± 9
286.5	641.9 ± 1.4	540 ± 4	676 ± 5	554.5 ± 3.0	1299 ± 7	2163 ± 10
286.75	649.1 ± 1.4	547 ± 4	687 ± 5	562.1 ± 3.1	1324 ± 8	2202 ± 10
287.0	656.3 ± 1.5	554 ± 4	698 ± 5	569.9 ± 3.2	1350 ± 8	2240 ± 10
287.25	663.7 ± 1.5	561 ± 4	709 ± 5	577.7 ± 3.3	1375 ± 8	2280 ± 11
287.5	671.1 ± 1.5	568 ± 4	720 ± 5	585.6 ± 3.3	1402 ± 8	2320 ± 11
287.75	678.6 ± 1.6	575 ± 4	731 ± 6	593.7 ± 3.4	1429 ± 9	2361 ± 11
288.0	686.1 ± 1.6	582 ± 4	742 ± 6	601.8 ± 3.5	1456 ± 9	2402 ± 12
288.25	693.7 ± 1.7	589 ± 4	754 ± 6	610 ± 4	1483 ± 9	2444 ± 12
288.5	701.4 ± 1.7	597 ± 4	765 ± 6	618 ± 4	1512 ± 9	2487 ± 12
288.75	709.2 ± 1.7	604 ± 5	777 ± 6	627 ± 4	1540 ± 10	2530 ± 13
289.0	717.0 ± 1.8	612 ± 5	789 ± 6	635 ± 4	1570 ± 10	2574 ± 13
289.25	725.0 ± 1.8	619 ± 5	801 ± 7	644 ± 4	1599 ± 10	2619 ± 13
289.5	732.9 ± 1.9	627 ± 5	814 ± 7	653 ± 4	1629 ± 11	2664 ± 14
289.75	741.0 ± 1.9	635 ± 5	826 ± 7	661 ± 4	1660 ± 11	2710 ± 14
290.0	749.1 ± 1.9	642 ± 5	839 ± 7	670 ± 4	1691 ± 11	2757 ± 15
290.25	757.3 ± 2.0	650 ± 5	852 ± 7	679 ± 4	1723 ± 12	2805 ± 15
290.5	765.6 ± 2.0	658 ± 5	865 ± 8	689 ± 5	1755 ± 12	2853 ± 16
290.75	774.0 ± 2.1	666 ± 5	878 ± 8	698 ± 5	1788 ± 12	2902 ± 16
291.0	782.4 ± 2.1	675 ± 6	891 ± 8	707 ± 5	1822 ± 13	2952 ± 16
291.25	791.0 ± 2.2	683 ± 6	905 ± 8	717 ± 5	1856 ± 13	3003 ± 17
291.5	799.6 ± 2.2	691 ± 6	918 ± 8	726 ± 5	1890 ± 14	3054 ± 17
291.75	808.2 ± 2.3	700 ± 6	932 ± 9	736 ± 5	1925 ± 14	3106 ± 18
292.0	817.0 ± 2.3	708 ± 6	946 ± 9	746 ± 5	1961 ± 14	3159 ± 18
292.25	825.8 ± 2.4	717 ± 6	961 ± 9	756 ± 5	1997 ± 15	3213 ± 19
292.5	834.7 ± 2.4	726 ± 6	975 ± 9	766 ± 5	2034 ± 15	3268 ± 19
292.75	843.7 ± 2.5	734 ± 7	990 ± 10	776 ± 6	2072 ± 16	3323 ± 20
293.0	852.8 ± 2.5	743 ± 7	1005 ± 10	786 ± 6	2110 ± 16	3379 ± 20
293.25	862.0 ± 2.6	752 ± 7	1020 ± 10	797 ± 6	2149 ± 17	3437 ± 21
293.5	871.2 ± 2.6	761 ± 7	1035 ± 10	807 ± 6	2188 ± 17	3495 ± 22
293.75	880.6 ± 2.7	770 ± 7	1050 ± 10	818 ± 6	2228 ± 18	3553 ± 22
294.0	890.0 ± 2.7	780 ± 7	1066 ± 11	828 ± 6	2269 ± 18	3613 ± 23
294.25	899.5 ± 2.8	789 ± 7	1082 ± 11	839 ± 6	2311 ± 18	3674 ± 23
294.5	909.1 ± 2.8	799 ± 8	1098 ± 11	850 ± 7	2353 ± 19	3736 ± 24
294.75	918.7 ± 2.9	808 ± 8	1114 ± 12	861 ± 7	2396 ± 20	3798 ± 25
295.0	928.5 ± 3.0	818 ± 8	1131 ± 12	873 ± 7	2439 ± 20	3862 ± 25
295.25	938.3 ± 3.0	828 ± 8	1148 ± 12	884 ± 7	2484 ± 21	3926 ± 26
295.5	948.3 ± 3.1	837 ± 8	1164 ± 12	896 ± 7	2529 ± 21	3991 ± 27
295.75	958.3 ± 3.1	847 ± 8	1182 ± 13	907 ± 7	2574 ± 22	4058 ± 27
296.0	968.4 ± 3.2	857 ± 9	1199 ± 13	919 ± 7	2621 ± 22	4125 ± 28
296.25	978.6 ± 3.3	868 ± 9	1217 ± 13	931 ± 8	2668 ± 23	4194 ± 29
296.5	988.9 ± 3.3	878 ± 9	1234 ± 14	943 ± 8	2716 ± 24	4263 ± 29
296.75	999.3 ± 3.4	888 ± 9	1253 ± 14	955 ± 8	2765 ± 24	4333 ± 30
297.0	1009.8 ± 3.4	899 ± 9	1271 ± 14	967 ± 8	2815 ± 25	4405 ± 31
297.25	1020.3 ± 3.5	909 ± 10	1289 ± 15	980 ± 8	2865 ± 25	4477 ± 32
297.5	1031 ± 4	920 ± 10	1308 ± 15	992 ± 8	2916 ± 26	4551 ± 32
297.75	1042 ± 4	931 ± 10	1327 ± 15	1005 ± 9	2968 ± 27	4626 ± 33
298.0	1053 ± 4	942 ± 10	1346 ± 16	1018 ± 9	3021 ± 28	4702 ± 34
298.25	1064 ± 4	953 ± 10	1366 ± 16	1031 ± 9	3075 ± 28	4778 ± 35
298.5	1075 ± 4	964 ± 11	1386 ± 16	1044 ± 9	3130 ± 29	$(4.86 \pm 0.04) \times 10^3$
298.75	1086 ± 4	975 ± 11	1406 ± 17	1058 ± 9	3185 ± 30	$(4.94 \pm 0.04) \times 10^3$
299.0	1097 ± 4	987 ± 11	1426 ± 17	1071 ± 10	3242 ± 30	$(5.02 \pm 0.04) \times 10^3$
299.25	1108 ± 4	998 ± 11	1447 ± 17	1085 ± 10	3299 ± 31	$(5.10 \pm 0.04) \times 10^3$
299.5	1120 ± 4	1010 ± 11	1467 ± 18	1098 ± 10	3357 ± 32	$(5.18 \pm 0.04) \times 10^3$
299.75	1131 ± 4	1021 ± 12	1488 ± 18	1112 ± 10	3416 ± 33	$(5.26 \pm 0.04) \times 10^3$
300.0	1143 ± 4	1033 ± 12	1510 ± 18	1126 ± 10	3476 ± 34	$(5.35 \pm 0.04) \times 10^3$
300.25	1155 ± 4	1045 ± 12	1531 ± 19	1141 ± 11	3537 ± 35	$(5.44 \pm 0.04) \times 10^3$
300.5	1166 ± 4	1057 ± 12	1553 ± 19	1155 ± 11	3599 ± 35	$(5.52 \pm 0.04) \times 10^3$
300.75	1178 ± 5	1069 ± 13	1575 ± 20	1169 ± 11	$(3.66 \pm 0.04) \times 10^3$	$(5.61 \pm 0.04) \times 10^3$
301.0	1190 ± 5	1082 ± 13	1598 ± 20	1184 ± 11	$(3.73 \pm 0.04) \times 10^3$	$(5.70 \pm 0.05) \times 10^3$
301.25	1203 ± 5	1094 ± 13	1621 ± 21	1199 ± 11	$(3.79 \pm 0.04) \times 10^3$	$(5.79 \pm 0.05) \times 10^3$
301.5	1215 ± 5	1107 ± 13	1644 ± 21	1214 ± 12	$(3.86 \pm 0.04) \times 10^3$	$(5.89 \pm 0.05) \times 10^3$
301.75	1227 ± 5	1119 ± 13	1667 ± 21	1229 ± 12	$(3.92 \pm 0.04) \times 10^3$	$(5.98 \pm 0.05) \times 10^3$
302.0	1240 ± 5	1132 ± 14	1690 ± 22	1245 ± 12	$(3.99 \pm 0.04) \times 10^3$	$(6.08 \pm 0.05) \times 10^3$
302.25	1252 ± 5	1145 ± 14	1714 ± 22	1260 ± 12	$(4.06 \pm 0.04) \times 10^3$	$(6.17 \pm 0.05) \times 10^3$
302.5	1265 ± 5	1158 ± 14	1738 ± 23	1276 ± 13	$(4.13 \pm 0.04) \times 10^3$	$(6.27 \pm 0.05) \times 10^3$
302.75	1278 ± 5	1171 ± 14	1763 ± 23	1292 ± 13	$(4.20 \pm 0.04) \times 10^3$	$(6.37 \pm 0.05) \times 10^3$
303.0	1291 ± 5	1184 ± 15	1788 ± 24	1308 ± 13	$(4.28 \pm 0.05) \times 10^3$	$(6.47 \pm 0.05) \times 10^3$
303.25	1304 ± 5	1198 ± 15	1813 ± 24	1324 ± 13	$(4.35 \pm 0.05) \times 10^3$	$(6.57 \pm 0.06) \times 10^3$
303.5	1317 ± 5	1211 ± 15	1838 ± 25	1340 ± 14	$(4.42 \pm 0.05) \times 10^3$	$(6.68 \pm 0.06) \times 10^3$
303.75	1330 ± 6	1225 ± 16	1864 ± 25	1357 ± 14	$(4.50 \pm 0.05) \times 10^3$	$(6.78 \pm 0.06) \times 10^3$
304.0	1343 ± 6	1239 ± 16	1890 ± 26	1373 ± 14	$(4.58 \pm 0.05) \times 10^3$	$(6.89 \pm 0.06) \times 10^3$
304.25	1357 ± 6	1253 ± 16	1916 ± 26	1390 ± 14	$(4.66 \pm 0.05) \times 10^3$	$(7.00 \pm 0.06) \times 10^3$
304.5	1370 ± 6	1267 ± 16	1943 ± 27	1407 ± 15	$(4.74 \pm 0.05) \times 10^3$	$(7.11 \pm 0.06) \times 10^3$
304.75	1384 ± 6	1281 ± 17	1970 ± 27	1425 ± 15	$(4.82 \pm 0.05) \times 10^3$	$(7.22 \pm 0.06) \times 10^3$
305.0	1398 ± 6	1296 ± 17	1997 ± 28	1442 ± 15	$(4.90 \pm 0.05) \times 10^3$	$(7.33 \pm 0.06) \times 10^3$
305.25	1412 ± 6	1310 ± 17	2025 ± 29	1460 ± 16	$(4.98 \pm 0.06) \times 10^3$	$(7.45 \pm 0.07) \times 10^3$
305.5	1426 ± 6	1325 ± 18	2053 ± 29	1477 ± 16	$(5.07 \pm 0.06) \times 10^3$	$(7.56 \pm 0.07) \times 10^3$
305.75	1440 ± 6	1339 ± 18	2081 ± 30	1495 ± 16	$(5.15 \pm 0.06) \times 10^3$	$(7.68 \pm 0.07) \times 10^3$
306.0	1454 ± 6	1354 ± 18	2110 ± 30	1514 ± 16	$(5.24 \pm 0.06) \times 10^3$	$(7.80 \pm 0.07) \times 10^3$
306.25	1468 ± 7	1369 ± 18	2139 ± 31	1532 ± 17	$(5.33 \pm 0.06) \times 10^3$	$(7.92 \pm 0.07) \times 10^3$
306.5	1483 ± 7	1385 ± 19	2169 ± 31	1551 ± 17	$(5.42 \pm 0.06) \times 10^3$	$(8.05 \pm 0.07) \times 10^3$
306.75	1497 ± 7	1400 ± 19	2198 ± 32	1569 ± 17	$(5.51 \pm 0.06) \times 10^3$	$(8.17 \pm 0.08) \times 10^3$
307.0	1512 ± 7	1415 ± 19	2228 ± 33	1588 ± 18	$(5.60 \pm 0.07) \times 10^3$	$(8.30 \pm 0.08) \times 10^3$
307.25	1527 ± 7	1431 ± 20	2259 ± 33	1608 ± 18	$(5.70 \pm 0.07) \times 10^3$	$(8.42 \pm 0.08) \times 10^3$
307.5	1542 ± 7	1447 ± 20	2290 ± 34	1627 ± 18	$(5.79 \pm 0.07) \times 10^3$	$(8.55 \pm 0.08) \times 10^3$
307.75	1557 ± 7	1463 ± 20	2321 ± 35	1646 ± 19	$(5.89 \pm 0.07) \times 10^3$	$(8.69 \pm 0.08) \times 10^3$
308.0	1572 ± 7	1479 ± 21	2353 ± 35	1666 ± 19	$(5.99 \pm 0.07) \times 10^3$	$(8.82 \pm 0.08) \times 10^3$
308.25	1588 ± 7	1495 ± 21	$(2.38 \pm 0.04) \times 10^3$	1686 ± 19	$(6.09 \pm 0.07) \times 10^3$	$(8.95 \pm 0.09) \times 10^3$
308.5	1603 ± 7	1512 ± 21	$(2.42 \pm 0.04) \times 10^3$	1706 ± 20	$(6.19 \pm 0.07) \times 10^3$	$(9.09 \pm 0.09) \times 10^3$
308.75	1619 ± 8	1528 ± 22	$(2.45 \pm 0.04) \times 10^3$	1727 ± 20	$(6.29 \pm 0.08) \times 10^3$	$(9.23 \pm 0.09) \times 10^3$
309.0	1634 ± 8	1545 ± 22	$(2.48 \pm 0.04) \times 10^3$	1747 ± 20	$(6.39 \pm 0.08) \times 10^3$	$(9.37 \pm 0.09) \times 10^3$
309.25	1650 ± 8	1562 ± 23	$(2.52 \pm 0.04) \times 10^3$	1768 ± 21	$(6.50 \pm 0.08) \times 10^3$	$(9.51 \pm 0.09) \times 10^3$
309.5	1666 ± 8	1579 ± 23	$(2.55 \pm 0.04) \times 10^3$	1789 ± 21	$(6.61 \pm 0.08) \times 10^3$	$(9.66 \pm 0.09) \times 10^3$
309.75	1682 ± 8	1596 ± 23	$(2.58 \pm 0.04) \times 10^3$	1811 ± 21	$(6.72 \pm 0.08) \times 10^3$	$(9.81 \pm 0.10) \times 10^3$
310.0	1699 ± 8	1613 ± 24	$(2.62 \pm 0.04) \times 10^3$	1832 ± 22	$(6.83 \pm 0.09) \times 10^3$	$(9.95 \pm 0.10) \times 10^3$

Table 5: Exchange rates extrapolated to 310 K, 1M glucose at pH=7.38, using restricted model, with partial assignment.

T(K)	C_6	$C_{3/4}$	$C_{3/4}$	C_2	$C_{1,\alpha}$	$C_{1,\beta}$
260.0	162.0 ± 0.7	111.0 ± 2.2	176.4 ± 2.1	99.7 ± 1.3	222.3 ± 3.0	478 ± 6
260.25	165.5 ± 0.7	114.5 ± 2.2	179.5 ± 2.1	103.0 ± 1.3	229.1 ± 3.0	490 ± 6
260.5	169.0 ± 0.7	118.2 ± 2.2	182.6 ± 2.0	106.3 ± 1.3	236.1 ± 3.0	501 ± 6
260.75	172.6 ± 0.7	121.9 ± 2.2	185.8 ± 2.0	109.8 ± 1.3	243.3 ± 3.0	514 ± 6
261.0	176.3 ± 0.7	125.8 ± 2.2	189.0 ± 2.0	113.3 ± 1.3	250.6 ± 3.0	526 ± 6
261.25	180.1 ± 0.7	129.7 ± 2.2	192.3 ± 2.0	117.0 ± 1.3	258.2 ± 3.0	539 ± 6
261.5	183.9 ± 0.7	133.8 ± 2.2	195.6 ± 2.0	120.8 ± 1.3	266.0 ± 3.0	552 ± 6
261.75	187.8 ± 0.7	138.0 ± 2.2	199.0 ± 2.0	124.7 ± 1.3	274.0 ± 3.0	566 ± 6
262.0	191.7 ± 0.7	142.3 ± 2.2	202.4 ± 1.9	128.7 ± 1.3	282.3 ± 3.0	579 ± 6
262.25	195.8 ± 0.7	146.8 ± 2.2	205.9 ± 1.9	132.8 ± 1.3	290.8 ± 3.0	593 ± 6
262.5	199.9 ± 0.7	151.4 ± 2.2	209.4 ± 1.9	137.1 ± 1.3	299.5 ± 3.0	607 ± 6
262.75	204.1 ± 0.7	156.1 ± 2.2	213.0 ± 1.9	141.4 ± 1.3	308.4 ± 3.0	622 ± 6
263.0	208.4 ± 0.6	161.0 ± 2.1	216.6 ± 1.8	146.0 ± 1.3	317.6 ± 3.0	637 ± 6
263.25	212.7 ± 0.6	166.0 ± 2.1	220.3 ± 1.8	150.6 ± 1.3	327.1 ± 3.0	652 ± 6
263.5	217.2 ± 0.6	171.1 ± 2.1	224.1 ± 1.8	155.4 ± 1.2	336.8 ± 2.9	668 ± 6
263.75	221.7 ± 0.6	176.4 ± 2.1	227.9 ± 1.7	160.3 ± 1.2	346.8 ± 2.9	684 ± 6
264.0	226.3 ± 0.6	181.9 ± 2.0	231.8 ± 1.7	165.4 ± 1.2	357.1 ± 2.9	700 ± 6
264.25	231.0 ± 0.6	187.5 ± 2.0	235.7 ± 1.7	170.6 ± 1.2	367.7 ± 2.8	717 ± 6
264.5	235.8 ± 0.6	193.2 ± 2.0	239.7 ± 1.6	176.0 ± 1.1	378.5 ± 2.8	734 ± 6
264.75	240.7 ± 0.5	199.2 ± 1.9	243.7 ± 1.6	181.6 ± 1.1	389.7 ± 2.8	751 ± 6
265.0	245.7 ± 0.5	205.3 ± 1.9	247.8 ± 1.6	187.3 ± 1.1	401.1 ± 2.7	769 ± 6
265.25	250.7 ± 0.5	211.6 ± 1.8	252.0 ± 1.5	193.1 ± 1.1	412.9 ± 2.7	787 ± 6
265.5	255.9 ± 0.5	218.0 ± 1.8	256.2 ± 1.5	199.2 ± 1.0	425.0 ± 2.6	806 ± 6
265.75	261.2 ± 0.5	224.7 ± 1.7	260.5 ± 1.5	205.4 ± 1.0	437.4 ± 2.5	824 ± 6
266.0	266.5 ± 0.5	231.5 ± 1.7	264.9 ± 1.4	211.8 ± 0.9	450.2 ± 2.5	844 ± 5
266.25	272.0 ± 0.4	238.6 ± 1.6	269.3 ± 1.4	218.4 ± 0.9	463.3 ± 2.4	864 ± 5
266.5	277.5 ± 0.4	245.8 ± 1.6	273.8 ± 1.3	225.2 ± 0.9	476.8 ± 2.4	884 ± 5
266.75	283.2 ± 0.4	253.2 ± 1.5	278.3 ± 1.3	232.2 ± 0.8	490.6 ± 2.3	904 ± 5
267.0	289.0 ± 0.4	260.9 ± 1.5	282.9 ± 1.3	239.4 ± 0.8	504.8 ± 2.2	925 ± 5
267.25	294.8 ± 0.4	268.8 ± 1.4	287.6 ± 1.2	246.8 ± 0.7	519.4 ± 2.2	947 ± 5
267.5	300.8 ± 0.4	276.8 ± 1.3	292.4 ± 1.2	254.4 ± 0.7	534.4 ± 2.1	969 ± 5
267.75	306.89 ± 0.35	285.2 ± 1.3	297.2 ± 1.1	262.2 ± 0.7	549.8 ± 2.0	991 ± 5
268.0	313.09 ± 0.34	293.7 ± 1.3	302.1 ± 1.1	270.3 ± 0.6	565.6 ± 2.0	1014 ± 5
268.25	319.40 ± 0.33	302.5 ± 1.2	307.1 ± 1.1	278.6 ± 0.6	581.9 ± 1.9	1038 ± 4
268.5	325.83 ± 0.32	311.5 ± 1.2	312.1 ± 1.0	287.1 ± 0.6	598.5 ± 1.9	1062 ± 4
268.75	332.38 ± 0.32	320.8 ± 1.2	317.2 ± 1.0	295.9 ± 0.6	615.6 ± 1.9	1086 ± 4
269.0	339.04 ± 0.32	330.4 ± 1.3	322.4 ± 1.0	304.9 ± 0.7	633.2 ± 1.9	1111 ± 4
269.25	345.83 ± 0.33	340.2 ± 1.3	327.7 ± 1.0	314.2 ± 0.7	651.2 ± 2.0	1136 ± 4
269.5	352.74 ± 0.34	350.3 ± 1.4	333.0 ± 1.0	323.7 ± 0.8	669.8 ± 2.0	1162 ± 4
269.75	359.8 ± 0.4	360.7 ± 1.5	338.5 ± 1.0	333.5 ± 0.9	688.8 ± 2.1	1189 ± 4
270.0	366.9 ± 0.4	371.3 ± 1.7	344.0 ± 1.0	343.6 ± 1.0	708.3 ± 2.2	1216 ± 4
270.25	374.2 ± 0.4	382.3 ± 1.8	349.5 ± 1.0	354.0 ± 1.1	728.3 ± 2.4	1244 ± 4
270.5	381.7 ± 0.4	393.5 ± 2.0	355.2 ± 1.0	364.7 ± 1.3	748.9 ± 2.5	1272 ± 4
270.75	389.2 ± 0.5	405.1 ± 2.2	360.9 ± 1.0	375.7 ± 1.4	770.0 ± 2.7	1301 ± 4
271.0	396.9 ± 0.5	416.9 ± 2.4	366.7 ± 1.1	387.0 ± 1.6	791.6 ± 3.0	1330 ± 4
271.25	404.7 ± 0.6	429.2 ± 2.7	372.6 ± 1.1	398.6 ± 1.7	813.8 ± 3.2	1360 ± 4
271.5	412.7 ± 0.6	441.7 ± 2.9	378.6 ± 1.2	410.5 ± 1.9	836.7 ± 3.5	1391 ± 5
271.75	420.8 ± 0.7	454.6 ± 3.2	384.7 ± 1.2	422.8 ± 2.1	860 ± 4	1422 ± 5
272.0	429.1 ± 0.7	467.8 ± 3.5	390.9 ± 1.3	435.4 ± 2.3	884 ± 4	1454 ± 5
272.25	437.5 ± 0.8	481 ± 4	397.1 ± 1.4	448.3 ± 2.5	909 ± 4	1487 ± 5
272.5	446.0 ± 0.8	495 ± 4	403.4 ± 1.5	461.7 ± 2.8	934 ± 5	1520 ± 6
272.75	454.7 ± 0.9	510 ± 5	409.9 ± 1.6	475.4 ± 3.0	960 ± 5	1554 ± 6
273.0	463.6 ± 0.9	524 ± 5	416.4 ± 1.7	489.4 ± 3.2	986 ± 6	1588 ± 6
273.25	472.6 ± 1.0	540 ± 5	423.0 ± 1.8	503.9 ± 3.5	1014 ± 6	1624 ± 7
273.5	481.8 ± 1.1	555 ± 6	429.7 ± 1.9	519 ± 4	1042 ± 7	1660 ± 7
273.75	491.1 ± 1.2	571 ± 6	436.5 ± 2.0	534 ± 4	1070 ± 7	1697 ± 8
274.0	500.6 ± 1.2	587 ± 7	443.4 ± 2.2	550 ± 4	1100 ± 8	1734 ± 9
274.25	510.3 ± 1.3	604 ± 7	450.4 ± 2.3	566 ± 5	1130 ± 8	1772 ± 9
274.5	520.1 ± 1.4	622 ± 8	457.4 ± 2.4	582 ± 5	1161 ± 9	1811 ± 10
274.75	530.1 ± 1.5	639 ± 8	464.6 ± 2.6	599 ± 5	1193 ± 9	1851 ± 10
275.0	540.3 ± 1.6	657 ± 9	471.9 ± 2.7	617 ± 6	1225 ± 10	1892 ± 11
275.25	550.7 ± 1.6	676 ± 9	479.3 ± 2.9	635 ± 6	1259 ± 11	1933 ± 12
275.5	561.2 ± 1.7	695 ± 10	486.8 ± 3.0	653 ± 7	1293 ± 11	1976 ± 13
275.75	571.9 ± 1.8	715 ± 10	494.3 ± 3.2	672 ± 7	1328 ± 12	2019 ± 13
276.0	582.8 ± 1.9	735 ± 11	502.0 ± 3.3	692 ± 7	1364 ± 13	2063 ± 14
276.25	593.9 ± 2.0	756 ± 12	509.8 ± 3.5	712 ± 8	1401 ± 14	2108 ± 15
276.5	605.2 ± 2.1	777 ± 12	518 ± 4	732 ± 8	1439 ± 14	2154 ± 16
276.75	616.7 ± 2.2	799 ± 13	526 ± 4	754 ± 9	1478 ± 15	2200 ± 17
277.0	628.3 ± 2.3	822 ± 14	534 ± 4	775 ± 9	1518 ± 16	2248 ± 18
277.25	640.2 ± 2.5	845 ± 15	542 ± 4	797 ± 10	1558 ± 17	2297 ± 19
277.5	652.3 ± 2.6	868 ± 16	550 ± 4	820 ± 10	1600 ± 18	2346 ± 20
277.75	664.6 ± 2.7	892 ± 16	559 ± 5	844 ± 11	1643 ± 19	2397 ± 21
278.0	677.1 ± 2.8	917 ± 17	567 ± 5	868 ± 12	1687 ± 20	2448 ± 22
278.25	689.8 ± 2.9	943 ± 18	576 ± 5	893 ± 12	1732 ± 21	2501 ± 23
278.5	702.7 ± 3.1	969 ± 19	585 ± 5	918 ± 13	1778 ± 22	2554 ± 24
278.75	715.8 ± 3.2	996 ± 20	594 ± 6	944 ± 14	1825 ± 23	2609 ± 25
279.0	729.2 ± 3.4	1023 ± 21	603 ± 6	971 ± 14	1874 ± 25	2665 ± 26
279.25	742.8 ± 3.5	1052 ± 22	612 ± 6	998 ± 15	1924 ± 26	2721 ± 28
279.5	757 ± 4	1081 ± 24	621 ± 6	1027 ± 16	1974 ± 27	2779 ± 29
279.75	771 ± 4	1111 ± 25	631 ± 6	1056 ± 17	2026 ± 28	2838 ± 30
280.0	785 ± 4	1141 ± 26	640 ± 7	1085 ± 17	2080 ± 30	2899 ± 32
280.25	799 ± 4	1172 ± 27	650 ± 7	1116 ± 18	2135 ± 31	2960 ± 33
280.5	814 ± 4	1204 ± 29	660 ± 7	1147 ± 19	2191 ± 33	3022 ± 35
280.75	829 ± 4	1237 ± 30	670 ± 8	1179 ± 20	2248 ± 34	$(3.09 \pm 0.04) \times 10^3$
281.0	844 ± 5	1271 ± 31	680 ± 8	1212 ± 21	$(2.31 \pm 0.04) \times 10^3$	$(3.15 \pm 0.04) \times 10^3$
281.25	860 ± 5	1306 ± 33	690 ± 8	1246 ± 22	$(2.37 \pm 0.04) \times 10^3$	$(3.22 \pm 0.04) \times 10^3$
281.5	876 ± 5	1341 ± 34	700 ± 8	1281 ± 23	$(2.43 \pm 0.04) \times 10^3$	$(3.28 \pm 0.04) \times 10^3$
281.75	892 ± 5	$(1.38 \pm 0.04) \times 10^3$	711 ± 9	1316 ± 24	$(2.49 \pm 0.04) \times 10^3$	$(3.35 \pm 0.04) \times 10^3$
282.0	908 ± 5	$(1.41 \pm 0.04) \times 10^3$	721 ± 9	1353 ± 25	$(2.56 \pm 0.04) \times 10^3$	$(3.42 \pm 0.04) \times 10^3$
282.25	925 ± 6	$(1.45 \pm 0.04) \times 10^3$	732 ± 9	1390 ± 26	$(2.62 \pm 0.04) \times 10^3$	$(3.50 \pm 0.05) \times 10^3$
282.5	941 ± 6	$(1.49 \pm 0.04) \times 10^3$	743 ± 10	1429 ± 28	$(2.69 \pm 0.05) \times 10^3$	$(3.57 \pm 0.05) \times 10^3$
282.75	959 ± 6	$(1.53 \pm 0.04) \times 10^3$	754 ± 10	1468 ± 29	$(2.76 \pm 0.05) \times 10^3$	$(3.64 \pm 0.05) \times 10^3$
283.0	976 ± 6	$(1.57 \pm 0.04) \times 10^3$	765 ± 10	1509 ± 30	$(2.83 \pm 0.05) \times 10^3$	$(3.72 \pm 0.05) \times 10^3$
283.25	994 ± 6	$(1.62 \pm 0.05) \times 10^3$	777 ± 11	1550 ± 32	$(2.90 \pm 0.05) \times 10^3$	$(3.79 \pm 0.05) \times 10^3$
283.5	1012 ± 7	$(1.66 \pm 0.05) \times 10^3$	788 ± 11	1593 ± 33	$(2.98 \pm 0.06) \times 10^3$	$(3.87 \pm 0.06) \times 10^3$
283.75	1030 ± 7	$(1.70 \pm 0.05) \times 10^3$	800 ± 11	1636 ± 34	$(3.06 \pm 0.06) \times 10^3$	$(3.95 \pm 0.06) \times 10^3$
284.0	1048 ± 7	$(1.75 \pm 0.05) \times 10^3$	811 ± 12	$(1.68 \pm 0.04) \times 10^3$	$(3.13 \pm 0.06) \times 10^3$	$(4.03 \pm 0.06) \times 10^3$
284.25	1067 ± 7	$(1.80 \pm 0.06) \times 10^3$	823 ± 12	$(1.73 \pm 0.04) \times 10^3$	$(3.21 \pm 0.06) \times 10^3$	$(4.12 \pm 0.06) \times 10^3$
284.5	1086 ± 8	$(1.84 \pm 0.06) \times 10^3$	835 ± 12	$(1.77 \pm 0.04) \times 10^3$	$(3.30 \pm 0.07) \times 10^3$	$(4.20 \pm 0.07) \times 10^3$
284.75	1106 ± 8	$(1.89 \pm 0.06) \times 10^3$	848 ± 13	$(1.82 \pm 0.04) \times 10^3$	$(3.38 \pm 0.07) \times 10^3$	$(4.29 \pm 0.07) \times 10^3$
285.0	1126 ± 8	$(1.94 \pm 0.06) \times 10^3$	860 ± 13	$(1.87 \pm 0.04) \times 10^3$	$(3.47 \pm 0.07) \times 10^3$	$(4.38 \pm 0.07) \times 10^3$

Table 6: CONT: Exchange rates extrapolated to 310 K, 1M glucose at pH=7.38, using restricted model, with partial assignment.

T(K)	C_6	$C_{3/4}$	$C_{3/4}$	C_2	$C_{1,\alpha}$	$C_{1,\beta}$
285.25	1146 ± 8	(1.99 ± 0.07) × 10^3	873 ± 14	(1.92 ± 0.04) × 10^3	(3.55 ± 0.07) × 10^3	(4.47 ± 0.07) × 10^3
285.5	1166 ± 9	(2.05 ± 0.07) × 10^3	885 ± 14	(1.97 ± 0.05) × 10^3	(3.64 ± 0.08) × 10^3	(4.56 ± 0.08) × 10^3
285.75	1187 ± 9	(2.10 ± 0.07) × 10^3	898 ± 14	(2.03 ± 0.05) × 10^3	(3.74 ± 0.08) × 10^3	(4.65 ± 0.08) × 10^3
286.0	1208 ± 9	(2.16 ± 0.07) × 10^3	911 ± 15	(2.08 ± 0.05) × 10^3	(3.83 ± 0.08) × 10^3	(4.74 ± 0.08) × 10^3
286.25	1230 ± 9	(2.21 ± 0.08) × 10^3	924 ± 15	(2.14 ± 0.05) × 10^3	(3.93 ± 0.09) × 10^3	(4.84 ± 0.08) × 10^3
286.5	1251 ± 10	(2.27 ± 0.08) × 10^3	938 ± 16	(2.20 ± 0.05) × 10^3	(4.03 ± 0.09) × 10^3	(4.94 ± 0.09) × 10^3
286.75	1273 ± 10	(2.33 ± 0.08) × 10^3	951 ± 16	(2.26 ± 0.06) × 10^3	(4.13 ± 0.09) × 10^3	(5.04 ± 0.09) × 10^3
287.0	1296 ± 10	(2.39 ± 0.09) × 10^3	965 ± 17	(2.32 ± 0.06) × 10^3	(4.23 ± 0.10) × 10^3	(5.14 ± 0.09) × 10^3
287.25	1319 ± 11	(2.45 ± 0.09) × 10^3	979 ± 17	(2.38 ± 0.06) × 10^3	(4.34 ± 0.10) × 10^3	(5.24 ± 0.10) × 10^3
287.5	1342 ± 11	(2.52 ± 0.09) × 10^3	993 ± 18	(2.44 ± 0.06) × 10^3	(4.45 ± 0.10) × 10^3	(5.35 ± 0.10) × 10^3
287.75	1366 ± 11	(2.58 ± 0.10) × 10^3	1007 ± 18	(2.51 ± 0.07) × 10^3	(4.56 ± 0.11) × 10^3	(5.46 ± 0.10) × 10^3
288.0	1389 ± 12	(2.65 ± 0.10) × 10^3	1022 ± 18	(2.57 ± 0.07) × 10^3	(4.67 ± 0.11) × 10^3	(5.57 ± 0.11) × 10^3
288.25	1414 ± 12	(2.72 ± 0.10) × 10^3	1036 ± 19	(2.64 ± 0.07) × 10^3	(4.79 ± 0.12) × 10^3	(5.68 ± 0.11) × 10^3
288.5	1439 ± 12	(2.79 ± 0.11) × 10^3	1051 ± 20	(2.71 ± 0.07) × 10^3	(4.90 ± 0.12) × 10^3	(5.79 ± 0.12) × 10^3
288.75	1464 ± 13	(2.86 ± 0.11) × 10^3	1066 ± 20	(2.78 ± 0.08) × 10^3	(5.03 ± 0.13) × 10^3	(5.91 ± 0.12) × 10^3
289.0	1489 ± 13	(2.94 ± 0.12) × 10^3	1081 ± 21	(2.86 ± 0.08) × 10^3	(5.15 ± 0.13) × 10^3	(6.02 ± 0.12) × 10^3
289.25	1515 ± 13	(3.01 ± 0.12) × 10^3	1096 ± 21	(2.93 ± 0.08) × 10^3	(5.28 ± 0.13) × 10^3	(6.14 ± 0.13) × 10^3
289.5	1541 ± 14	(3.09 ± 0.13) × 10^3	1112 ± 22	(3.01 ± 0.09) × 10^3	(5.41 ± 0.14) × 10^3	(6.27 ± 0.13) × 10^3
289.75	1568 ± 14	(3.17 ± 0.13) × 10^3	1128 ± 22	(3.09 ± 0.09) × 10^3	(5.54 ± 0.14) × 10^3	(6.39 ± 0.14) × 10^3
290.0	1595 ± 15	(3.25 ± 0.13) × 10^3	1144 ± 23	(3.17 ± 0.09) × 10^3	(5.68 ± 0.15) × 10^3	(6.52 ± 0.14) × 10^3
290.25	1623 ± 15	(3.33 ± 0.14) × 10^3	1160 ± 23	(3.25 ± 0.10) × 10^3	(5.82 ± 0.16) × 10^3	(6.64 ± 0.14) × 10^3
290.5	1651 ± 15	(3.42 ± 0.14) × 10^3	1176 ± 24	(3.34 ± 0.10) × 10^3	(5.96 ± 0.16) × 10^3	(6.78 ± 0.15) × 10^3
290.75	1679 ± 16	(3.50 ± 0.15) × 10^3	1193 ± 25	(3.43 ± 0.10) × 10^3	(6.10 ± 0.17) × 10^3	(6.91 ± 0.15) × 10^3
291.0	1708 ± 16	(3.59 ± 0.16) × 10^3	1209 ± 25	(3.51 ± 0.11) × 10^3	(6.25 ± 0.17) × 10^3	(7.04 ± 0.16) × 10^3
291.25	1737 ± 17	(3.68 ± 0.16) × 10^3	1226 ± 26	(3.61 ± 0.11) × 10^3	(6.41 ± 0.18) × 10^3	(7.18 ± 0.16) × 10^3
291.5	1767 ± 17	(3.78 ± 0.17) × 10^3	1243 ± 27	(3.70 ± 0.11) × 10^3	(6.56 ± 0.18) × 10^3	(7.32 ± 0.17) × 10^3
291.75	1797 ± 18	(3.87 ± 0.17) × 10^3	1261 ± 27	(3.80 ± 0.12) × 10^3	(6.72 ± 0.19) × 10^3	(7.47 ± 0.17) × 10^3
292.0	1828 ± 18	(3.97 ± 0.18) × 10^3	1278 ± 28	(3.89 ± 0.12) × 10^3	(6.88 ± 0.20) × 10^3	(7.61 ± 0.18) × 10^3
292.25	1859 ± 19	(4.07 ± 0.19) × 10^3	1296 ± 29	(3.99 ± 0.13) × 10^3	(7.05 ± 0.20) × 10^3	(7.76 ± 0.18) × 10^3
292.5	1890 ± 19	(4.17 ± 0.19) × 10^3	1314 ± 29	(4.10 ± 0.13) × 10^3	(7.22 ± 0.21) × 10^3	(7.91 ± 0.19) × 10^3
292.75	1923 ± 20	(4.28 ± 0.20) × 10^3	1332 ± 30	(4.20 ± 0.14) × 10^3	(7.39 ± 0.22) × 10^3	(8.06 ± 0.19) × 10^3
293.0	1955 ± 20	(4.39 ± 0.21) × 10^3	1351 ± 31	(4.31 ± 0.14) × 10^3	(7.57 ± 0.23) × 10^3	(8.22 ± 0.20) × 10^3
293.25	1988 ± 21	(4.50 ± 0.21) × 10^3	1369 ± 31	(4.42 ± 0.15) × 10^3	(7.75 ± 0.23) × 10^3	(8.38 ± 0.21) × 10^3
293.5	2022 ± 21	(4.61 ± 0.22) × 10^3	1388 ± 32	(4.54 ± 0.15) × 10^3	(7.94 ± 0.24) × 10^3	(8.54 ± 0.21) × 10^3
293.75	2056 ± 22	(4.72 ± 0.23) × 10^3	1407 ± 33	(4.65 ± 0.16) × 10^3	(8.13 ± 0.25) × 10^3	(8.71 ± 0.22) × 10^3
294.0	2091 ± 23	(4.84 ± 0.23) × 10^3	1427 ± 34	(4.77 ± 0.16) × 10^3	(8.33 ± 0.26) × 10^3	(8.87 ± 0.23) × 10^3
294.25	2126 ± 23	(4.96 ± 0.24) × 10^3	1446 ± 34	(4.89 ± 0.17) × 10^3	(8.52 ± 0.27) × 10^3	(9.04 ± 0.23) × 10^3
294.5	2161 ± 24	(5.09 ± 0.25) × 10^3	1466 ± 35	(5.02 ± 0.17) × 10^3	(8.73 ± 0.28) × 10^3	(9.22 ± 0.24) × 10^3
294.75	2198 ± 24	(5.21 ± 0.26) × 10^3	(1.49 ± 0.04) × 10^3	(5.14 ± 0.18) × 10^3	(8.93 ± 0.29) × 10^3	(9.39 ± 0.25) × 10^3
295.0	2234 ± 25	(5.34 ± 0.27) × 10^3	(1.51 ± 0.04) × 10^3	(5.27 ± 0.19) × 10^3	(9.15 ± 0.29) × 10^3	(9.57 ± 0.25) × 10^3
295.25	2272 ± 26	(5.47 ± 0.28) × 10^3	(1.53 ± 0.04) × 10^3	(5.41 ± 0.19) × 10^3	(9.36 ± 0.30) × 10^3	(9.76 ± 0.26) × 10^3
295.5	2309 ± 26	(5.61 ± 0.29) × 10^3	(1.55 ± 0.04) × 10^3	(5.54 ± 0.20) × 10^3	(9.59 ± 0.31) × 10^3	(9.94 ± 0.27) × 10^3
295.75	2348 ± 27	(5.75 ± 0.30) × 10^3	(1.57 ± 0.04) × 10^3	(5.68 ± 0.20) × 10^3	(9.81 ± 0.32) × 10^3	(1.013 ± 0.027) × 10^4
296.0	2387 ± 28	(5.89 ± 0.31) × 10^3	(1.59 ± 0.04) × 10^3	(5.83 ± 0.21) × 10^3	(1.004 ± 0.033) × 10^4	(1.032 ± 0.028) × 10^4
296.25	2426 ± 28	(6.03 ± 0.32) × 10^3	(1.61 ± 0.04) × 10^3	(5.97 ± 0.22) × 10^3	(1.028 ± 0.035) × 10^4	(1.052 ± 0.029) × 10^4
296.5	2467 ± 29	(6.18 ± 0.33) × 10^3	(1.63 ± 0.04) × 10^3	(6.12 ± 0.23) × 10^3	(1.05 ± 0.04) × 10^4	(1.072 ± 0.030) × 10^4
296.75	2507 ± 30	(6.33 ± 0.34) × 10^3	(1.66 ± 0.04) × 10^3	(6.28 ± 0.23) × 10^3	(1.08 ± 0.04) × 10^4	(1.092 ± 0.031) × 10^4
297.0	2549 ± 30	(6.49 ± 0.35) × 10^3	(1.68 ± 0.04) × 10^3	(6.44 ± 0.24) × 10^3	(1.10 ± 0.04) × 10^4	(1.113 ± 0.032) × 10^4
297.25	2591 ± 31	(6.6 ± 0.4) × 10^3	(1.70 ± 0.05) × 10^3	(6.60 ± 0.25) × 10^3	(1.13 ± 0.04) × 10^4	(1.134 ± 0.032) × 10^4
297.5	2633 ± 32	(6.8 ± 0.4) × 10^3	(1.72 ± 0.05) × 10^3	(6.76 ± 0.26) × 10^3	(1.15 ± 0.04) × 10^4	(1.155 ± 0.033) × 10^4
297.75	2677 ± 33	(7.0 ± 0.4) × 10^3	(1.75 ± 0.05) × 10^3	(6.93 ± 0.27) × 10^3	(1.18 ± 0.04) × 10^4	(1.177 ± 0.034) × 10^4
298.0	2720 ± 34	(7.1 ± 0.4) × 10^3	(1.77 ± 0.05) × 10^3	(7.10 ± 0.27) × 10^3	(1.21 ± 0.04) × 10^4	(1.199 ± 0.035) × 10^4
298.25	2765 ± 34	(7.3 ± 0.4) × 10^3	(1.79 ± 0.05) × 10^3	(7.28 ± 0.28) × 10^3	(1.24 ± 0.04) × 10^4	(1.22 ± 0.04) × 10^4
298.5	2810 ± 35	(7.5 ± 0.4) × 10^3	(1.82 ± 0.05) × 10^3	(7.46 ± 0.29) × 10^3	(1.27 ± 0.05) × 10^4	(1.24 ± 0.04) × 10^4
298.75	(2.86 ± 0.04) × 10^3	(7.7 ± 0.4) × 10^3	(1.84 ± 0.05) × 10^3	(7.64 ± 0.30) × 10^3	(1.29 ± 0.05) × 10^4	(1.27 ± 0.04) × 10^4
299.0	(2.90 ± 0.04) × 10^3	(7.9 ± 0.4) × 10^3	(1.87 ± 0.05) × 10^3	(7.83 ± 0.31) × 10^3	(1.32 ± 0.05) × 10^4	(1.29 ± 0.04) × 10^4
299.25	(2.95 ± 0.04) × 10^3	(8.0 ± 0.5) × 10^3	(1.89 ± 0.05) × 10^3	(8.02 ± 0.32) × 10^3	(1.36 ± 0.05) × 10^4	(1.31 ± 0.04) × 10^4
299.5	(3.00 ± 0.04) × 10^3	(8.2 ± 0.5) × 10^3	(1.92 ± 0.05) × 10^3	(8.22 ± 0.33) × 10^3	(1.39 ± 0.05) × 10^4	(1.34 ± 0.04) × 10^4
299.75	(3.05 ± 0.04) × 10^3	(8.4 ± 0.5) × 10^3	(1.94 ± 0.06) × 10^3	(8.42 ± 0.34) × 10^3	(1.42 ± 0.05) × 10^4	(1.36 ± 0.04) × 10^4
300.0	(3.10 ± 0.04) × 10^3	(8.6 ± 0.5) × 10^3	(1.97 ± 0.06) × 10^3	(8.63 ± 0.35) × 10^3	(1.45 ± 0.05) × 10^4	(1.39 ± 0.04) × 10^4
300.25	(3.15 ± 0.04) × 10^3	(8.8 ± 0.5) × 10^3	(1.99 ± 0.06) × 10^3	(8.8 ± 0.4) × 10^3	(1.48 ± 0.06) × 10^4	(1.41 ± 0.04) × 10^4
300.5	(3.20 ± 0.04) × 10^3	(9.1 ± 0.5) × 10^3	(2.02 ± 0.06) × 10^3	(9.1 ± 0.4) × 10^3	(1.52 ± 0.06) × 10^4	(1.44 ± 0.05) × 10^4
300.75	(3.25 ± 0.04) × 10^3	(9.3 ± 0.6) × 10^3	(2.05 ± 0.06) × 10^3	(9.3 ± 0.4) × 10^3	(1.55 ± 0.06) × 10^4	(1.47 ± 0.05) × 10^4
301.0	(3.30 ± 0.04) × 10^3	(9.5 ± 0.6) × 10^3	(2.07 ± 0.06) × 10^3	(9.5 ± 0.4) × 10^3	(1.59 ± 0.06) × 10^4	(1.49 ± 0.05) × 10^4
301.25	(3.35 ± 0.05) × 10^3	(9.7 ± 0.6) × 10^3	(2.10 ± 0.06) × 10^3	(9.7 ± 0.4) × 10^3	(1.62 ± 0.06) × 10^4	(1.52 ± 0.05) × 10^4
301.5	(3.41 ± 0.05) × 10^3	(9.9 ± 0.6) × 10^3	(2.13 ± 0.06) × 10^3	(1.00 ± 0.04) × 10^4	(1.66 ± 0.07) × 10^4	(1.55 ± 0.05) × 10^4
301.75	(3.46 ± 0.05) × 10^3	(1.02 ± 0.06) × 10^4	(2.16 ± 0.07) × 10^3	(1.02 ± 0.04) × 10^4	(1.70 ± 0.07) × 10^4	(1.58 ± 0.05) × 10^4
302.0	(3.52 ± 0.05) × 10^3	(1.04 ± 0.06) × 10^4	(2.18 ± 0.07) × 10^3	(1.05 ± 0.05) × 10^4	(1.74 ± 0.07) × 10^4	(1.61 ± 0.05) × 10^4
302.25	(3.57 ± 0.05) × 10^3	(1.07 ± 0.07) × 10^4	(2.21 ± 0.07) × 10^3	(1.07 ± 0.05) × 10^4	(1.78 ± 0.07) × 10^4	(1.64 ± 0.05) × 10^4
302.5	(3.63 ± 0.05) × 10^3	(1.09 ± 0.07) × 10^4	(2.24 ± 0.07) × 10^3	(1.10 ± 0.05) × 10^4	(1.82 ± 0.07) × 10^4	(1.67 ± 0.06) × 10^4
302.75	(3.69 ± 0.05) × 10^3	(1.12 ± 0.07) × 10^4	(2.27 ± 0.07) × 10^3	(1.12 ± 0.05) × 10^4	(1.86 ± 0.08) × 10^4	(1.70 ± 0.06) × 10^4
303.0	(3.75 ± 0.05) × 10^3	(1.14 ± 0.07) × 10^4	(2.30 ± 0.07) × 10^3	(1.15 ± 0.05) × 10^4	(1.90 ± 0.08) × 10^4	(1.73 ± 0.06) × 10^4
303.25	(3.80 ± 0.05) × 10^3	(1.17 ± 0.08) × 10^4	(2.33 ± 0.07) × 10^3	(1.18 ± 0.05) × 10^4	(1.94 ± 0.08) × 10^4	(1.76 ± 0.06) × 10^4
303.5	(3.86 ± 0.06) × 10^3	(1.20 ± 0.08) × 10^4	(2.36 ± 0.08) × 10^3	(1.21 ± 0.05) × 10^4	(1.99 ± 0.08) × 10^4	(1.79 ± 0.06) × 10^4
303.75	(3.93 ± 0.06) × 10^3	(1.23 ± 0.08) × 10^4	(2.39 ± 0.08) × 10^3	(1.24 ± 0.06) × 10^4	(2.03 ± 0.09) × 10^4	(1.82 ± 0.06) × 10^4
304.0	(3.99 ± 0.06) × 10^3	(1.25 ± 0.08) × 10^4	(2.42 ± 0.08) × 10^3	(1.27 ± 0.06) × 10^4	(2.08 ± 0.09) × 10^4	(1.86 ± 0.06) × 10^4
304.25	(4.05 ± 0.06) × 10^3	(1.28 ± 0.08) × 10^4	(2.45 ± 0.08) × 10^3	(1.30 ± 0.06) × 10^4	(2.12 ± 0.09) × 10^4	(1.89 ± 0.07) × 10^4
304.5	(4.11 ± 0.06) × 10^3	(1.31 ± 0.09) × 10^4	(2.48 ± 0.08) × 10^3	(1.33 ± 0.06) × 10^4	(2.17 ± 0.09) × 10^4	(1.92 ± 0.07) × 10^4
304.75	(4.18 ± 0.06) × 10^3	(1.34 ± 0.09) × 10^4	(2.51 ± 0.08) × 10^3	(1.36 ± 0.06) × 10^4	(2.22 ± 0.10) × 10^4	(1.96 ± 0.07) × 10^4
305.0	(4.24 ± 0.06) × 10^3	(1.38 ± 0.09) × 10^4	(2.55 ± 0.08) × 10^3	(1.39 ± 0.07) × 10^4	(2.27 ± 0.10) × 10^4	(1.99 ± 0.07) × 10^4
305.25	(4.31 ± 0.06) × 10^3	(1.41 ± 0.10) × 10^4	(2.58 ± 0.09) × 10^3	(1.42 ± 0.07) × 10^4	(2.32 ± 0.10) × 10^4	(2.03 ± 0.07) × 10^4
305.5	(4.38 ± 0.07) × 10^3	(1.44 ± 0.10) × 10^4	(2.61 ± 0.09) × 10^3	(1.46 ± 0.07) × 10^4	(2.37 ± 0.10) × 10^4	(2.06 ± 0.08) × 10^4
305.75	(4.45 ± 0.07) × 10^3	(1.47 ± 0.10) × 10^4	(2.65 ± 0.09) × 10^3	(1.49 ± 0.07) × 10^4	(2.42 ± 0.11) × 10^4	(2.10 ± 0.08) × 10^4
306.0	(4.52 ± 0.07) × 10^3	(1.51 ± 0.10) × 10^4	(2.68 ± 0.09) × 10^3	(1.53 ± 0.07) × 10^4	(2.48 ± 0.11) × 10^4	(2.14 ± 0.08) × 10^4
306.25	(4.59 ± 0.07) × 10^3	(1.54 ± 0.11) × 10^4	(2.71 ± 0.09) × 10^3	(1.56 ± 0.08) × 10^4	(2.53 ± 0.11) × 10^4	(2.18 ± 0.08) × 10^4
306.5	(4.66 ± 0.07) × 10^3	(1.58 ± 0.11) × 10^4	(2.75 ± 0.09) × 10^3	(1.60 ± 0.08) × 10^4	(2.59 ± 0.12) × 10^4	(2.21 ± 0.08) × 10^4
306.75	(4.73 ± 0.07) × 10^3	(1.61 ± 0.11) × 10^4	(2.78 ± 0.10) × 10^3	(1.64 ± 0.08) × 10^4	(2.64 ± 0.12) × 10^4	(2.25 ± 0.08) × 10^4
307.0	(4.80 ± 0.08) × 10^3	(1.65 ± 0.12) × 10^4	(2.82 ± 0.10) × 10^3	(1.67 ± 0.08) × 10^4	(2.70 ± 0.12) × 10^4	(2.29 ± 0.09) × 10^4
307.25	(4.88 ± 0.08) × 10^3	(1.69 ± 0.12) × 10^4	(2.85 ± 0.10) × 10^3	(1.71 ± 0.08) × 10^4	(2.76 ± 0.13) × 10^4	(2.33 ± 0.09) × 10^4
307.5	(4.95 ± 0.08) × 10^3	(1.73 ± 0.12) × 10^4	(2.89 ± 0.10) × 10^3	(1.75 ± 0.09) × 10^4	(2.82 ± 0.13) × 10^4	(2.38 ± 0.09) × 10^4
307.75	(5.03 ± 0.08) × 10^3	(1.77 ± 0.13) × 10^4	(2.93 ± 0.10) × 10^3	(1.79 ± 0.09) × 10^4	(2.88 ± 0.13) × 10^4	(2.42 ± 0.09) × 10^4
308.0	(5.10 ± 0.08) × 10^3	(1.81 ± 0.13) × 10^4	(2.96 ± 0.11) × 10^3	(1.84 ± 0.09) × 10^4	(2.94 ± 0.14) × 10^4	(2.46 ± 0.10) × 10^4
308.25	(5.18 ± 0.08) × 10^3	(1.85 ± 0.13) × 10^4	(3.00 ± 0.11) × 10^3	(1.88 ± 0.09) × 10^4	(3.01 ± 0.14) × 10^4	(2.50 ± 0.10) × 10^4
308.5	(5.26 ± 0.09) × 10^3	(1.89 ± 0.14) × 10^4	(3.04 ± 0.11) × 10^3	(1.92 ± 0.10) × 10^4	(3.07 ± 0.14) × 10^4	(2.55 ± 0.10) × 10^4
308.75	(5.34 ± 0.09) × 10^3	(1.93 ± 0.14) × 10^4	(3.07 ± 0.11) × 10^3	(1.97 ± 0.10) × 10^4	(3.14 ± 0.15) × 10^4	(2.59 ± 0.10) × 10^4
309.0	(5.42 ± 0.09) × 10^3	(1.97 ± 0.15) × 10^4	(3.11 ± 0.11) × 10^3	(2.01 ± 0.10) × 10^4	(3.21 ± 0.15) × 10^4	(2.64 ± 0.10) × 10^4
309.25	(5.51 ± 0.09) × 10^3	(2.02 ± 0.15) × 10^4	(3.15 ± 0.12) × 10^3	(2.06 ± 0.11) × 10^4	(3.28 ± 0.16) × 10^4	(2.68 ± 0.11) × 10^4
309.5	(5.59 ± 0.09) × 10^3	(2.06 ± 0.15) × 10^4	(3.19 ± 0.12) × 10^3	(2.11 ± 0.11) × 10^4	(3.35 ± 0.16) × 10^4	(2.73 ± 0.11) × 10^4
309.75	(5.68 ± 0.09) × 10^3	(2.11 ± 0.16) × 10^4	(3.23 ± 0.12) × 10^3	(2.16 ± 0.11) × 10^4	(3.42 ± 0.16) × 10^4	(2.78 ± 0.11) × 10^4
310.0	(5.76 ± 0.10) × 10^3	(2.16 ± 0.16) × 10^4	(3.27 ± 0.12) × 10^3	(2.21 ± 0.12) × 10^4	(3.49 ± 0.17) × 10^4	(2.82 ± 0.11) × 10^4

Table 7: Chem shifts, 1M glucose at pH=6.21, using restricted model, with partial assignment. Signals 1 to 5

T(K)	C_6	$C_{3/4\alpha}$	$C_{3/4\beta}$	$C_{3/4\alpha}$	$C_{3/4\beta}$
269.27	0.78885 ± 0.00011	1.2290 ± 0.0011	1.37237 ± 0.00024	1.2198 ± 0.0005	1.43349 ± 0.00015
274.23	0.78644 ± 0.00015	1.2411 ± 0.0018	1.3686 ± 0.0008	1.2199 ± 0.0005	1.4326 ± 0.0005
279.19	0.78655 ± 0.00023	1.2499 ± 0.0008	1.3615 ± 0.0030	1.22000 ± 0.00023	1.4299 ± 0.0014
284.15	0.7786 ± 0.0004	1.2497 ± 0.0028	1.316 ± 0.006	1.2194 ± 0.0015	1.4487 ± 0.0024
289.11	0.7672 ± 0.0009	1.248 ± 0.004	1.313 ± 0.010	1.2199 ± 0.0031	1.434 ± 0.004
294.06	0.7603 ± 0.0021	1.244 ± 0.016	1.331 ± 0.007	1.204 ± 0.008	1.422 ± 0.004

Table 8: Chem shifts, 1M glucose at pH=6.21, using restricted model, with partial assignment. Signals 6 to 9

T(K)	$C_{2,\alpha}$	$C_{2,\beta}$	$C_{1,\alpha}$	$C_{1,\beta}$
269.27	1.07148 ± 0.00012	1.25909 ± 0.00028	2.06430 ± 0.00004	2.81704 ± 0.00009
274.23	1.06993 ± 0.00022	1.2601 ± 0.0007	2.07862 ± 0.00008	2.82553 ± 0.00014
279.19	1.0667 ± 0.0006	1.2597 ± 0.0007	2.09270 ± 0.00016	2.83369 ± 0.00024
284.15	1.0639 ± 0.0012	1.2686 ± 0.0020	2.10148 ± 0.00029	2.8372 ± 0.0004
289.11	1.0576 ± 0.0021	1.265 ± 0.004	2.1105 ± 0.0006	2.8386 ± 0.0007
294.06	1.049 ± 0.005	1.257 ± 0.006	2.1169 ± 0.0010	2.8351 ± 0.0012

Table 9: Chem shifts, 1M glucose at pH=7.00, using restricted model, with partial assignment. Signals 1 to 5

T(K)	C_6	$C_{3/4\alpha}$	$C_{3/4\beta}$	$C_{3/4\alpha}$	$C_{3/4\beta}$
267.88	0.77826 ± 0.00006	1.2139 ± 0.0010	1.35360 ± 0.00027	1.2139 ± 0.0010	1.43178 ± 0.00020
268.92	0.77559 ± 0.00007	1.2138 ± 0.0009	1.35343 ± 0.00035	1.2127 ± 0.0008	1.42465 ± 0.00027
271.53	0.77860 ± 0.00008	1.2347 ± 0.0013	1.3416 ± 0.0023	1.21999 ± 0.00018	1.4290 ± 0.0009
274.13	0.77544 ± 0.00012	1.2373 ± 0.0008	1.30002 ± 0.00010	1.21998 ± 0.00012	1.4377 ± 0.0005
276.73	0.77576 ± 0.00018	1.2361 ± 0.0016	1.3004 ± 0.0011	1.2200 ± 0.0005	1.4371 ± 0.0009

Table 10: Chem shifts, 1M glucose at pH=7.00, using restricted model, with partial assignment. Signals 6 to 9

T(K)	$C_{2,\alpha}$	$C_{2,\beta}$	$C_{1,\alpha}$	$C_{1,\beta}$
267.88	1.06316 ± 0.00011	1.24909 ± 0.00016	2.05641 ± 0.00020	2.81210 ± 0.00030
268.92	1.06199 ± 0.00013	1.24609 ± 0.00025	2.05779 ± 0.00025	2.80938 ± 0.00035
271.53	1.06047 ± 0.00017	1.2331 ± 0.0010	2.06362 ± 0.00033	2.8108 ± 0.0005
274.13	1.05868 ± 0.00025	1.2185 ± 0.0022	2.0695 ± 0.0005	2.8166 ± 0.0007
276.73	1.0599 ± 0.0004	1.2255 ± 0.0034	2.0726 ± 0.0007	2.8231 ± 0.0011

Table 11: Example defining the restricted exchange model of $\sim 1M$ glucose at temperature of 269.5 K

T=269.5K	water‡	C_6	$C_{3/4\alpha}$	$C_{3/4\beta}$	$C_{3/4\alpha}$	$C_{3/4\beta}$	$C_{2,\alpha}$	$C_{2,\beta}$	$C_{1,\alpha}$	$C_{1,\beta}$
δ [PPM]	0.0	0.78885	1.2299	1.37237	1.2198	1.43349	1.07148	1.25909	2.06430	2.81704
k_{ex} [Hz]	calculated	372.2	207.2	207.2	169.0	169.0	177.8	177.8	102.25	242.4
c_{frac}	110.0	1.0	0.36	0.64	0.36	0.64	0.36	0.64	0.36	0.64

‡ The water is used as a reference, therefore the chemical shift is zero. Exchange rate of water is calculated from the other exchange rates on the fly, so there is no need to specify it in the model. The fractional concentration of water is here set as 110 mol/l (of water hydrogen atoms). For confidence intervals of these paremeters, see Tables 1, 6 and 7, from which this table is assembled. Note however, that the chemical shifts correspond to the temperature of ~295 K since no extrapolation of those was attempted.

Table 12: Example defining the restricted exchange model for fitting at low temperature (270 K) and pH=6.21

	water‡	C_6^a	$C_{3/4\alpha}$	$C_{3/4\beta}$	$C_{3/4\alpha}$	$C_{3/4\beta}$	$C_{2,\alpha}$	$C_{2,\beta}$	$C_{1,\alpha}$	$C_{1,\beta}$
δ [PPM]	0.0	0.70-0.80	1.15-1.5	1.15-1.5	1.15-1.5	1.15-1.5	1.0-1.1	1.15-1.5	1.9-2.2	2.7-3.0
k_{ex} [Hz]	calculated	20-1950	20-1950	$\equiv\leftarrow^b$	20-1950	$\equiv\leftarrow$	20-1950	$\equiv\leftarrow$	20-1950	20-1950
c_{frac}	110.0	1.0	0.36	0.64	0.36	0.64	0.36	0.64	0.36	0.64

^{a} Suitably narrow range is used where the assignment is obvious. The chemical shift can be estimated from the spectrum. ^{b} With the sign we indicate, that in this (restricted) model, the exchange rate of β is set equal to the α on the left in the table. This way, the chemical shifts of corresponding α and β (of the same carbon) are obtained during the fitting.

Table 13: Example defining the restricted exchange model for fitting at higher temperature (295 K), at pH=6.21. The chemical shift ranges are narrowed arround the values obtained at low temperature to prevent swapping.

	water‡	C_6^a	$C_{3/4\alpha}$	$C_{3/4\beta}$	$C_{3/4\alpha}$	$C_{3/4\beta}$	$C_{2,\alpha}$	$C_{2,\beta}$	$C_{1,\alpha}$	$C_{1,\beta}$
δ [PPM]	0.0	0.70-0.80	1.15-1.25	1.30-1.40	1.20-1.22	1.4-1.5	1.0-1.1	1.2-1.3	1.9-2.2	2.7-3.0
k_{ex} [Hz]	calculated	20-1950	20-1950	$\equiv\leftarrow^b$	20-1950	$\equiv\leftarrow$	20-1950	$\equiv\leftarrow$	20-1950	20-1950
c_{frac}	110.0	1.0	0.36	0.64	0.36	0.64	0.36	0.64	0.36	0.64

Table 14: Parameters of the Eyring equation for exchange rates obtained by linear fit of the linearized Eying equation. Note that parameters for pH=7.38 should be used only in low temperatures for estimates of exchange rates.

pH	6.21	
site/parameter	κ	$\Delta G^{\ddagger}$ [J/mol]
C_6	1.3749-06	2.22730e+04
$C_{3/4}$	1.5401-06	2.38401e+04
$C_{3/4}$	2.4989-05	3.05403e+04
C_2	8.2092-06	2.79324e+04
$C_{1,\alpha}$	1.6195-02	4.61731e+04
$C_{1,\beta}$	1.2615-03	3.85195e+04

pH	7.00	
site/parameter	κ	$\Delta G^{\ddagger}$
C_6	1.4289-05	2.81024e+04
$C_{3/4}$	5.9687-05	3.19200e+04
$C_{3/4}$	2.2834-03	4.00640e+04
C_2	2.3088-04	3.50790e+04
$C_{1,\alpha}$	2.4681-01	4.96645e+04
$C_{1,\beta}$	7.1153-02	4.54870e+04

pH	7.38	
site/parameter	κ	$\Delta G^{\ddagger}$
C_6	4.1509-02	4.55072e+04
$C_{3/4}$	1.0688+03	6.82804e+04
$C_{3/4}$	7.9775-04	3.67809e+04
C_2	2.1281+03	7.00003e+04
$C_{1,\alpha}$	5.7136+02	6.54245e+04
$C_{1,\beta}$	2.8559+00	5.23164e+04
